# Camptothecin-PHA nanoparticles attenuate drug-induced gut microbiome dysbiosis and metabolic toxicity

**DOI:** 10.3389/fmicb.2025.1617468

**Published:** 2026-01-28

**Authors:** Shanshuo Liu, Benchen Rao, Wenjie Liu, Xuemei Wang, Haiyu Wang, Liwen Liu, Guizhen Zhang, Junyi Sun, Lei Li, Daixu Wei, Zujiang Yu, Zhigang Ren

**Affiliations:** 1 Department of Infectious Diseases, State Key Laboratory of Antiviral Drugs, Pingyuan Laboratory, The First Affiliated Hospital of Zhengzhou University, Zhengzhou, China; 2 Gene Hospital of Henan Province, Precision Medicine Center, The First Affiliated Hospital of Zhengzhou University, Zhengzhou, China; 3 Department of Pain Management, The First Affiliated Hospital of Zhengzhou University, Zhengzhou, China; 4 Zigong Institute of Brain Science, Zigong Psychiatric Research Center, Zigong Affiliated Hospital of Southwest Medical University, Zigong, China; 5 Key Laboratory of Resource Biology and Biotechnology in Western China, Ministry of Education, School of Medicine, Department of Life Sciences and Medicine, Northwest University, Xi'an, China

**Keywords:** camptothecin, gut microbiome, nanoparticles, PHBVHHx, serum metabolism

## Abstract

**Introduction:**

The anticancer drug camptothecin (CPT) has limited clinical applications due to severe toxic reactions.

**Methods:**

We combined CPT with PHBVHHx (PHA) nanoparticles by a modified emulsion method for the first time construct a novel nanomedical drug (CPT-PHA-NPs, CPNs).

**Results:**

*In vitro* experiments verified the drug loading level (89%), sustained-release properties (40% release within 48 h; near-complete release over 21 days), and inhibition ability of the compound on HT-29 cell activity (IC_50_ = 0.44 μM). *In vivo*, CPN-treated mice showed significantly less body weight reduction (*P* < 0.05 from day 7) and markedly improved liver and kidney function markers compared to controls. Histological analysis confirmed that CPN effectively prevented hepatocyte necrosis and renal inflammation observed with free CPT, demonstrating higher biosafety and lower toxicity. Crucially, 16S rRNA sequencing revealed that CPT severely depleted probiotics (*Akkermansia, Lactobacillus, Candidatus_Arthromitus*, and *Bacilli_unclassified*) while promoting pathogenic taxa (*Lachnospiraceae_NK4A136_group, [Eubacterium]_xylanophilum_group*, and *Faecalibaculum*), whereas CPNs attenuated these microbial disruptions. Metabolomics further showed CPNs' milder effects on phenylalanine and essential amino acid metabolism vs. CPT.

**Discussion:**

In conclusion, this novel type of nanomaterial not only possesses excellent performance but also can reduce the impact of CPT on tissues, intestinal flora and serum metabolism, providing a new strategy for anti-tumor treatment that takes into account both microbial homeostasis and metabolic safety.

## 1 Introduction

Cancer chemotherapy, while indispensable for tumor eradication, often inflicts severe collateral damage on host physiology—particularly the gut microbiota and its intricate metabolic network. Mounting evidence reveals that chemotherapy-induced dysbiosis not only exacerbates treatment toxicity but may also compromise therapeutic efficacy, creating an urgent need for anticancer strategies that concurrently preserve microbial homeostasis. Camptothecin (CPT) is a pentacyclic monoterpene alkaloid isolated from the bark and stem of *Camptotheca acuminata*, consisting mainly of a planar pentacyclic structure and a lactone ring with a chiral center (Khaiwa et al., 2021). As a topoisomerase I inhibitor, CPT exerts cytotoxicity by stabilizing the enzyme-DNA cleavage complex through its lactone ring and C-20-OH group, ultimately inducing lethal DNA damage in replicating cells (Martino et al., 2017). However, the lactone ring is susceptible to hydrolyzing and converts into inactive carboxylate at physiological pH conditions, which has a high affinity for human serum albumin and significantly reduces cellular uptake (Mi and Burke, 1994). Beyond these pharmacokinetic limitations, CPT's clinical utility is further hampered by its severe hepatotoxicity, nephrotoxicity, and gut dysbiosis (Laskar et al., 2014).

These limitations directly led to the emergence of derivatives of CPT, which increased clinical utility by improving their pharmacological and pharmacokinetic properties. For example, topotecan and irinotecan have been approved for clinical use by the Food and Drug Administration (FDA), the former for the treatment of ovarian and lung cancer, and the latter for the treatment of colorectal cancer (Hevener et al., 2018; Singh et al., 2020; Pommier, 2006; Liu et al., 2015). Although these two CPT derivatives had largely improved the availability of CPT, they still had significant side effects, namely myelosuppression and gastrointestinal toxicity (Chen et al., 2020a). Recent studies suggest that chemotherapy-induced gut microbiota disruption plays a pivotal role in these toxicities, as depletion of beneficial microbes (e.g., *Lactobacillus*) and enrichment of opportunistic pathogens exacerbate intestinal barrier dysfunction and systemic inflammation. Thus, beyond traditional toxicity metrics, preserving microbial- metabolic homeostasis represents a novel paradigm for evaluating chemotherapy safety (Bailly, 2019).

Another solution is to encapsulate CPT in nanoparticles, which not only prolong and maintain appropriate drug levels in plasma, but also improve the pharmacokinetic properties and increase their residence time in close contact with the absorption membrane (Huarte et al., 2021). This drug delivery system is not unusual. Chemotherapy medications are commonly loaded onto nanoparticles, ensuring more precise drug delivery while maintaining superior anti-tumor capabilities. Nevertheless, there are significant limitations in clinical applications due to poor biocompatibility, high immunogenicity, expensive production costs, and limited source materials (Shi et al., 2017). Crucially, their impact on the gut microbiome and host metabolism remains poorly understood, despite growing recognition of the microbiota's role in drug efficacy and toxicity.

Recent studies have indicated that minimizing disruption to the gut microbiota has become an important consideration in the design of anticancer nanoformulations. Natural-derived nanocarriers are increasingly favored in this context, as they can better balance targeted drug delivery with microbial homeostasis compared to synthetic counterparts (Teng et al., 2025; Huang et al., 2025; Han et al., 2025). However, few such carriers have been specifically developed to address chemotherapy-induced gut microbiota disruption, leaving a critical gap in current nanomedicine strategies.

To address these gaps, we designed a microbiome-friendly nanocarrier based on poly(3-hydroxybutyrate-co-3-hydroxyvalerate-co-3-hydroxyhexanoate) (PHBVHHx, a polyhydroxyalkanoate (PHA) tripolymer). PHAs are uniquely suited for mitigating chemotherapy-induced microbial dysbiosis due to their inherent biodegradability and reported prebiotic-like properties. Structurally, PHBVHHx offers a porous architecture for high drug loading, while its closed internal environment ensures stability (Hu et al., 2020). In this study, we developed a novel camptothecin nanoformulation (CPT-PHA-NPs, CPNs) and established a comprehensive safety evaluation framework integrating microbiome and metabolomics analyses. Importantly, this study provides the first demonstration that PHBVHHx-based nanoencapsulation actively maintains gut microbial homeostasis during chemotherapy—a novel finding beyond the functions of traditional drug delivery systems. Beyond conventional delivery metrics, we revealed the unique dual protective mechanisms of CPNs: on one hand, the nanoencapsulation reduces direct toxic impact on intestinal tissues; on the other hand, it preserves probiotic communities and maintains metabolic balance, thereby indirectly alleviating chemotherapy-induced systemic dysregulation. This microbiome-friendly nanocarrier strategy offers a new paradigm for evaluating nanomedicine safety from the perspective of microbiome-metabolome interactions and opens new avenues for developing cancer therapies with low ecological toxicity.

## 2 Methods

### 2.1 Materials and preparation of nanoparticles

Medical grade poly(3-hydroxybutyrate-co-3-hydroxyvalerate-co-3-hydroxyhexanoate) (PHBVHHx, or PHA) of Mw = 48 kDa was obtained from Bluepha Microbial Technology Co., Ltd. (Beijing, China). Poly(vinyl acetate) (PVA, 89% hydrolyzed with an average Mw of 13,000–23,000) and camptothecin were both purchased from Sigma-Aldrich (USA). The PHA nanoparticles loaded with CPT (CPNs) were designed and prepared by a modified emulsion method (Hu et al., 2020). Briefly, 100 mg of CPT and 1 g of medical PHA were both dissolved in 40 mL of dichloromethane (DCM) for 6 h. The solution was then poured into the 100 mL aqueous phase of 1% (w/v) PVA and treated with ultrasound (1,200 W) for 5 min to form an emulsion in ice water. The emulsion was treated by rotary evaporation to evaporate DCM and solidify the CPNs. To remove residual PVA and unpackaged CPT, the nanoparticles were centrifuged at 12,000 rpm for 10 min, washed in ddH_2_O three times, and dispersed finally in phosphate buffered saline (PBS). As controls, the pPNs were produced via a similar method without CPT introduction. For fluorescent labeling, 0.01% rhodamine B 5-isothiocyanate (RBITC) was added to the DCM phase during preparation.

### 2.2 Physicochemical characterization of nanoparticles

The morphology of the CPNs and pPNs was investigated by TEM on a JEM 1200EX instrument (JEM, Japan). The size and surface charge were detected using a Malvern Zetasizer Nano-zs90 (UK) operating at a laser wavelength of 633 nm and a scattering angle of 90° at 20 °C.

### 2.3 CPT entrapment efficiency of nanoparticles

The CPT entrapment efficiency of the CPNs was detected referring to the previous method (Hu et al., 2020). Briefly, after stirring in the preparation process of CPNs, the supernatant was collected for CPT measurement after centrifugation at 12,000 rpm for 10 min. The free-CPT (fC) amount (W1) in the supernatant, which was not packaged into nanoparticles, was analyzed by a Scientific Varioskan Flash spectrophotometer (Thermo-Fisher, USA) at an absorbance of 378 nm. A serial concentration of CPT dissolved in 1% PVA solution was used for the standard curve. The amount of CPT loaded was achieved by subtracting that of unloaded CPT (W1) from the total amount (W0).

Therefore, the loading efficiency of CPT was calculated using the following equation:


$$\begin{array}{l} Entrapment\;Efficiency\;\left(\%\right)=\left(W0-W1\right)/W0*\;100\%, \end{array}$$

Where W0 is the total CPT in preparation of CPNs, W1 is the free-CPT and (W0–W1) is the packaged CPT in nanoparticles.

### 2.4 Degradation of nanoparticles

1 g of CPNs or pPNs was immersed in 40 mL simulated intestinal juice (*pH* 8.0) containing 0.1% trypsin. After shaking at 50 rpm in a 37 °C shaker for the indicated time, 5 mL of suspending NPs were centrifuged at 12,000 rpm for 5 min for collection of nanoparticles. After freeze-drying, the weight of the nanoparticles (M1) was determined.

The degradation rate was calculated as:


$$\begin{array}{l} Degradation\;rate\;\left(\%\right)=\left(M0-M1\right)/M0*100\%, \end{array}$$

Where M0 is the initial weight of nanoparticles and M1 is the weight of residual nanoparticles after degradation.

### 2.5 *In vitro* release of CPT

Similarly, 200 mg of CPNs were immersed in 10 mL simulated intestinal juice (*pH* 8.0) containing 0.1% trypsin. After shaking at 50 rpm in a 37 °C shaker for the indicated time, the suspension was centrifuged at 12,000 rpm for 5 min. The amount of released CPT in the supernatant was measured by a Scientific Varioskan Flash spectrophotometer. After each sampling, an equal volume of fresh pre-warmed release medium was added to the sediment, which was then re-suspended and returned to the shaker. The cumulative released CPT (W2) was calculated considering dilution factors from medium replacement.

The CPT release rate was calculated using the equation:


$$\begin{array}{l} Cumulative\;release\;rate\left(\%\right)=W2/W3*100\%, \end{array}$$

Where W3 is the total amount of CPT loaded in the CPNs used for the release test.

### 2.6 *In vitro* cytotoxicity

HT-29 cells were seeded at 5,000 cells per well in a 96-well plate. To measure the correlation between cell viability and CPT content, cells were treated with CPNs and fC at CPT concentrations ranging from 0 to 1,000 μM. The pPNs were added at equivalent PHA concentrations. The group containing cells with pure DMEM medium was defined as the 100% cell viability control. Following incubation for 48 h, the cell viability was quantified by CCK-8 assay using a Cell Counting Kit-8 (CCK-8, FANBO, China). Subsequently, based on optimized CPT concentration, HT-29 cells were treated with CPNs, pPNs, and fC, and cell viability was assessed on days 0, 1, 4, and 7.

### 2.7 *In vitro* cellular uptake

HT-29 cells, typical human colon cancer cells, were cultured in DMEM medium (HyClone) containing 10% FBS (Gibco) and 1% penicillin/streptomycin (HyClone) at 37 °C in 5% CO_2_. Cells were seeded in 24-well plates at a density of 5,000 cells per well overnight. The cells were co-incubated with nanoparticles loaded with RBITC at an equivalent concentration of 0.1 μM CPT in 1 h. Then, the cells were treated by washing with PBS three times, fixation with 4% paraformaldehyde in 15 min, and stained with 1 μg/mL Calcein-AM for cytoplasm and 1 μg/mL DAPI 4′,6-diamidino-2-phenylindole) for nucleus for 15 min. The labeled cells were observed under a confocal laser scanning microscope (CLSM, Leica, TCS SP5, Germany).

### 2.8 *In vivo* study

This study was approved by the Ethics Committee of the First Affiliated Hospital of Zhengzhou University (2021-KY-0716-003) and complied with the Animal Research Reporting of *in vivo* Experiments (ARRIVE) guidelines. The study was carried out in accordance with the National Institutes of Health guide for the care and use of Laboratory animals. The laboratory animal facility has been accredited by AAALAC (Association for Assessment and Accreditation of Laboratory Animal Care International). 9-week-old C57BL/6 male mice (20–25 g) were purchased from Beijing Vital River Laboratory Animal Technology Co., Ltd. (Beijing, China). These 35 mice were housed at the Laboratory Animal Center of Zhengzhou University. Mice were free to obtain food and tap water and were kept on a 12-h dark/light cycle. All mice were randomly divided into 4 groups: control group (*n* = 5), pPNs group (pure PHA nanoparticles, *n* = 10), fC group (free camptothecin, *n* = 10) and CPNs group (CPT-PHA-NPs, *n* = 10). The CPT dose was 0.5 mg per administration. Drugs were administered by oral gavage on days 0, 2, 4, 6, 8, 10, 12, and 14, with a recovery period from day 15 to 21.

### 2.9 Sample collection

Stool and venous blood (0.5 mL, collected from the retro-orbital plexus) were collected on day 0, day 14, and day 21. Venous blood was collected after fasting for 12 h and centrifuged immediately (4,000 rpm, 4 °C, 5 min). The serum was stored in a −80 °C refrigerator for metabolomics study. Fresh tail stool samples were collected and stored in a −80 °C refrigerator immediately after packaging for intestinal microbiota sequencing and analysis. All samples left at room temperature for more than 2 h were discarded. At the end of the experiment, the animals were euthanized. All animal surgeries were performed with humane care and protocols adhered to institutional guidelines. At the same time, the liver, kidney, small intestine, and colon tissues of mice were collected on day 21 and fixed with 4% paraformaldehyde.

### 2.10 Immunohistochemical staining of tissues

After fixation with paraformaldehyde, the liver, kidney, small intestine, and colon tissues were treated by dehydration and embedding in paraffin. Sections were cut for hematoxylin and eosin (H&E) staining (Solarbio, China). Meanwhile, sections were also stained by DAPI for *in-situ* observation of nanoparticles loaded with RBITC as described previously (Zhao et al., 2021b).

### 2.11 DNA extraction of fecal samples

Each fecal sample with frozen aliquots was processed by phenol-chloroform DNA extraction using a bead beater to mechanically disrupt cells, followed by phenol–chloroform extraction (Ren et al., 2017). DNA extraction was performed as previously described (Qin et al., 2014). The DNA concentration was detected by a NanoDrop (Thermo Scientific), and the molecular size was estimated by agarose gel electrophoresis.

### 2.12 PCR amplification and MiSeq sequencing

Extracted bacterial DNA was amplified with a pair of primers targeting the variable V3-V4 region (341F/805R) of the 16S rRNA gene. The forward primer is 5′-CCTACGGGNGGCWGCAG-3′; and the reverse primer is 5′-GACTACHVGGGTATCTAATCC-3′. PCR amplification and DNA library construction were done as described in a previous study (Ren et al., 2017). Sequencing and bioinformatics analysis were carried out by Shanghai Mobio Biomedical Technology Co., Ltd. in China on the Miseq platform (Illumina Inc., USA). The original Illumina read data for all samples has been deposited and the accession number is PRJNA972503.

### 2.13 Sequence data process

Amplicon reads were processed strictly as follows: a) paired-end sequenced reads of each library were overlapped by FLASH version 1.2.10 (Magoč and Salzberg, 2011) with default parameters, b) using custom functions to perform more specific quality control on overlapping reads generated by FLASH, c) de-multiplexing the reads and assigning them to different samples according to barcodes, and d) detecting and removing chimeric sequences with UCHIME version 4.2.40 (Edgar et al., 2011). We used the 16S “gold” database provided by the Broad Institute as a reference (version microbiome util-r20110519, http://drive5.com/uchime/gold.fa) to match operational taxonomic units (OTUs).

### 2.14 OTU analysis and taxonomy annotation

Random reads of all samples with equal numbers were chosen, and the UPARSE pipeline classified OTUs with the following steps (Edgar, 2013): (a) duplicate sequences and unique elements were first eliminated; (b) the unique sequences were grouped into OTUs with the command “search-cluster_otus”; and (c) the randomly chosen sequences were aligned with the OTU sequences with the command “search-usearch_global-id 0.97”, the identity threshold was set to 0.97, and then the OTU composition table was created. The sequences were annotated using the RDP classifier version 2.6 (Wang et al., 2007), and the confidence level was set at 0.5 according to the developer documents.

### 2.15 Analysis of the gut microbial diversity and the distance of the bacterial community

The richness and diversity of microbiota were presented by the Shannon index. Bacterial community distance analysis was performed by the NMDS, PCA, and PCoA to visualize interactions between bacterial communities. Heatmaps were drawn with Heatmap Builder. The fecal microbial characterization was analyzed using the LEfSe method (http://huttenhower.sph.harvard.edu/lefse/). Significantly different taxa in LEfSe analysis were identified using the Kruskal-Wallis rank sum test, and LDA evaluated the effect size of each trait. To illustrate the functional profile of fecal microflora, Kyoto Encyclopedia of Genes and Genomes (KEGG) orthology, and KEGG pathway/module profiles were created by the PICRUSt pipeline (Langille et al., 2013) and human version 0.99 (Abubucker et al., 2012). The PICRUSt method was used to predict gene family abundance in host-related communities, incorporating data from the Human Microbiome Project.

### 2.16 Metabolomics analysis

We analyzed the serum metabolomics of mice using UPLC-MS. Mass spectrometry separation was conducted using a Waters Acquity I-Class UPLC (Waters Corporation, Milford, MA), with system stability monitored by mixed quality control (QC) samples. For quality control and validation, mixed QC samples were injected throughout the analytical sequence at intervals of every 8 experimental runs to monitor system stability. Data quality was evaluated based on the relative standard deviation (RSD) of metabolic features in the QC samples; metabolites with a peak area RSD > 30% were excluded. The median RSD for all retained metabolites was 12%, indicating good analytical precision. To validate the supervised multivariate statistical model, a 200-permutation test was performed, confirming that the OPLS-DA model was not overfitted (Q^2^ intercept < 0). Metabolite identification was achieved by matching the accurate mass (mass error < 5 ppm) and MS/MS spectra against the HMDB database.

Detailed LC and MS methods followed established protocols (Boysen et al., 2018): (a) For targeted metabolomics, a Waters Xevo TQ-S triple quadrupole (TQS) with electrospray ionization (Iglesias et al., 2018) was used in the selected reaction monitoring mode (SRM). (b) For most metabolites, two SRM transitions were used based on the largest peak area. (c) For non-targeted analysis, Thermo QExactive HF (QE) with ESI was used. Stoichiometric analysis was performed by SIMCA-P 11.0 (Umetrics AB, Umeå, Sweden). A multivariate pattern recognition technique of PLS-DA was performed. Select PLS-DA parameters for evaluation. R2Y and Q2Y represent the goodness of fit and predictive power of the model.

### 2.17 Statistical analysis

Statistical analysis was performed using SPSS version 22.0 (SPSS Inc., Chicago, IL). For normally distributed data, differences between groups were compared using one-way ANOVA. For non-normally distributed data, the Wilcoxon rank-sum test was applied. Significance was accepted when the *p*-value was less than 0.05.

## 3 Results

### 3.1 Nanoparticle conjugation of CPNs and its characterization

**Figure 1:**
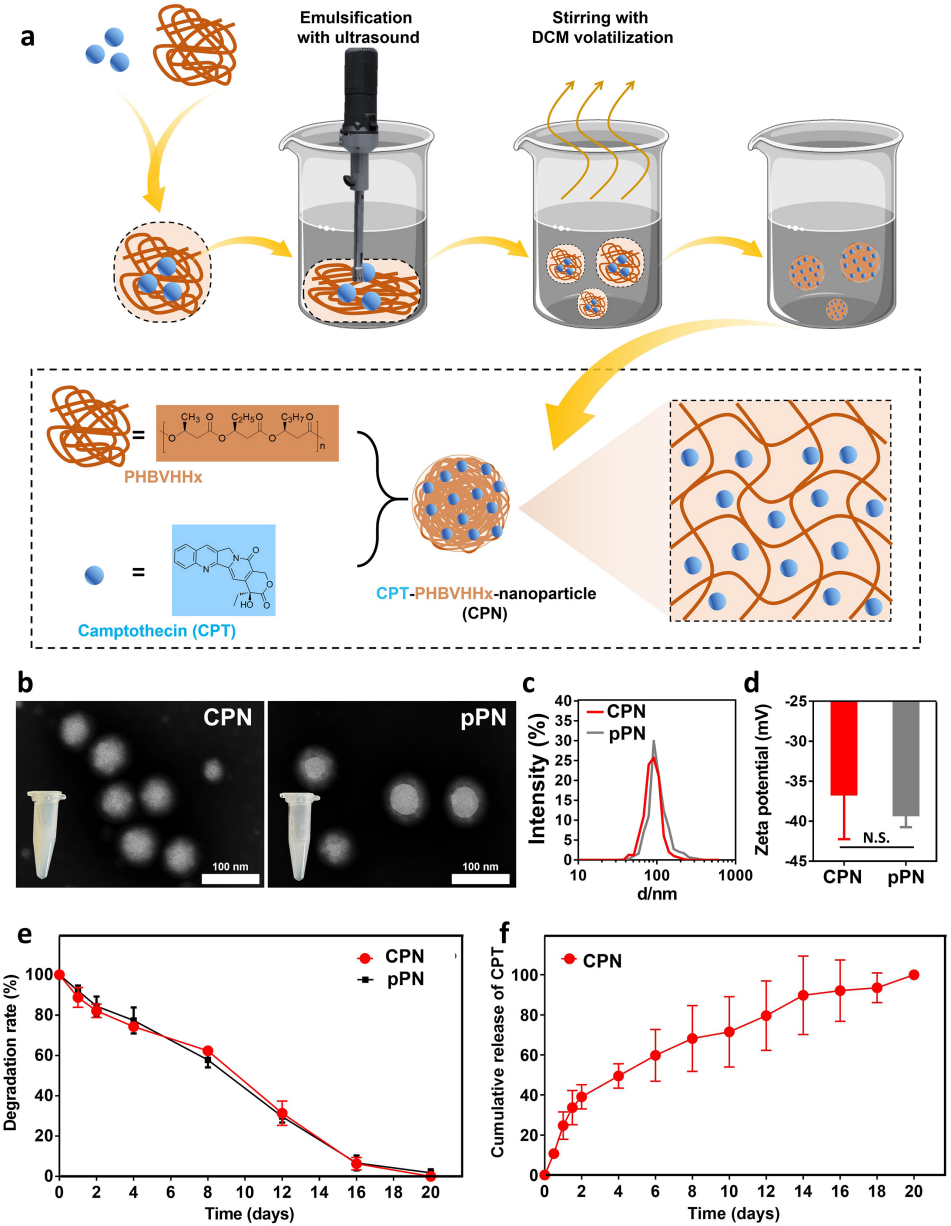
Preparation and characterization of CPNs. **(a)** Schematic illustration of the CPNs preparation process and structure. Comparison of appearance and TEM images **(b)**, size distributions **(c)**, Zeta potential **(d)**, and degradation rate **(e)** of CPNs and pPNs, respectively. **(f)** Cumulative release of CPT from CPNs in 21 days. Data in **(d–f)** are from three independent replicates and presented as mean ± SD. CPNs, PHA nanoparticles loaded with CPT; pPNs, pure PHA nanoparticles; TEM, transmission electron microscopy.

The prepared CPNs and pPNs (Figure 1a) both formed a white suspension. They exhibited similar nano-sized morphology in TEM images (Figure 1b), with an average diameter of 80 nm (Figure 1c) and surface charges ranging from−43 mV (pPNs) to −30 mV (CPNs) (Figure 1d). The loading efficiency of CPT in PHA was 89%. With continuous degradation of PHA in simulated intestinal juice, CPT was released as an initial burst of approximately 40% within 48 h and nearly completely released on day 21 from the CPNs as shown in Figure 1f. The degradation curve of pPNs coincided with that of CPNs (Figure 1e), indicating that the drug does not interfere with the degradation of PHA, which is similar to the results of PHA NPs in previous studies (Hu et al., 2020; Zhao et al., 2021b; Chen et al., 2020b).

### 3.2 CPNs inhibited cell proliferation *in vitro*

**Figure 2:**
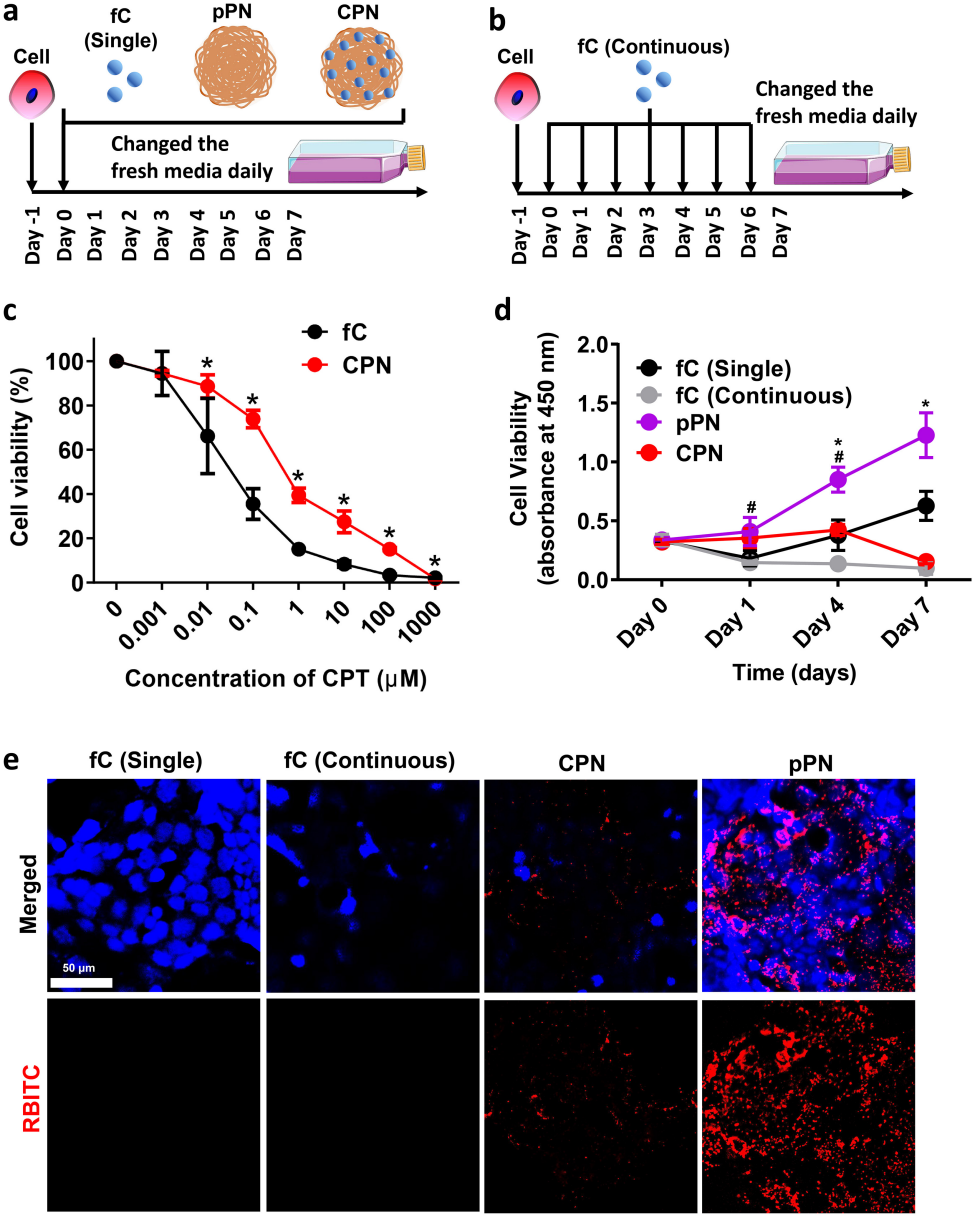
*In vitro* cell experiments with different modes of administration of CPNs and controls. **(a)** Schematic of cells treated with single drug-supplying style for fC, CPNs, and pPNs. **(b)** Schematic of cells treated with continued drug-supplying style in fC. **(c)** Cell viability of HT-29 cells treated with fC and CPNs with different concentrations of CPT in 24 h. **(d)** Cell viability of HT-29 cells treated with fC (single), fC (continuous), pPNs, and CPNs with 0.1 μM CPT for 0 day, 1 day, 4 days, and 7 days, respectively. **(e)** *In-situ* observation of HT-29 cells treated with fC (single), fC (continuous), pPNs, and CPNs for 7 days, respectively. 3 parallel studies were conducted for each sample in **(c)** and **(d)**. fC, free-camptothecin; CPNs, PHA nanoparticles loaded with CPT; pPNs, pure PHA nanoparticles; RBITC, rhodamine B 5-isothiocyanate. In figure **(d)**, * indicates a significant difference (*P* < 0.05) between the pPN and CPN groups; # indicates a significant difference between the CPN and fC (continuous) groups.

We evaluated the *in vitro* inhibition of the cellular proliferation of CPNs, pPNs, fC (single), and fC (continuous) using a CCK8 assay for two models (Figures 2a, b): (1) three independent series of experiments were conducted for CPNs, pPNs, and fC, respectively. In each series, cells received a single treatment of the respective formulation at a range of concentrations; and (2) with daily replenishment of fC [fC (Continuous)], and cell viability was assessed at 1, 4, and 7 days post-administration. Notably, fC exhibited significant cytotoxicity to HT-29 cells, with half maximal inhibitory concentrations (IC50 values) of 0.03 μM, while CPNs had IC_50_ values of 0.44 μM (Figure 2c). At 0.1 μM, a statistically significant difference in cell viability was observed between the CPNs (35.45%) and fC (73.82%) groups. After co-culture with NPs, the fresh medium was replaced daily, the intracellular CPNs can continuously release CPT, resulting in a slow decline of cell activity (Figure 2d). One-time dosing of free CPT, namely fC (Single), showed a valley tendency with a decrease on day 1 and increased on day 4 and day 7, indicating residual CPT were removed with fresh medium. Oppositely, continuous CPT supply, namely fC (Continuous), has a significant toxicity and showed low cell activity on day 7. Statistically, at day 7, the cell viability of the CPNs group was comparable to that of the fC (Continuous) group, and both were significantly lower than that of the fC (Single) group (*P* < 0.05).

The toxicity or biocompatibility of the blank nanocarriers is also one of the most important indicators in clinical application. HT-29 cells with a mass of internalized red pPNs can be observed by a confocal laser scanning microscope (CLSM) (Figure 2e). The cell activity of pPNs showed an increasing trend without significant toxicity compared to the control group in Figure 2d, which also explained the non-toxicity of PHA as a nanocarrier. The cell activity of CPNs was seen to be consistently low and showed no significant difference from fC (Continuous) at day 7, with only a small number of cells found in CLSM (Figure 2e). It illustrated the *in vitro* anti-tumor effect of CPNs is an excellent sustained drug release system, equivalent to continuous supplement of anti-cancer drug, and superior to one-time dosing.

### 3.3 CPNs have better biosafety and less toxicity *in vivo*

**Figure 3:**
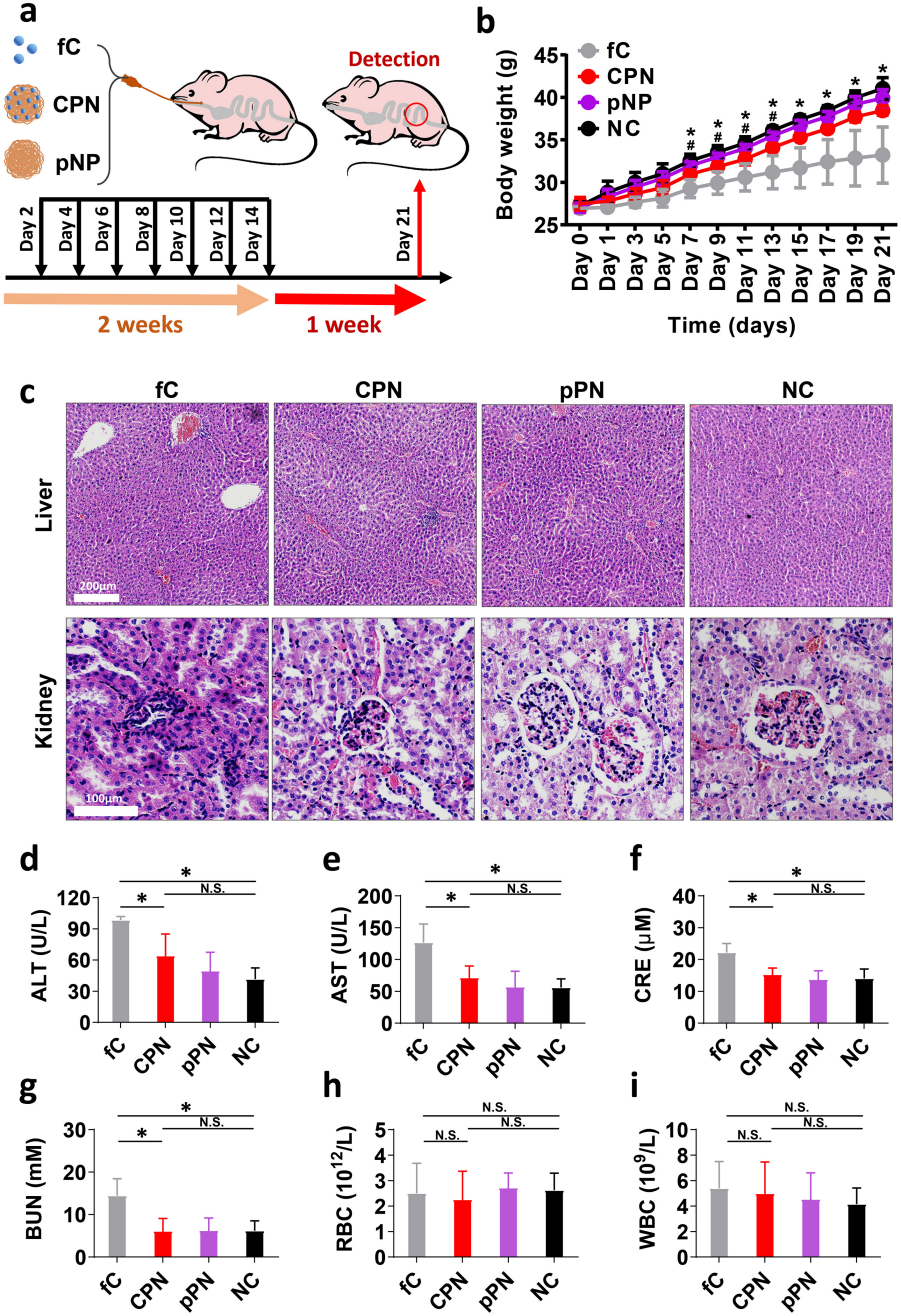
*In vivo* animal test and systematic toxicity evaluation of CPNs and controls. **(a)** Point-in-time of gavage and detection of mice treated by fC, CPNs, and pPNs. **(b)** Body weight changes of mice treated with fC, CPNs, pPNs, and NC, respectively. **(c)** H&E staining of the liver and kidneys on day 21. **(d, e)** Comparison of liver function between different groups. **(f, g)** Comparison of kidney function between different groups. **(h, i)** Hematological parameters of RBCs and WBCs. fC, free-camptothecin; CPNs, PHA nanoparticles loaded with CPT; pPNs, pure PHA nanoparticles; ALT, Alanine transaminase; AST, Aspartate aminotransferase; CRE, Creatinine; BUN, Blood urea nitrogen; RBC, Red blood cell; WBC, White blood cell. In figure **b**, * indicates a significant difference (*P* < 0.05) between the fC and CPN groups; # indicates a significant difference between the CPN and NC groups. In figure **d**, N.S. indicates no significant difference; * indicates *P* < 0.05.

We assessed the biosafety of the CPNs and controls. CPNs and pPNs were gavaged into 9-week-old male mice for 14 days (dosing at 1-day intervals) and we evaluated the *in vivo* behavior of these nanoparticles on the 21st day (Figure 3a). As shown in Figure 3b, because of the constant stimulation of the free anticancer drug CPT, the mice in the fC group lost weight significantly from Day 3 onwards compared to the normal control (NC) group (*P* < 0.05). Furthermore, a statistically significant reduction in the fC group's body weight was observed vs. the CPNs group starting at Day 7 (*P* < 0.05). In contrast, the weight of mice in the CPNs group was lower than that of the NC group, but the decrease was not as pronounced as in the fC group, possibly because the slow release of the drug after coupling with the nanomaterial avoided the damage and weight changes caused by the toxicity and side effects of the drug. Importantly, no significant differences in body weight were observed at any time point among the NC, pPNs, and CPNs groups, demonstrating the safety of both the nanoparticle carrier and the loaded drug.

H&E staining of the liver demonstrated that hepatic lobule disorder and hepatocyte necrosis were widely distributed in the liver treated with fC, whereas few necrotic cells were observed in the CPNs and pPNs groups (Figure 3c). The clinical biochemical parameters of alanine aminotransferase (ALT) and aspartate aminotransferase (AST) for liver function after treatment with CPNs were lower than those in the fC group, and close to those in the pPNs and NC groups (Figures 3d, e). In addition, there was also a significant difference between CPNs and fC groups regarding kidney function, including serum creatinine (CRE) and blood urea nitrogen (BUN) levels (Figures 3f, g). H&E staining showed that the kidneys of the mice treated with free CPT exhibited severe glomerular mesangial cell proliferation, inflammatory cell infiltration, and capillary loop compression. These abnormal histological phenomena did not appear in the groups CPNs, pPNs, and NC (Figure 3c). However, there were no significant differences among the treatment groups regarding the hematology of white blood cells (WBCs) and red blood cells (RBCs) (Figures 3h, i). To sum up, CPNs have better biological safety and less toxicity than free CPT and may be a better choice for anti-cancer therapy.

**Figure 4:**
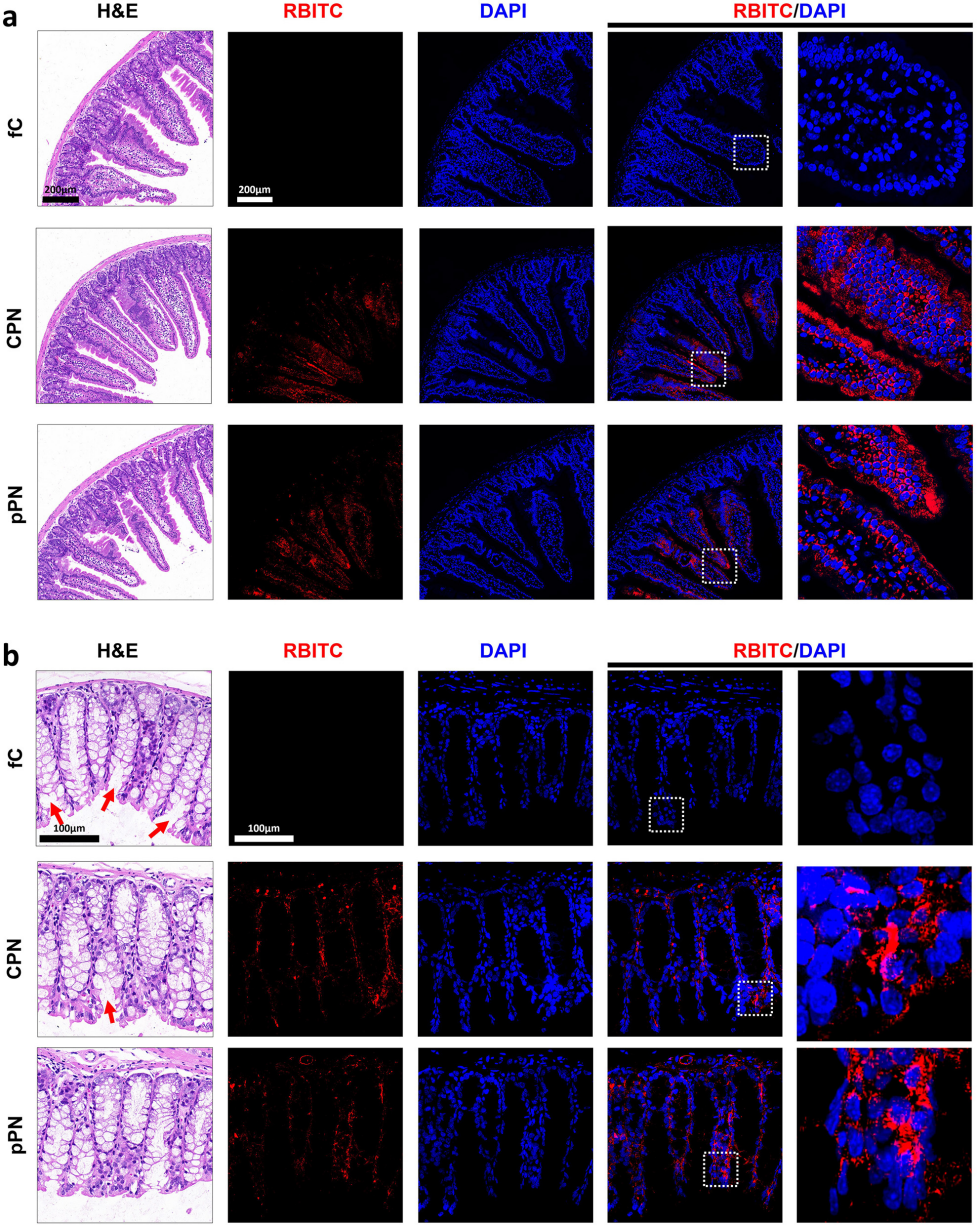
*In-situ* observation of CPNs in small intestine **(a)** and colon **(b)**. Cell nuclei were stained with DAPI in blue; CPNs and pPNs loaded with RBITC showed red. fC, free-camptothecin; CPNs, PHA nanoparticles loaded with CPT; pPNs, pure PHA nanoparticles; DAPI, 4′,6-diamidino-2-phenylindole; RBITC, rhodamine B 5-isothiocyanate.

For oral drugs, the histology of the small intestine and colon is important when investigating the therapeutic efficacy of nanoparticle treatments, was assessed by H&E staining and immunohistochemistry of tissue sections for *in-situ* observation of nano-carriers. As shown in Figures 4a, b, small intestine and colon tissues in the fC group exhibited clear signs of inflammation, including goblet cell depletion, epithelial disruption, and significant infiltration of inflammatory cells into the mucosa. In contrast, tissues from the groups CPNs and pPNs showed no sign of inflammation or disruption of healthy tissue morphology. Both nanoparticles were observed in the intervillus space of the small intestine and colon tissues (red parts) and further diffused into the bloodstream to release the drug CPT with PHA-degradation (Chen et al., 2020b). The accumulation and retention of drugs in the gut cannot be ignored, and it may greatly affect the proportion and activity of gut microbes.

### 3.4 CPNs had a lower effect on the gut microbiome of mice than that of fC

Studying the gut microbiota may have important significance for cancer treatment and prognosis. Therefore, we also looked at the intestinal flora of mice to evaluate the properties of nanomedicine from the perspective of omics. We collected fresh feces of mice at baseline (week 0), at the end of administration (week 2), and at the end of the experiment (week 3) for 16S rRNA sequencing. The gut microbiome of the pPNs did not change significantly and was highly similar to that of the normal control (NC) (Supplementary Figures 1a, j and Supplementary Datas S1–S3). In contrast, the non-metric multidimensional scale (NMDS) analysis and principal coordinate analysis (PCoA) (Supplementary Figures 2a, b) showed that the gut microbiome of the fC was significantly different from the baseline after 2 weeks of administration, and approached the baseline level again at week 3, but did not completely return to normal. The heatmap showed the abundance of 29 key operational taxonomic units (OTUs) in each sample across the three groups (Supplementary Figure 2c and Supplementary Data S4).

**Figure 5:**
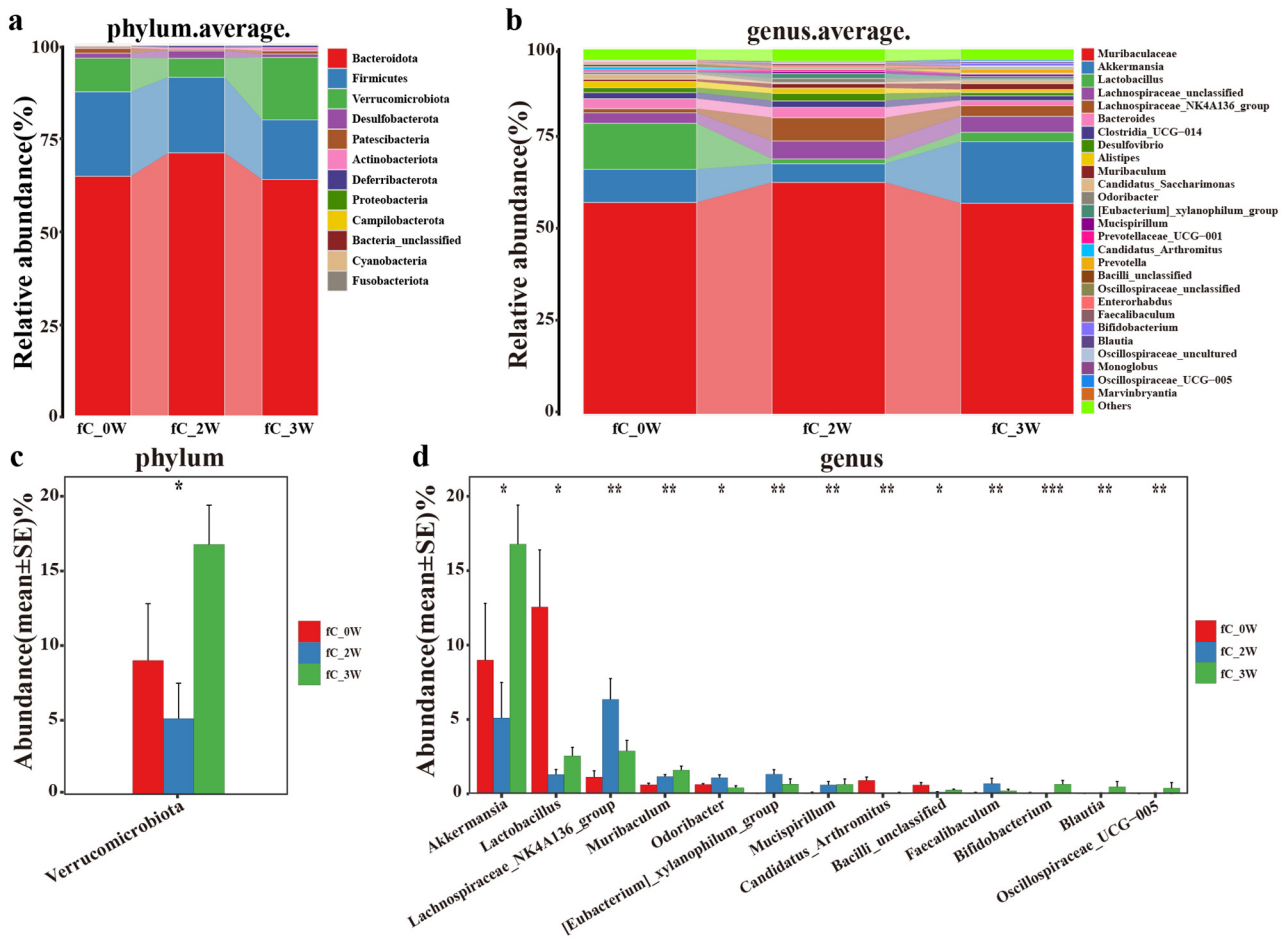
Average composition and comparison of gut microbes at baseline, week 2, and week 3 in the fC group (*n* = 10). Average composition of the gut microbiome at three different points in time at the phylum level **(a)** and the genus level **(b)**. Comparison of intestinal microbial abundance at the phylum level **(c)** and the genus level **(d)** at the three different points in time. fC, free-camptothecin. *P < 0.05, **P < 0.01, ***P < 0.001.

We then analyzed the average composition of gut microbes at different levels at three time points in the fC group. At the phylum level, *Bacteroidota, Firmicutes*, and *Verrucomicrobiota* accounted for more than 96% of the total, which belonged to the dominant phylum (Figure 5a and Supplementary Data S5). At the genus level, *Muribaculaceae, Akkermansia*, and *Lactobacillus* were the dominant genera (Figure 5b and Supplementary Data S6). We compared bacterial abundance to identify key bacterial communities that had changed, and found that the abundance of *Verrucomicrobiota* decreased significantly after administration, and increased during the recovery period after withdrawal of the drug (Figure 5c and Supplementary Data S7). At the genus level, 13 genera showed significant changes in abundance (Figure 5d and Supplementary Data S8). In addition, the linear discriminant analysis (LDA) effect size (LEfSe) method was utilized to select the greatest differences in taxa. A representative cladogram showed the phylogenetic distribution of dominant microorganisms in mice at different time points after fC gavage (Supplementary Figure 2d). The LDA score plot showed the significant differences between groups of bacteria at the genus level (LDA > 2.5, *P* < 0.05) (Supplementary Figure 2e).

**Figure 6:**
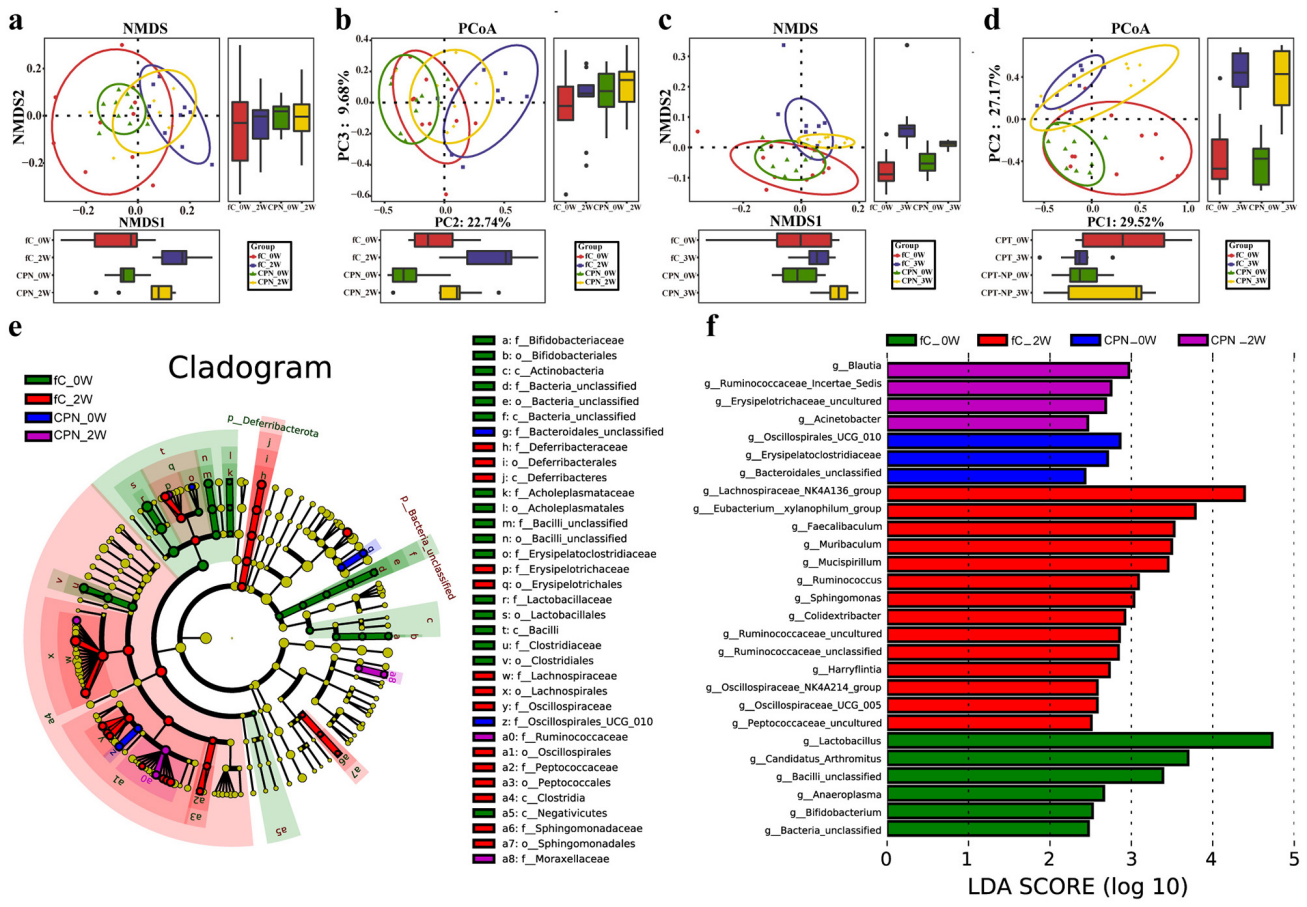
Effects of fC and CPNs on gut microecology of mice at week 2 and week 3. **(a, b)** After 2 weeks of administration, the changes of the gut microbiome in CPNs were weaker than those in fC. At week 3, both NMDS **(c)** and PCoA **(d)** analysis showed greater changes in the fC group than in the CPNs. **(e)** A cladogram drawn by the LEfSe method shows the phylogenetic distribution of fecal microbiome in different groups. The circles radiating from the inside out represent the taxonomic level from the phylum to the genus. Each circle on a level represents a classification at that level, and the diameter of the circle represents its relative abundance. Microorganisms with no significant differences are yellow, and biomarkers with significant differences follow grouped colors. **(f)** LDA scoring plot showing statistically significant differences among the different groups of gut microbiomes. The higher the LDA score, the higher the importance of microbial biomarkers. Data are derived from longitudinal sampling of independent biological replicates (*n* = 10 mice per group). fC, free-camptothecin; CPNs, PHA nanoparticles loaded with CPT; NMDS, Non-metric multidimensional scale; PCoA, Principal coordinate analysis. LEfSe, Linear discriminant analysis effect size; LDA, Linear discriminant analysis; OTUs, Operational taxonomic units.

**Figure 7:**
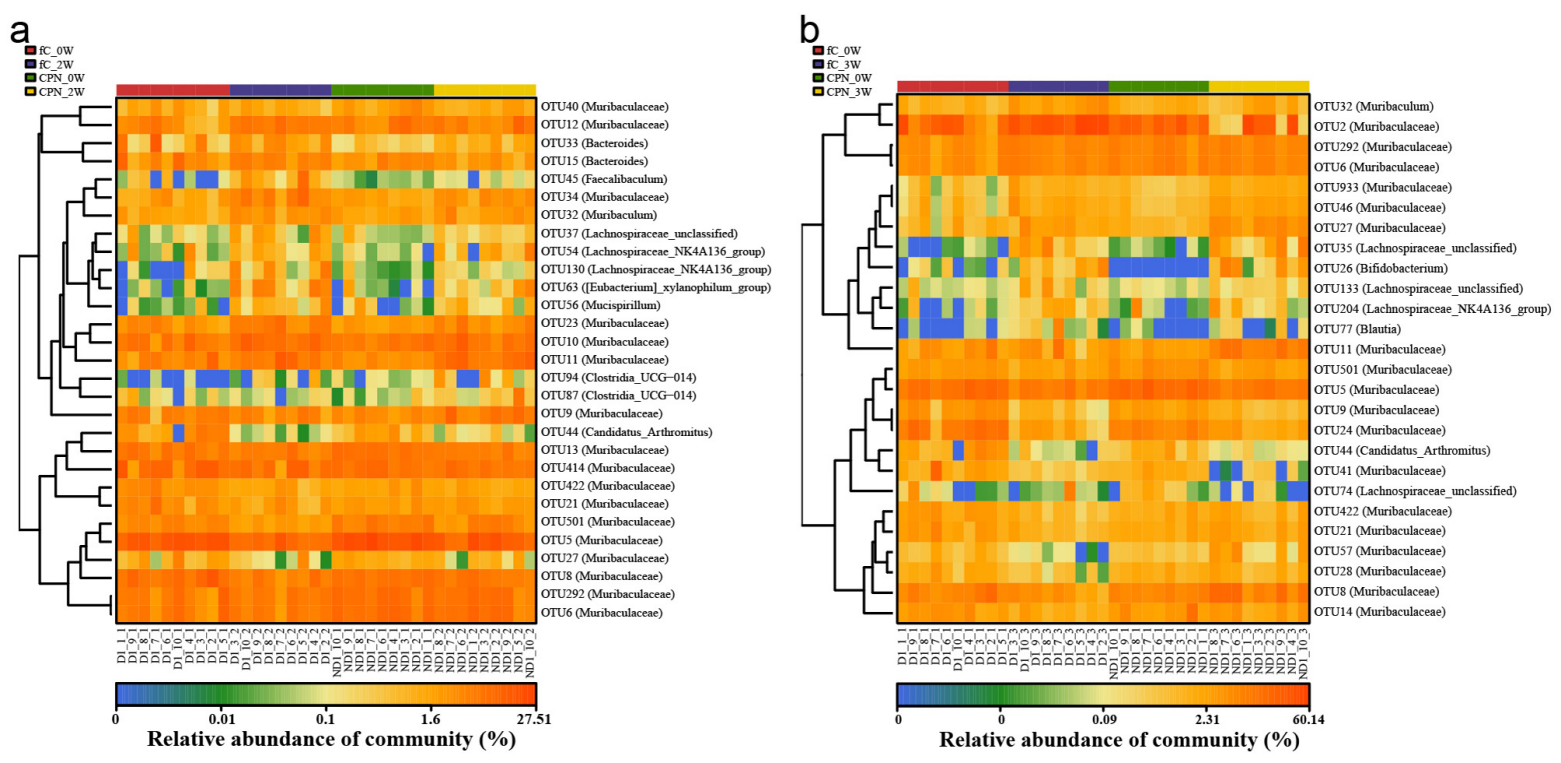
Heatmap of the relative abundance of OTUs differences between the fC group and the CPNs group at week 2 **(a)** and week 3 **(b)**. For each sample, the column on the right shows the relative abundance data for the different OTUs. Use the relative abundance of each OTU to plot the heatmap (blue, low abundance; red, high abundance). Analyses are based on longitudinal data from biological replicates (*n* = 10 per group). fC, free-camptothecin; CPNs, PHA nanoparticles loaded with CPT; OTUs, Operational taxonomic units.

Compared with the fC group, the gut microbiome in the CPNs group at week 2 and week 3 was closer to the baseline (Figures 6a–d), indicating that the effect of camptothecin on the gut microecology of mice after nanocoupling became smaller. In addition, the baseline data of the two groups could not be significantly distinguished, reflecting the reliability of the data. The heatmap (Figures 7a, b and Supplementary Datas S13, S14) showed the abundance of differential key OTUs between the four groups. Regardless of week 2 or week 3, the abundance of 5 OTUs in the CPNs group was significantly weaker than that in the fC group. Subsequently, we performed an average composition analysis and comparative analysis of gut microbes in the CPNs group at different levels. The results indicated that the dominant flora was highly similar to the fC group at both the phylum and genus levels. The abundance of *Deferribacterota* in the fC group changed more significantly than that in the CPNs group whether at both week 2 and week 3 (Supplementary Figures 3a, b and Supplementary Datas S9, S11). At the genus level, there were significant differences in 9 genera at week 2 (Supplementary Figure 3c and Supplementary Data S10), of which 8 genera had more prominent changes in abundance in the fC group than in the CPNs group. There were significant differences in 10 genera at week 3 (Supplementary Figure 3d and Supplementary Data S12), and the changes of *Muribaculum, Mucispirillum, Lachnospiraceae_NK4A136_group, Faecalibaculum*, and *Blautia* in the fC group were always higher than those in the CPNs group. We used LEfSe to select the maximum taxonomic differences. The cladogram showed significant changes in the gut microbiome of the fC group at week 2 (Figure 6e). At the genus level, LEfSe analysis and LDA scores observed that the changes of intestinal flora in the CPNs group were not as significant as fC at week 2 (LDA > 2.5, *P* < 0.05) (Figure 6f).

We analyzed the bacteria with significant differences between groups and found that CPT significantly decreased the abundance of probiotics such as *Akkermansia, Lactobacillus, Candidatus_Arthromitus*, and *Bacilli_unclassified* in the gut of mice, while the abundance of *Lachnospiraceae_NK4A136_group, Faecalibaculum, [Eubacterium]_xylanophilum_group* and other bacteria increased significantly. However, the change amplitude of these bacteria in the CPNs group was weaker than that in the fC group. The smaller effect of nanomaterials on the intestinal flora of mice can alleviate adverse drug reactions to some extent, and may also play a positive role in tumor treatment.

### 3.5 CPNs had a lower effect on the serum metabolomics of mice than that of fC

We used ultra-performance liquid chromatography-mass spectrometry (UPLC-MS) to analyze the serum metabolomics of mice. However, due to the difficulty in collecting venous blood from mice, serum from 5 mice at the baseline level of the control group was finally used as the overall baseline reference. Firstly, we used partial least squares discriminant analysis (PLS-DA) to reduce the dimensionality of the data at different time points in the fC group and screened the differential metabolites. The difference among the three groups was obvious (Supplementary Figure 4a), and the intercept of Q2 in the permutation test was less than 0, indicating that the fitting effect of the OPLS-DA (orthogonal partial least squares discriminant analysis) model was good (Supplementary Figure 4b). Subsequently, we screened out representative metabolites with significant differences (variable importance in projection (VIP) > 2, *P* < 0.05), and there were 5 representative metabolites whose expression increased or decreased after fC gavage, and gradually recovered after discontinuation (Supplementary Figure 4c and Supplementary Data S15).

**Figure 8:**
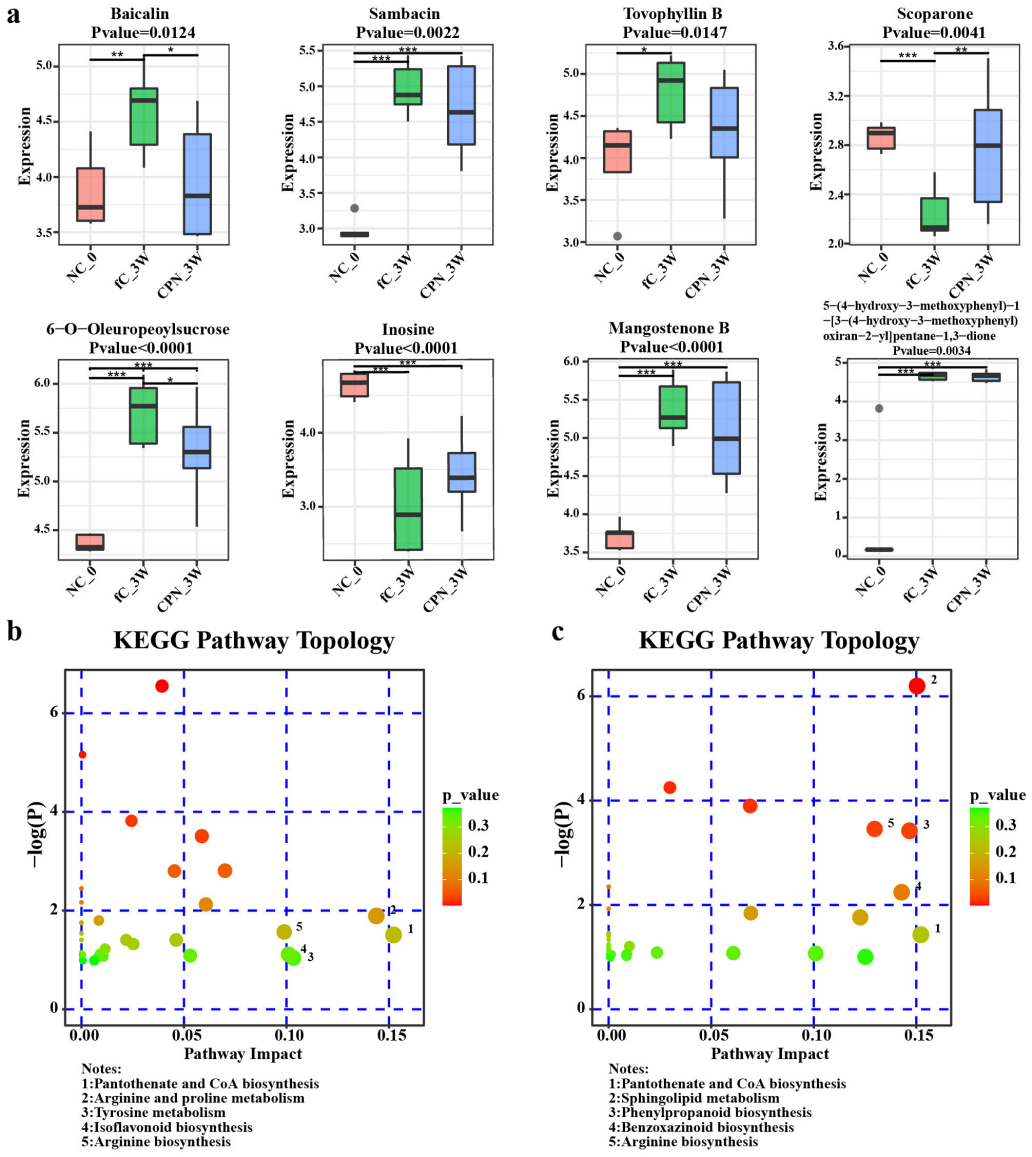
Significantly differential metabolites and topology in the fC group and CPNs group. **(a)** The abundance of six significantly differential metabolites decreased in the CPN group than in the fC group, while 2 metabolites increased in the CPN group. Topology of fC group and CPN group at week 2 **(b)** and week 3 **(c)**. Each bubble in the figure represented a KEGG Pathway, and the horizontal axis represented the relative importance of metabolites in the pathway; The longitudinal axis represented the enrichment significance of metabolites involved in pathways; The Bubble size representation was expressed in Impact Value; The larger the bubble, the greater the importance; The color represented the *p*-value of the pathway enrichment. Metabolomic data were obtained from longitudinal sampling of biological replicates (*n* = 10 mice per group). fC, free-camptothecin; CPNs, PHA nanoparticles loaded with CPT; KEGG, Kyoto Encyclopedia of Genes and Genomes. *P < 0.05, **P < 0.01, ***P < 0.001.

At week 2 and week 3, there were 16 different metabolites among the three groups (VIP > 2, *P* < 0.05). At week 2, we selected 11 representative metabolites (Supplementary Figure 5), and they were mainly concentrated in 10 pathways such as Cushing syndrome, bile secretion, and neuroactive ligand-receptor interaction. KEGG pathway enrichment analysis showed that pathways such as Arginine and proline metabolism were enriched with more differential metabolites at week 2 (Figure 8b and Supplementary Data S16). At week 3, we selected 8 representative differential metabolites (Figure 8a) and performed KEGG pathway enrichment analysis (Figure 8c and Supplementary Data S17). The pathways such as Sphingolipid Metabolism, Phenylpropanoid biosynthesis, and Arginine biosynthesis were enriched with more different metabolites at week 3.

Our study found that the increase of metabolites such as abscisic acid and the decrease of metabolites such as cortisol were not as significant in the CPNs group as they were in the fC group, meaning that the nanomaterials also had less metabolic impact on the mice. The differential metabolite enrichment pathways overlapped in the fC group and CPNs group to some extent, which included mainly sphingolipid metabolism, bile secretion, purine metabolism, aldosterone-regulated sodium reabsorption, phenylalanine, tyrosine and tryptophan biosynthesis, beta-alanine metabolism, ABC transporters, and other pathways. Metabolic changes in cancer therapy are key determinants of therapeutic toxicity and response. Our study also found differences between fC and CPNs in phenylalanine metabolism and essential amino acid metabolism, which may be related to the safety and efficacy of CPNs and fC in subsequent tumor therapy.

### 3.6 Interaction network among gut microbiota, plasma metabolites, and KEGG pathways

Our correlation analysis identified significant interactions among 5 gut microbiota genera, 27 plasma metabolites, and 70 KEGG pathways, revealing a complex regulatory network. *Faecalibaculum* emerged as a central hub genus, displaying distinct metabolic modulation patterns. It exhibited strong positive correlations with N2-(Dimethylamino)ethanesulfonic acid (a membrane-stabilizing compound; R = 0.80, *P* < 0.001) and LysoPE 22:6 (an anti-inflammatory lipid mediator; R = 0.67, *P* = 0.009). Conversely, it was negatively correlated with stress- and inflammation-associated metabolites, including 5-Oxo-L-proline (Pyroglutamic acid), LysoPC 20:3, 2-Pentylthiophene, Cortisol, and Aldosterone (R = −0.70 to −0.85, *P* < 0.01). *Bacteroides* and *Oscillospiraceae_UCG-005* showed similar regulatory trends, with positively associations to LysoPE 22:6 (R = 0.71, *P* = 0.005; R = 0.77, *P* = 0.001), and negatively correlations with Cortisol (R = −0.55, *P* = 0.041) and Aldosterone (R = −0.53, *P* = 0.049). These findings suggest a coordinated microbial strategy to attenuate host stress responses by modulating lipid metabolism and endocrine signaling.

KEGG pathway analysis further elucidated the functional mechanisms underlying these interactions. The identified microbiota genera were predicted to enhance key metabolic enzymes including O-Acetylhomoserine thiol-lyase (MetY) (EC:2.5.1.49), pyruvate formate-lyase activating enzyme (PflA/PflC/PflE) (EC:1.97.1.4), two-component system sensor histidine kinase KdpD (EC:2.7.13.3), and oxaloacetate decarboxylase subunit β (OadB) (EC:4.1.1.3), while simultaneously suppressing pathways involving uncharacterized protein (Hypothetical protein), acetate kinase (AckA) (EC:2.7.2.1), ribonuclease HI (RnhA) (EC:3.1.26.4), amino acid transporters (AAT family), and DNA segregation ATPases (FtsK/SpoIIIE). Collectively, these modulations likely influence host energy metabolism, amino acid homeostasis, ion balance, and genomic stability, thereby maintaining metabolic equilibrium.

Of particular interest, *Peptococcaceae_uncultured* demonstrated a positive correlation with Dihydro-3-coumaric acid (R = 0.67, *P* = 0.009) but negative associations with Phenylethyl glucuronide, 4-Ethylphenyl sulfate, and 21-Deoxycortisol (R = −0.76 to −0.82, *P* < 0.01). Pathway annotation linked these metabolites to bacterial cell wall integrity [MurE ligase (EC:6.3.2.13)], protein dynamics (SecY/SecA translocases, ClpC protease), and ribosomal function [RpmG, Gmk (EC:2.7.4.8)], suggesting that this genus may restrict pathogenic bacteria by targeting cell wall synthesis, protein turnover, and stress adaptation. These results systematically demonstrate a dual regulatory axis through which gut microbiota influence host physiology: (1) Immunomodulation via lipid mediators (e.g., LysoPE 22:6); (2) Metabolic reprogramming via energy metabolism optimization and stress hormone suppression. This integrative analysis provides novel mechanistic insights into microbiota-host crosstalk and highlights potential therapeutic strategies for microbiota-based interventions in metabolic disorders.

## 4 Discussion

The treatment of advanced tumors mainly relies on chemotherapy, but often faces the challenge of ineffective treatment due to adverse reactions and increased drug resistance. Camptothecin, as a natural plant-derived alkaloid, has shown potent anti-tumor activity by targeting intracellular topoisomerase I, but its practical therapeutic prospects were limited by a variety of factors. Currently, nanotechnology-based delivery systems have received great attention as a strategy to overcome challenges related to bioavailability, solubility, distribution, toxicity, and so on (Lagoa et al., 2020; Kashyap et al., 2021; Davatgaran-Taghipour et al., 2017). Novel strategies involving CPT pharmacological and low doses combined with nanoparticles may bring different anti-tumor effects. In this context, biodegradable nanoparticles have become the preferred drug carrier due to their effectiveness in encapsulating drugs, ability to control drug release, and low cytotoxicity. Biodegradable polymers have been applied to drug delivery systems in many previous studies (Sung and Kim, 2020). Both natural and synthetic polymers can improve drug pharmacokinetics to a certain extent and provide ideal physicochemical properties for the controlled release of therapeutic drugs. Among them, PHAs, a class of non-cytotoxic polyester synthesized by a variety of bacteria (Zhao et al., 2021a; Xiang et al., 2022; Wei and Zhang, 2022; Wei et al., 2011), have the advantages of good biocompatibility, high biodegradability, and high loading efficiency, making them suitable for various biomedical and pharmaceutical applications (Manavitehrani et al., 2016); they have been widely applied in the fields of drug release (Hu et al., 2020; Zhao et al., 2021b; Chen et al., 2020b; Ding et al., 2022), tissue repair (Wei et al., 2018b,a), and biomimetic vaccines (Peng et al., 2021). Previous studies have also reported PHA-based strategies for encapsulating therapeutic drugs (Zhang et al., 2021b) and cells (González et al., 2020), implying the potential of PHAs as an alternative to other pharmaceutical materials and other biopolymers. PHBVHHx, a new member of microbial polyhydroxyalkanoate tri-polyester, has been reported to be successfully used in tissue engineering (Hu et al., 2009; Liang et al., 2008; Ji et al., 2008). For the first time, we assembled CPT with PHBVHHx nanoparticles to form composite drugs and conducted comprehensive *in vitro* and *in vivo* evaluations.

*In vitro*, CPNs showed good CPT loading capacity and could achieve cell-killing effects via carrier degradation and sustained CPT release, making them suitable for oral administration. In the cell proliferation inhibition model, CPNs showed similar inhibitory effects on cell activity as continuous administration of CPT on day 7. And the pPNs group had no inhibitory effect on HT-29 cells, confirming the safety of the vector. *In vivo* experiments, we chose not only histological and clinical assays but also microbiome and metabolomic analyses. Studies have shown that CPT-11 was metabolized by hepatic carboxylesterase to its toxic form SN-38, which was mainly distributed in the liver, kidney, spleen, lungs, and heart (Yao et al., 2017). However, there are a large number of microorganisms in the intestine, which can regulate the tumor microenvironment and participate in drug metabolism. SN-38 can be further transformed into SN-38G through UDP glucuronidase 1A1 and then excreted into the ileum with bile. There, β-glucuronidase produced by intestinal microbiota is transformed into SN-38, which is toxic to intestinal epithelial cells (Yao et al., 2017; Akbarali et al., 2022), eventually causing intestinal side effects such as diarrhea. The study of intestinal microbiota may be of great significance for the treatment and prognosis of tumors. Additionally, non-targeted metabolomics studies have identified differential metabolites in CPT-11 treated tissues, revealing disruptions in the Krebs cycle, amino acid, purine, and bile acid metabolism (Yao et al., 2017).

Therefore, we also focused on changes in gut microbiome and serum metabolism in addition to routine histological and clinical measures. The changes in body weight, histological of liver/kidney/small intestine/colon, intestinal microbiota, and serum metabolism in the CPNs group were all less pronounced than those in the fC group, demonstrating improved safety of the nanomaterial. In addition, we found that fC significantly reduced the abundance of probiotics such as *Akkermansia* and *Lactobacillus*, while the abundance of bacteria such as *Lachnospiraceae_NK4A136_group* and *Faecalibaculum* was significantly increased in the mouse gut. However, the changes in the above bacteria in the CPNs group were weaker. Studies have shown that *Akkermansia* and *Lactobacillus* can maintain flora balance and improve immunity, and are important core and beneficial flora in human and animal guts (Zhang et al., 2021a; Dempsey and Corr, 2022). The protective effect of CPNs on gut microbiota may originate from a dual mechanism. The primary mechanism involves the direct physical barrier provided by nano-encapsulation, which effectively prevents extensive contact between the drug and both the intestinal epithelium and commensal microorganisms, thereby reducing initial drug-induced damage at the source. Secondly, the inherent biodegradability of PHA may contribute to indirect microecological regulation. Studies have demonstrated that certain PHA components can be degraded by specific gut bacteria into metabolites such as short-chain fatty acids (e.g., butyrate) (Zha et al., 2025; Liu et al., 2024) which help maintain microbial balance, promote the growth of beneficial bacteria, and provide energy for the host. Concurrently, the large specific surface area of the nanocarriers may create favorable conditions for microbial adhesion, while the degradation process could subtly modulate the local microenvironment (e.g., *PH*). The synergistic effects of these mechanisms likely establish a relatively stable micro-niche for beneficial bacterial communities under chemotherapy-induced stress, thereby supporting their survival and colonization.

Similar changes also appeared in the detection of serum metabolites. Compared to the fC group, the CPNs group showed attenuated changes in metabolites such as abscisic acid (increase) and cortisol (decrease). Moreover, the differential metabolite enrichment pathways of the two groups overlapped to some extent, mainly including sphingolipid metabolism, bile secretion, purine metabolism, and the phenylalanine, tyrosine and tryptophan biosynthesis pathway. Metabolic changes in tumor therapy are key factors in determining toxicity and response to treatment. Metabolic disorders, including dysglycolysis, glutamine decomposition, one-carbon metabolism, and fatty acid synthesis, promoted the occurrence and development of colorectal cancer. And differences in phenylalanine metabolism and essential amino acid metabolism may influence the therapeutic response to neoadjuvant chemoradiotherapy (nCRT) (Wang et al., 2022). The differences between fC and CPNs in phenylalanine metabolism and essential amino acid metabolism found in this study may help explain the safety and efficacy differences in subsequent tumor therapy. From a certain point of view, the lower impact of nanomedicine on gut microbes and serum metabolites may also have some significance in alleviating drug side effects.

In conclusion, we successfully developed a CPT-PHBVHHx nanoparticle complex (CPNs) and characterized its properties. *In vitro* experiments, the drug loading efficiency, slow-release properties, and inhibitory effect was verified. *In vivo* experiments proved that the nano-drug has better biosafety and lower toxicity in mice, and the damage to liver, kidney, and intestine was significantly lower than that caused by fC. The nanoparticles can spread to the small intestine and colon tissue in the villous space, and further spread into the blood for drug release. Critically, CPNs caused markedly milder perturbations to the gut microbiome and serum metabolome than free CPT, suggesting reduced microbiota- and metabolism-mediated side effects. It is worth noting that this novel nano-drug improves the pharmacokinetics and stability of CPT, and its oral administration is convenient, economical, and ideal for long-term drug administration in tumor patients.

## 5 Limitations of the study

In this study, we synthesized a novel composite nanomedicine, CPNs, and preliminarily confirmed its safety advantages over free CPT in both *in vitro* and *in vivo* settings. However, a limitation of this work is that the antitumor efficacy has not been evaluated in tumor-bearing models, nor have the pharmacokinetic properties been systematically analyzed. As a result, the potential advantages of CPNs in terms of efficacy and pharmacokinetics compared to clinical standard camptothecin formulations (such as irinotecan) remain to be directly confirmed. These aspects will be key focuses of future investigations.

## Data Availability

The original contributions presented in the study are included in the article/Supplementary material, further inquiries can be directed to the corresponding authors.
