# Supplementary material for: Camptothecin-PHA nanoparticles attenuate drug-induced gut microbiome dysbiosis and metabolic toxicity
Source: Front Microbiol. 2026 Jan 28;16:1617468. doi: 10.3389/fmicb.2025.1617468 (PMC12893722; doi:10.3389/fmicb.2025.1617468)
Supplement: Supplementary file 1 [file Data_Sheet_1.pdf]

Supplementary Materials

This PDF file includes:

Figure S1 to S5
Data S1 to S17

Data S1. Alpha_diversity of pPNs group at different time points
Data S2. Alpha_diversity of pPNs and NC at week 2
Data S3. Data S3. Alpha_diversity of pPNs and NC at week 3
Data S4. Heatmap of fC group at different time points
Data S5. Average abundance of gut microbiome at the phylum level in fC group
Data S6. Average abundance of gut microbiome at the genus level in fC group
Data S7. Comparison of gut microbiome at the phylum level in fC group
Data S8. Comparison of gut microbiome at the genus level in fC group
Data S9. Comparison of gut microbiome at the phylum level between fC and CPNs at week 2
Data S10. Comparison of gut microbiome at the genus level between fC and CPNs at week 2
Data S11. Comparison of gut microbiome at the phylum level between fC and CPNs at week 3
Data S12. Comparison of gut microbiome at the genus level between fC and CPNs at week 3
Data S13. Heatmap of fC group and CPNs group at week 2
Data S14. Heatmap of fC group and CPNs group at week 3
Data S15. KEGG pathway of different metabolites in fC group at different time points
Data S16. KEGG pathway of different metabolites between fC group and CPNs group at week 2
Data S17. KEGG pathway of different metabolites between fC group and CPNs group at week 3

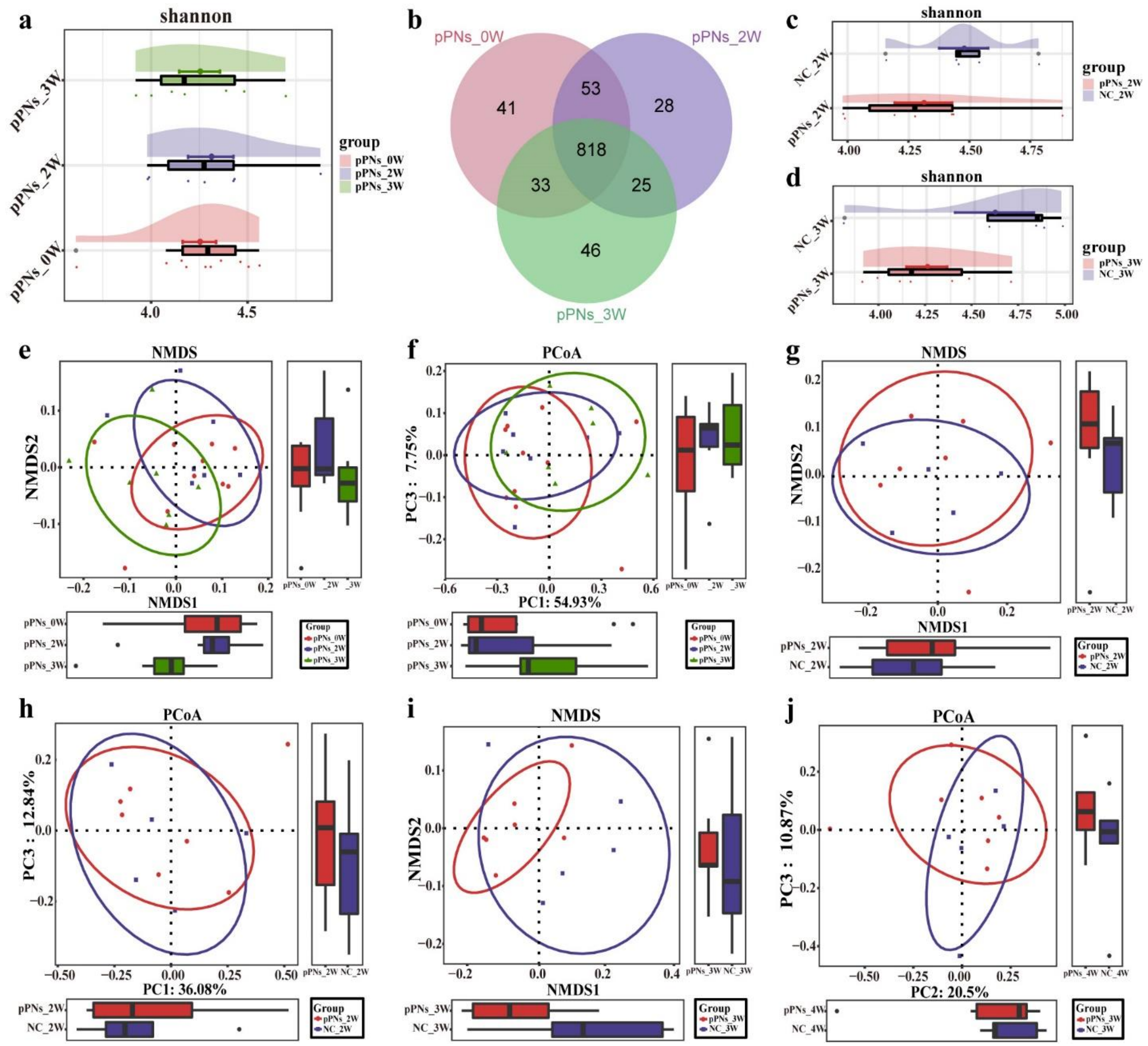

Supplementary Fig. 1. pPNs had no significant effect on the gut microecology of mice. **(a)** Shannon index of baseline, week 2 and week 3 in the pPNs group; **(b)** The Venn plot indicated that 818 of the total richness of the 1044 OTUs were shared between the three groups; **(c)** and **(d)** were Shannon graphs for week 2 and week 3 between NC and pPNs group, respectively; **(e)** NMDS analysis revealed gut microbiome distribution and differences in pPNs group at three different points in time. **(f)** PCoA analysis showed microbial similarities and differences in the pPNs group at three different points in time; **(g)** and **(h)** represented NMDS analysis and PCoA analysis at week 2 of pPNs group and NC group, respectively; **(i)** and **(j)** represented NMDS analysis and PCoA analysis at week 3 in the pPNs group and NC group, respectively. NC: Normal Control; pPNs: pure PHA nanoparticles; OTUs: Operational taxonomy units; NMDS: Non-metric multidimensional scale; PCoA: Principal Coordinate Analysis.

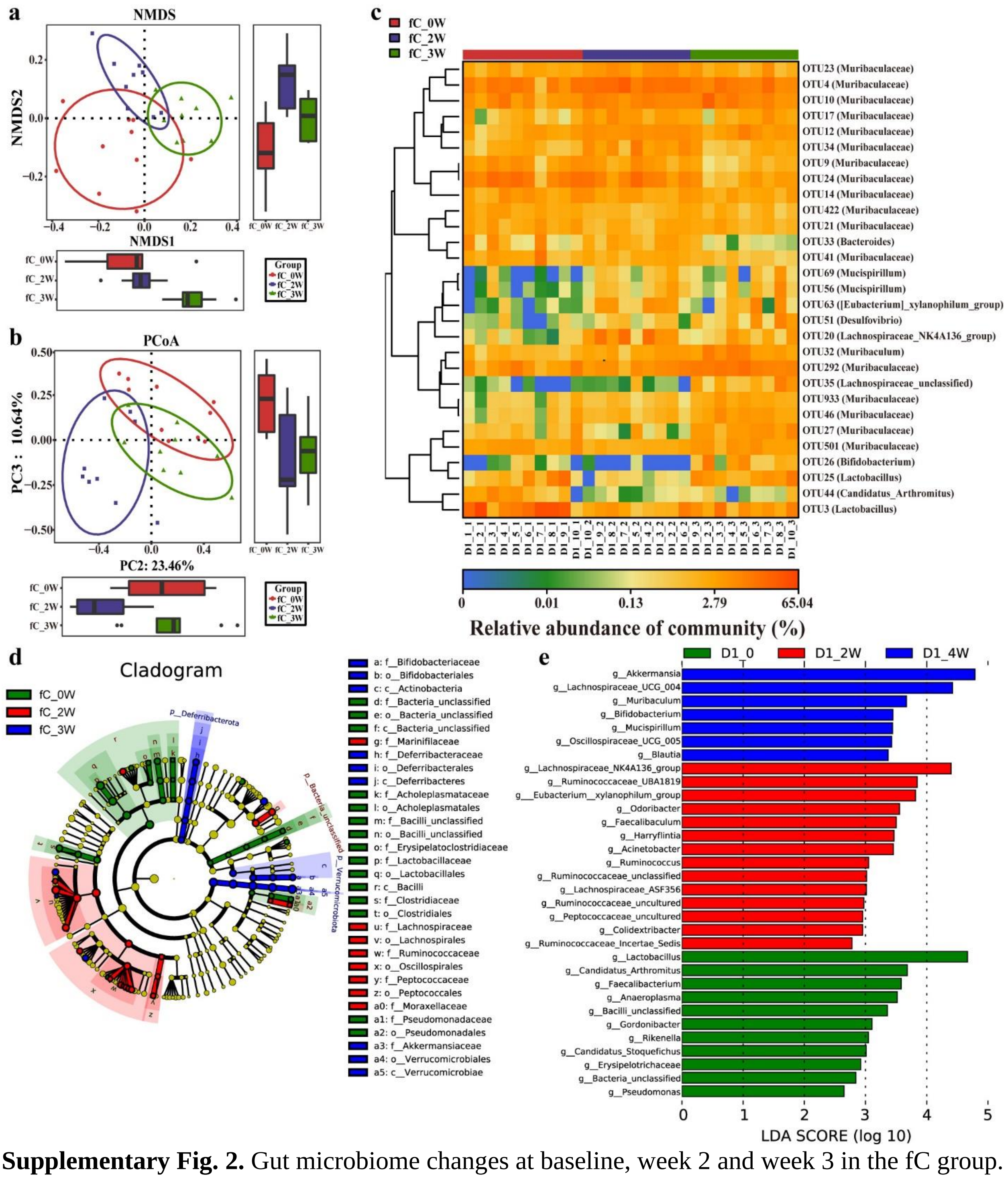

Supplementary Fig. 2. Gut microbiome changes at baseline, week 2 and week 3 in the fC group. **(a)** NMDS analysis was used to analyze the distribution and differences of intestinal microbes at different time points in the fC group. **(b)** The degree of similarity and differences in gut microbes at different time points in the fC group was studied by PCoA. **(c)** The heatmap showed the abundance of different OTUs at different time points after oral administration of fC. **(d)** Cladogram drawn by the LEfSe method showing the phylogenetic distribution of fecal microbiome in three groups. The circles radiating from the inside out represent the taxonomic level from the phylum to the genus. Each circle on a level represents a classification at that level, and the diameter of the

circle represents its relative abundance. Microorganisms with no significant differences are yellow, and biomarkers with significant differences follow grouped colors. Default LDA > 2.5 and $P < 0.05$ indicate different species and higher abundance in one group than the other. **(e)** LDA scoring plot showing statistically significant differences among the three groups of gut microbiome. The higher the LDA score, the higher the importance of microbial biomarkers. The default LDA score is greater than 3 and the p-value is less than 0.05, which is considered a different species. fC: free-camptothecin; NMDS: Non-metric Multidimensional Scale; PCoA: Principal Coordinate Analysis; OTUs: Operational taxonomy units. LEfSe: Linear Discriminant Analysis Effect Size; LDA: Linear Discriminant Analysis.

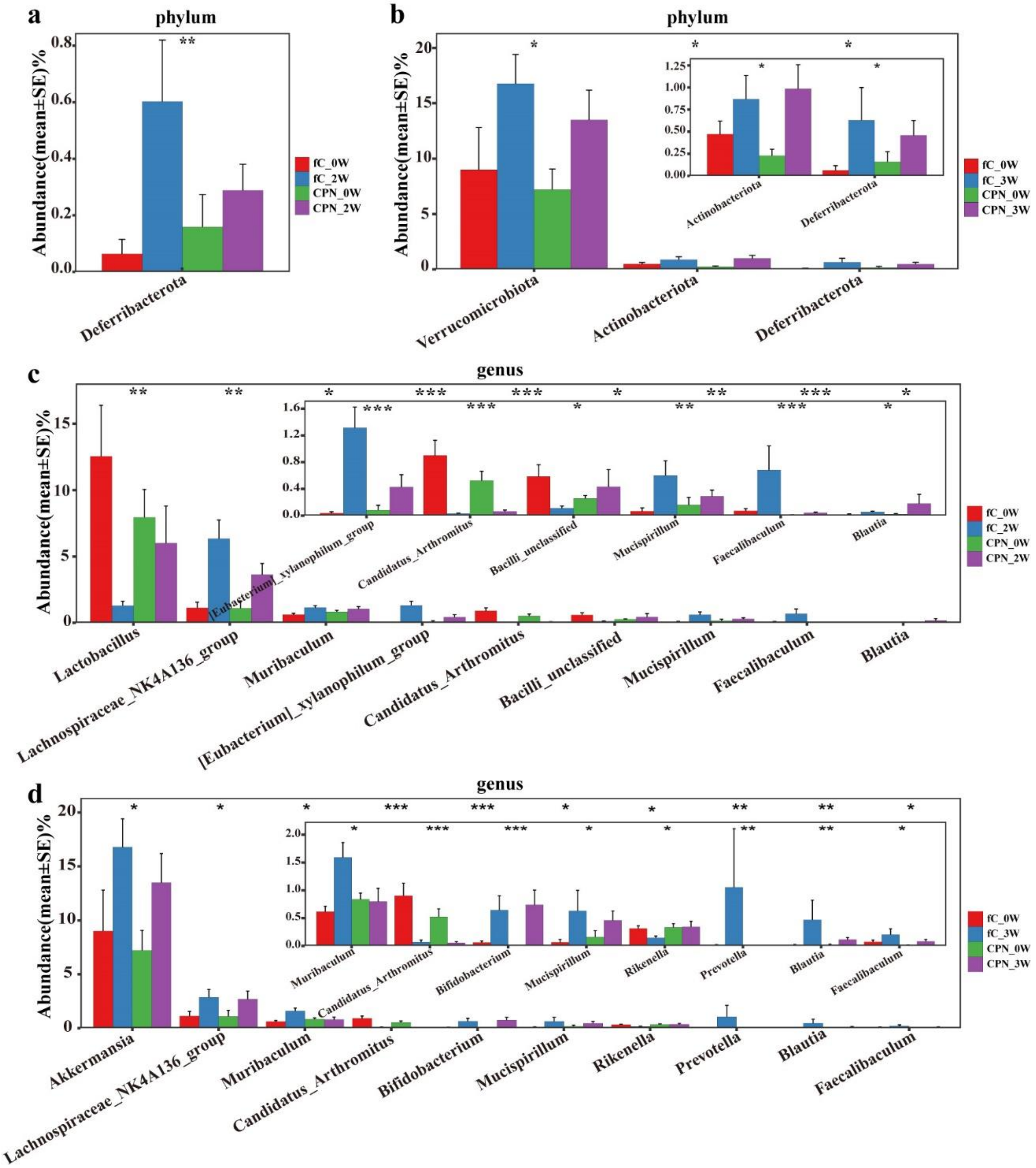

Supplementary Fig. 3. Abundance comparison between the fC group and the CPNs group at different times. At the phylum level, the fC group was compared with the CPNs group for differential microorganisms at week 2 **(a)** and week 3 **(b)**. At the genus level, the fC group was compared with the CPN group for differential microorganisms at week 2 **(c)** and week 3 **(d)**. fC: free-camptothecin; CPNs: PHA nanoparticles loaded with CPT.

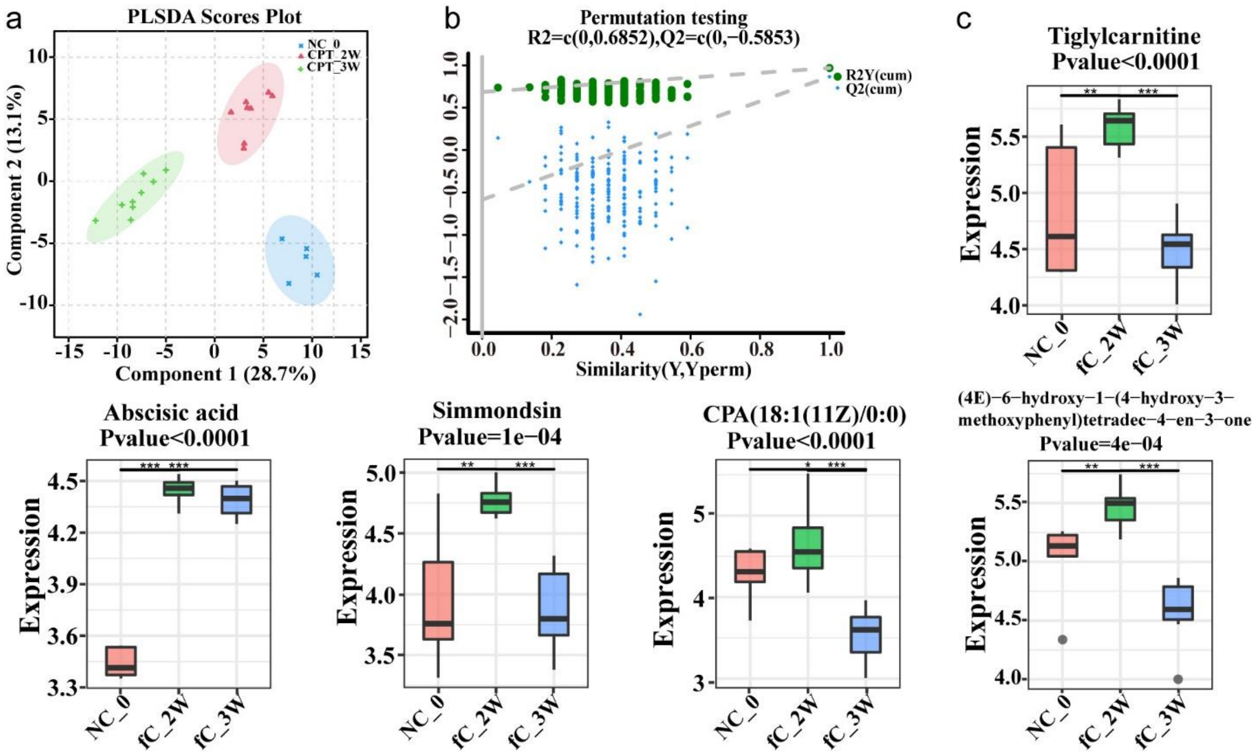

Supplementary Fig. 4. PLS-DA scores plot, permutation test and significantly differential metabolites at different time points in the fC group. **(a)** PLS-DA scores plot showed that the separation between the three groups of samples was large and the classification effect was remarkable. The interpretation of Componet 1 was 28.7%, and the interpretation of the second principal component of Componet 2 was 13.1%. **(b)** All blue Q2 values were lower than the original point and the regression line of Q2 point intersected with the vertical axis below zero, indicated the model quality was better. **(c)** The expression of the 5 significantly differential metabolites increased at 2 weeks and resumed after discontinuation. PLS-DA: Partial Least-Squares Discriminant Analysis; fC: free-camptothecin.

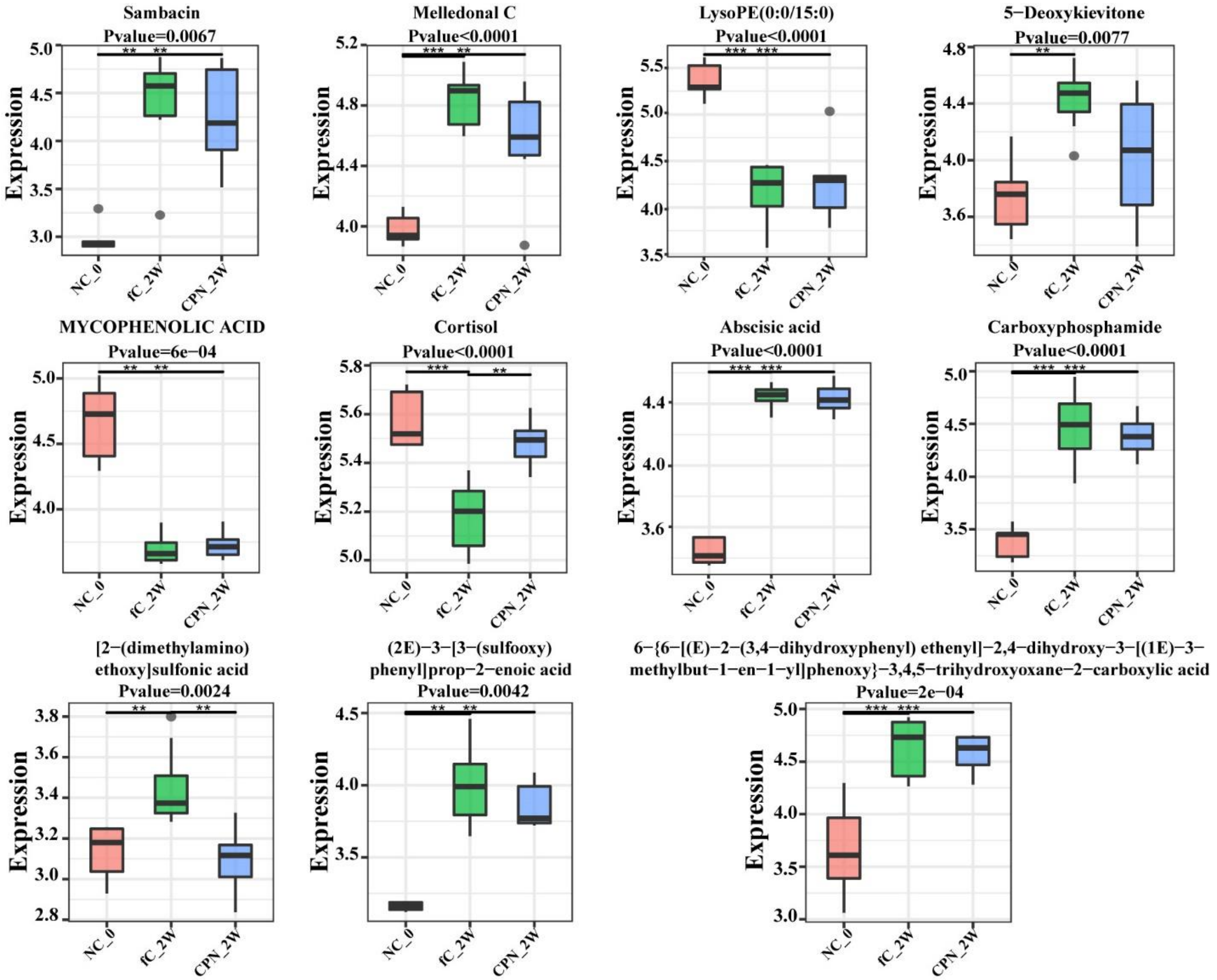

Supplementary Fig. 5. Significantly differential metabolites in fC group and CPN group at week 2. The expression of the 8 significantly differential metabolites increased in CPN group than fC group, while 3 metabolites decreased in CPN group. fC: free-camptothecin; CPN: PHA nanoparticles loaded with CPT.

Data S1. Alpha_diversity of pPNs group at different time points			
Alpha_diversity	ace	chao	shannon
pPNs_0-median	671.9219(651.926,695.5696)	686.5307(652.72,710.9765)	4.2941(4.1632,4.4365)
pPNs_0-mean	676.2220309	687.6748176	4.2499562
pPNs_0-se	14.51794415	15.6244141	0.086481925
pPNs_2W-median	692.2448(649.6678,720.9583)	700.9041(650.4543,745.0751)	4.2744(4.0893,4.4272)
pPNs_2W-mean	687.1838531	694.7661267	4.309148571
pPNs_2W-se	20.5412111	23.82922665	0.117064493
pPNs_3W-median	686.8599(663.4149,714.2728)	717.625(671.5261,722.6951)	4.1717(4.0524,4.4328)
pPNs_3W-mean	698.8611051	711.6756657	4.251095286
pPNs_3W-se	21.69448787	22.05997794	0.105623106
p-value	0.714112843	0.696879437	0.939480166
z-score	-0.366338046	-0.389536335	-0.075923242
Sig_mark			
Alpha_diversity	simpson	observed_otus	
pPNs_0-median	0.0331(0.0294,0.0468)	577.5(547.75,598.25)	
pPNs_0-mean	0.0454189	578.4	
pPNs_0-se	0.009430475	14.12027699	
pPNs_2W-median	0.0309(0.028,0.0385)	577(545.5,636.5)	
pPNs_2W-mean	0.031590429	590	
pPNs_2W-se	0.003511501	24.24478343	
pPNs_3W-median	0.057(0.0337,0.0595)	584(577,617)	
pPNs_3W-mean	0.047449286	611.1428571	
pPNs_3W-se	0.006200805	25.95364785	
p-value	0.283168179	0.608000459	
z-score	-1.073229453	-0.512929754	
Sig_mark			

Data S2. Alpha_diversity of pPNs and NC at week 2			
Alpha_diversity	ace	chao	shannon
pPNs_2W-median	692.2448(649.6678,720.9583)	700.9041(650.4543,745.0751)	4.2744(4.0893,4.4272)
pPNs_2W-mean	687.1838531	694.7661267	4.309148571
pPNs_2W-se	20.5412111	23.82922665	0.117064493
NC_2W-median	703.1216(697.288,707.3664)	718.3944(700.4,733.0476)	4.4529(4.4429,4.5363)
NC_2W-mean	692.7678028	706.7642304	4.4729274
NC_2W-se	26.86861871	25.98992079	0.09997411
p-value	0.755050505	0.755050505	0.202020202
z-score	-0.311986866	-0.311986866	-1.275817041
Sig_mark			
Alpha_diversity	simpson	observed_otus	
pPNs_2W-median	0.0309(0.028,0.0385)	577(545.5,636.5)	
pPNs_2W-mean	0.031590429	590	
pPNs_2W-se	0.003511501	24.24478343	
NC_2W-median	0.0256(0.0226,0.0288)	602(591,610)	
NC_2W-mean	0.0267996	592.4	
NC_2W-se	0.00273077	22.19143979	
p-value	0.267676768	0.935169874	
z-score	-1.10842858	-0.081342125	
Sig_mark			

Data S3. Alpha_diversity of pPNs and NC at week 3			
Alpha_diversity	ace	chao	shannon
pPNs_3W-median	686.8599(663.4149,714.2728)	717.625(671.5261,722.6951)	4.1717(4.0524,4.4328)
pPNs_3W-mean	698.8611051	711.6756657	4.251095286
pPNs_3W-se	21.69448787	22.05997794	0.105623106
NC_3W-median	784.3036(779.7787,803.2318)	795.5581(789.1282,799.5676)	4.8265(4.5675,4.8511)
NC_3W-mean	774.2682684	780.3381268	4.6039006
NC_3W-se	20.95840431	19.12810346	0.20522553
p-value	0.073232323	0.148989899	0.148989899
z-score	-1.791380081	-1.443108537	-1.443108537
Sig_mark			
Alpha_diversity	simpson	observed_otus	
pPNs_3W-median	0.057(0.0337,0.0595)	584(577,617)	
pPNs_3W-mean	0.047449286	611.1428571	
pPNs_3W-se	0.006200805	25.95364785	
NC_3W-median	0.0182(0.0164,0.0323)	688(642,730)	
NC_3W-mean	0.0285704	676.4	
NC_3W-se	0.008442057	28.74299915	
p-value	0.073232323	0.165976487	
z-score	-1.791380081	-1.385248527	
Sig_mark			

Data S4. Heatmap of fC group at different time points					
	D1_1_1	D1_2_1	D1_3_1	D1_4_1	D1_5_1
OTU3 (Lactobacillus)	0.206693	0.650434	0.015886	0.080133	0.104109
OTU44 (Candidatus_Arthromitus)	0.030695	0.027619	0.080981	0.042374	0.052091
OTU25 (Lactobacillus)	0.002148	0.009269	0.011685	0.021954	0.006993
OTU26 (Bifidobacterium)	0	0	0.00011	0.000157	0.004569
OTU501 (Muribaculaceae)	0.029164	0.027127	0.040023	0.042604	0.045757
OTU27 (Muribaculaceae)	0.005785	0.012035	0.008115	0.006598	0.019192
OTU46 (Muribaculaceae)	0.00206	0.000225	0.005179	0.007917	0.005891
OTU933 (Muribaculaceae)	0.001371	9.84E-05	0.00497	0.005505	0.004042
OTU35 (Lachnospiraceae_unclassified)	0.000516	7.23E-05	0.000513	0.001006	0
OTU292 (Muribaculaceae)	0.059044	0.010803	0.038205	0.063181	0.122729
OTU32 (Muribaculum)	0.029948	0.003146	0.017953	0.023236	0.01228
OTU20 (Lachnospiraceae_NK4A136_group)	0.002997	0.00045	0.00971	0.000907	0.006806
OTU51 (Desulfovibrio)	0.000539	0.000432	0.00033	0.012008	0.00027
OTU63 ([Eubacterium]_xylanophilum_group)	0	0.000175	7.70E-05	0.005616	0.000284
OTU56 (Mucispirillum)	0	5.83E-05	0.011315	0.00033	0
OTU69 (Mucispirillum)	0	2.81E-05	0.007906	0.000186	0
OTU41 (Muribaculaceae)	0.030203	0.014345	0.040507	0.03117	0.026747
OTU33 (Bacteroides)	0.074966	0.001926	0.067925	0.016074	0.001896
OTU21 (Muribaculaceae)	0.036739	0.019116	0.046951	0.009488	0.019306
OTU422 (Muribaculaceae)	0.033569	0.018198	0.038634	0.042324	0.019057
OTU14 (Muribaculaceae)	0.034186	0.011283	0.018925	0.066335	0.040652
OTU24 (Muribaculaceae)	0.091897	0.069096	0.187122	0.135104	0.178873
OTU9 (Muribaculaceae)	0.0427	0.028965	0.069508	0.042279	0.065174
OTU34 (Muribaculaceae)	0.010979	0.002971	0.01957	0.022218	0.028626
OTU12 (Muribaculaceae)	0.032396	0.002646	0.008834	0.012313	0.021903
OTU17 (Muribaculaceae)	0.0435	0.000167	0.00364	0.011447	0.019822
OTU10 (Muribaculaceae)	0.061747	0.027645	0.074431	0.14319	0.037369
OTU4 (Muribaculaceae)	0.1086	0.051724	0.138536	0.103938	0.14391
OTU23 (Muribaculaceae)	0.027558	0.009949	0.032458	0.05041	0.011653
	D1_6_1	D1_7_1	D1_8_1	D1_9_1	D1_10_1
OTU3 (Lactobacillus)	0.168401	0.470561	0.427154	0.331095	0.005558
OTU44 (Candidatus_Arthromitus)	0.015107	0.00646	0.00394	0.017792	0
OTU25 (Lactobacillus)	0.06835	0.011546	0.086651	0.175923	0.002231
OTU26 (Bifidobacterium)	0.007654	6.74E-05	0.0017	0.000705	0
OTU501 (Muribaculaceae)	0.044812	0.016849	0.021429	0.023197	0.064365
OTU27 (Muribaculaceae)	0.00797	0.000232	0.01324	0.006269	0.060704
OTU46 (Muribaculaceae)	0.006228	0.000265	0.015844	0.004816	0.038656
OTU933 (Muribaculaceae)	0.004861	0.000122	0.011356	0.004222	0.023347
OTU35 (Lachnospiraceae_unclassified)	0.000115	0	0	0	0.000142
OTU292 (Muribaculaceae)	0.051966	0.012884	0.030767	0.034449	0.109659
OTU32 (Muribaculum)	0.014439	0.010749	0.010416	0.00498	0.017312

OTU20 (Lachnospiraceae_NK4A136_group)	0.00014	0.000192	3.10E-05	0.003989	0.003965
OTU51 (Desulfovibrio)	0	0	0.000277	0.004577	0.000877
OTU63 ([Eubacterium]_xylanophilum_group)	0	0.000162	0.00137	0.000138	0.000127
OTU56 (Mucispirillum)	0.00045	7.74E-05	4.92E-05	0.001342	0.000137
OTU69 (Mucispirillum)	0	5.99E-05	0	0.000451	0
OTU41 (Muribaculaceae)	0.02386	0.144377	0.0074	0.00624	0.016795
OTU33 (Bacteroides)	0.001275	0.084324	0.001751	0.000926	0.003195
OTU21 (Muribaculaceae)	0.026004	0.051781	0.017055	0.025902	0.044883
OTU422 (Muribaculaceae)	0.020826	0.033948	0.020296	0.022486	0.027682
OTU14 (Muribaculaceae)	0.058798	0.035833	0.020065	0.026289	0.064943
OTU24 (Muribaculaceae)	0.11598	0.006292	0.066561	0.113419	0.160642
OTU9 (Muribaculaceae)	0.05021	0.002519	0.02581	0.039543	0.060075
OTU34 (Muribaculaceae)	0.035685	0.016063	0.005928	0.005601	0.010689
OTU12 (Muribaculaceae)	0.038206	0.04303	0.029376	0.022205	0.041434
OTU17 (Muribaculaceae)	0.030871	0.003598	0.010361	0.001154	0.018042
OTU10 (Muribaculaceae)	0.074841	0.011049	0.038347	0.045293	0.051622
OTU4 (Muribaculaceae)	0.09983	0.006407	0.112964	0.050287	0.151945
OTU23 (Muribaculaceae)	0.033124	0.03055	0.019864	0.026711	0.020973
	D1_10_2	D1_9_2	D1_8_2	D1_7_2	D1_5_2
OTU3 (Lactobacillus)	0.0222	0.042948	0.056603	0.031535	0.005811
OTU44 (Candidatus_Arthromitus)	0.000226	0.00055	0.001814	6.97E-05	7.50E-05
OTU25 (Lactobacillus)	0	0.002713	0.005988	0.003214	0.003165
OTU26 (Bifidobacterium)	8.72E-05	0	0	0	0.00099
OTU501 (Muribaculaceae)	0.013977	0.025033	0.015851	0.014192	0.01727
OTU27 (Muribaculaceae)	0.003212	0.001279	0.001049	4.30E-05	0.041752
OTU46 (Muribaculaceae)	0.01231	0.010854	0.016592	0.023961	0.003837
OTU933 (Muribaculaceae)	0.00651	0.006353	0.015291	0.01057	0.002262
OTU35 (Lachnospiraceae_unclassified)	0.00011	0.000123	0.000177	4.10E-05	0.015773
OTU292 (Muribaculaceae)	0.03673	0.057974	0.056373	0.012905	0.062397
OTU32 (Muribaculum)	0.027912	0.020539	0.035723	0.004446	0.029492
OTU20 (Lachnospiraceae_NK4A136_group)	0.038271	0.145487	0.041088	0.167668	0.002379
OTU51 (Desulfovibrio)	0.000459	0.002316	0.008811	0.01302	0.000666
OTU63 ([Eubacterium]_xylanophilum_group)	0.00251	0.045007	0.045841	0.003532	0.003996
OTU56 (Mucispirillum)	0.001089	0.012548	0.010311	0.00396	0.000783
OTU69 (Mucispirillum)	0.000801	0.01011	0.007133	0.002374	0.000276
OTU41 (Muribaculaceae)	0.029402	0.01322	0.023482	0.008111	0.024908
OTU33 (Bacteroides)	0.023357	0.005451	0.036663	0.024186	0.016016
OTU21 (Muribaculaceae)	0.011728	0.008257	0.023436	0.024125	0.007428
OTU422 (Muribaculaceae)	0.014591	0.012548	0.015801	0.016793	0.005733
OTU14 (Muribaculaceae)	0.061941	0.02818	0.023056	0.041822	0.041134
OTU24 (Muribaculaceae)	0.152425	0.087182	0.031095	0.075056	0.212505
OTU9 (Muribaculaceae)	0.052894	0.038869	0.014167	0.02408	0.073059
OTU34 (Muribaculaceae)	0.063321	0.029501	0.061465	0.006858	0.116041
OTU12 (Muribaculaceae)	0.066035	0.053154	0.048244	0.048951	0.064479

OTU17 (Muribaculaceae)	0.053056	0.026587	0.075874	0.042662	0.044962
OTU10 (Muribaculaceae)	0.11806	0.087373	0.072971	0.046549	0.081987
OTU4 (Muribaculaceae)	0.141746	0.179489	0.188686	0.297141	0.091898
OTU23 (Muribaculaceae)	0.04504	0.046355	0.066416	0.052136	0.028925
	D1_4_2	D1_3_2	D1_2_2	D1_6_2	D1_9_3
OTU3 (Lactobacillus)	0.012107	0.016456	0.017404	0.109307	0.060217
OTU44 (Candidatus_Arthromitus)	0.000437	0.001174	0.001368	0.002586	0.00167
OTU25 (Lactobacillus)	0.000352	0.003011	0.002015	0.000979	0.067212
OTU26 (Bifidobacterium)	0	0	0	0	0.006918
OTU501 (Muribaculaceae)	0.021455	0.021566	0.020042	0.030262	0.044902
OTU27 (Muribaculaceae)	0.001052	0.005645	7.15E-05	0.000774	0.041664
OTU46 (Muribaculaceae)	0.010073	0.017467	0.024578	0.003647	0.019059
OTU933 (Muribaculaceae)	0.006613	0.012393	0.016929	0.001646	0.015188
OTU35 (Lachnospiraceae_unclassified)	5.39E-05	0.000399	0.000383	0	0.036571
OTU292 (Muribaculaceae)	0.047196	0.065175	0.071753	0.091468	0.113821
OTU32 (Muribaculum)	0.02501	0.041811	0.042645	0.03827	0.030654
OTU20 (Lachnospiraceae_NK4A136_group)	0.171769	0.053573	0.051617	0.002254	0.001335
OTU51 (Desulfovibrio)	0.00262	0.010633	0.010476	8.13E-05	0.014253
OTU63 ([Eubacterium]_xylanophilum_group)	0.059357	0.049762	0.042876	0.001256	0.000412
OTU56 (Mucispirillum)	0.005117	0.015213	0.040455	0.008344	0.002316
OTU69 (Mucispirillum)	0.005599	0.009915	0.030671	0.003435	0.001989
OTU41 (Muribaculaceae)	0.023479	0.026831	0.032598	0.022068	0.013799
OTU33 (Bacteroides)	0.006733	0.037939	0.039116	0.023824	0.015037
OTU21 (Muribaculaceae)	0.00539	0.025625	0.032166	0.031683	0.023597
OTU422 (Muribaculaceae)	0.00892	0.014109	0.01885	0.031475	0.024691
OTU14 (Muribaculaceae)	0.01981	0.022181	0.018713	0.047343	0.038258
OTU24 (Muribaculaceae)	0.099319	0.089098	0.035188	0.081095	0.060645
OTU9 (Muribaculaceae)	0.046293	0.03453	0.01211	0.03506	0.024756
OTU34 (Muribaculaceae)	0.030098	0.043935	0.033712	0.07254	0.043677
OTU12 (Muribaculaceae)	0.051936	0.059667	0.058551	0.062342	0.047401
OTU17 (Muribaculaceae)	0.014262	0.040283	0.039776	0.027184	0.024748
OTU10 (Muribaculaceae)	0.101857	0.075008	0.072432	0.071261	0.078688
OTU4 (Muribaculaceae)	0.163255	0.143946	0.166115	0.164366	0.096171
OTU23 (Muribaculaceae)	0.059836	0.062656	0.067389	0.03545	0.05035
	D1_2_3	D1_3_3	D1_4_3	D1_5_3	D1_6_3
OTU3 (Lactobacillus)	0.023432	0.157848	0.008963	0.060584	0.032521
OTU44 (Candidatus_Arthromitus)	0.005981	0.000566	0	0.000233	0.001026
OTU25 (Lactobacillus)	0.047348	0.029106	0.012488	0.012984	0.037419
OTU26 (Bifidobacterium)	0.116765	0.000239	0.035324	0.066905	0.004846
OTU501 (Muribaculaceae)	0.049272	0.014037	0.052101	0.038232	0.06152
OTU27 (Muribaculaceae)	0.041386	0.010749	0.038784	0.039325	0.052881
OTU46 (Muribaculaceae)	0.031155	0.069345	0.032101	0.046082	0.038264
OTU933 (Muribaculaceae)	0.021719	0.04748	0.020946	0.034767	0.024598

OTU35 (Lachnospiraceae_unclassified)	0.00244	0.028663	0.006191	0.006861	0.021645
OTU292 (Muribaculaceae)	0.108319	0.133402	0.117768	0.177206	0.100558
OTU32 (Muribaculum)	0.110942	0.057919	0.10582	0.090705	0.033761
OTU20 (Lachnospiraceae_NK4A136_group)	0.003144	0.006266	0.050939	0.023061	0.004953
OTU51 (Desulfovibrio)	0.002153	0.003828	0.019425	0.001692	0.057681
OTU63 ([Eubacterium]_xylanophilum_group)	0	0.00343	0.017798	0.011066	0.08932
OTU56 (Mucispirillum)	0.000402	0.001443	0.023007	0.000436	0.018693
OTU69 (Mucispirillum)	0.000167	0.000394	0.022464	0	0.011577
OTU41 (Muribaculaceae)	0.016767	0.006793	0.015018	0.004762	0.008277
OTU33 (Bacteroides)	0.000943	0.002244	8.77E-05	0.002576	0.000919
OTU21 (Muribaculaceae)	0.021351	0.038279	0.02003	0.009885	0.01575
OTU422 (Muribaculaceae)	0.008656	0.026596	0.016807	0.005105	0.015843
OTU14 (Muribaculaceae)	0.022911	0.015024	0.038916	0.020241	0.026329
OTU24 (Muribaculaceae)	0.00379	0.00974	0.007441	0.029225	0.048248
OTU9 (Muribaculaceae)	0.001474	0.004058	0.002609	0.010205	0.021525
OTU34 (Muribaculaceae)	0.004522	0.005664	0.011339	0.006768	0.023381
OTU12 (Muribaculaceae)	0.018083	0.032026	0.043691	0.101683	0.055963
OTU17 (Muribaculaceae)	0.000713	0.022012	0.015022	0.030435	0.028339
OTU10 (Muribaculaceae)	0.247296	0.083241	0.126249	0.070673	0.062258
OTU4 (Muribaculaceae)	0.073317	0.159817	0.112423	0.08828	0.082529
OTU23 (Muribaculaceae)	0.015551	0.029791	0.026248	0.010019	0.019375
	D1_7_3	D1_8_3	D1_10_3		
OTU3 (Lactobacillus)	0.049742	0.021967	0.090196		
OTU44 (Candidatus_Arthromitus)	0.000987	0.000583	0.012753		
OTU25 (Lactobacillus)	0.052362	0.038218	0.119601		
OTU26 (Bifidobacterium)	0.004292	0.019747	0.014766		
OTU501 (Muribaculaceae)	0.032558	0.033487	0.032134		
OTU27 (Muribaculaceae)	0.057468	0.03354	0.078845		
OTU46 (Muribaculaceae)	0.04377	0.023653	0.022796		
OTU933 (Muribaculaceae)	0.026905	0.018893	0.016356		
OTU35 (Lachnospiraceae_unclassified)	0.006349	0.100808	0.051135		
OTU292 (Muribaculaceae)	0.080468	0.076737	0.08952		
OTU32 (Muribaculum)	0.033364	0.041293	0.025636		
OTU20 (Lachnospiraceae_NK4A136_group)	0.02536	0.002935	0.047488		
OTU51 (Desulfovibrio)	0.002016	0.087583	0.037655		
OTU63 ([Eubacterium]_xylanophilum_group)	3.78E-05	0.064404	0.001894		
OTU56 (Mucispirillum)	0.004514	0.076507	0.004467		
OTU69 (Mucispirillum)	0.00257	0.049467	0.002421		
OTU41 (Muribaculaceae)	0.00771	0.002792	0.004944		
OTU33 (Bacteroides)	0.001071	0.000711	0.000419		
OTU21 (Muribaculaceae)	0.024029	0.009732	0.016229		
OTU422 (Muribaculaceae)	0.023411	0.004042	0.01138		
OTU14 (Muribaculaceae)	0.030794	0.012848	0.041479		
OTU24 (Muribaculaceae)	0.021316	0.038639	0.035881		

OTU9 (Muribaculaceae)	0.009226	0.020447	0.017418		
OTU34 (Muribaculaceae)	0.030647	0.019732	0.024913		
OTU12 (Muribaculaceae)	0.033855	0.047435	0.040326		
OTU17 (Muribaculaceae)	0.064351	0.032791	0.021852		
OTU10 (Muribaculaceae)	0.0856	0.046377	0.050535		
OTU4 (Muribaculaceae)	0.181833	0.065879	0.067819		
OTU23 (Muribaculaceae)	0.063394	0.00875	0.019142		

Data S5. Average abundance of gut microbiome at the phylum level in fC group			
ID	fC_0	fC_2W	fC_3W
Bacteroidota	0.6458823	0.708556444	0.636767667
Firmicutes	0.2268179	0.202716556	0.160348889
Verrucomicrobiota	0.089926	0.051051889	0.167764222
Desulfobacterota	0.0133056	0.020267222	0.008722444
Patescibacteria	0.0131024	0.003887778	0.008886333
Actinobacteriota	0.0047154	0.004315778	0.008685778
Deferribacterota	6.36E-04	0.006021222	0.006301444
Proteobacteria	0.0017548	0.002192444	0.001756889
Campilobacterota	0.0030793	8.17E-04	5.68E-04
Bacteria_unclassified	5.77E-04	7.53E-05	1.26E-04
Cyanobacteria	2.04E-04	8.30E-05	7.18E-05
Fusobacteriota	0	1.50E-05	0

Data S6. Average abundance of gut microbiome at the genus level in fC group			
ID	fC_0	fC_2W	fC_3W
Muribaculaceae	0.579644	0.633995	0.577246
Akkermansia	0.089926	0.051052	0.167764
Lactobacillus	0.125471	0.012956	0.025477
Lachnospiraceae_unclassified	0.028631	0.048978	0.043438
Lachnospiraceae_NK4A136_group	0.011285	0.063398	0.028793
Bacteroides	0.027239	0.027817	0.014311
Clostridia_UCG-014	0.016374	0.017809	0.012827
Desulfovibrio	0.01329	0.020254	0.008689
Alistipes	0.016867	0.01468	0.008423
Muribaculum	0.00613	0.011543	0.01591
Candidatus_Saccharimonas	0.013102	0.003888	0.008886
Odoribacter	0.006199	0.010798	0.004004
[Eubacterium]_xylanophilum_group	3.66E-04	0.01315	0.006579
Mucispirillum	6.36E-04	0.006021	0.006301
Prevotellaceae_UCG-001	0.004714	0.005348	0.002127
Candidatus_Arthromitus	0.009015	2.91E-04	6.63E-04
Prevotella	1.52E-04	4.88E-05	0.010542
Bacilli_unclassified	0.00586	0.001116	0.002598
Oscillospiraceae_unclassified	0.002854	0.003836	0.003101
Enterorhabdus	0.003674	0.004018	0.00193
Faecalibaculum	7.00E-04	0.006796	0.00203
Bifidobacterium	5.96E-04	5.18E-05	0.006413
Blautia	1.71E-04	5.45E-04	0.004679
Oscillospiraceae_uncultured	0.002244	0.001462	0.001211
Monoglobus	5.17E-04	0.003043	8.96E-04
Oscillospiraceae_UCG-005	2.92E-05	4.88E-04	0.003832
Marvinbryantia	0.001342	4.36E-04	1.06E-04
Others	0.032973	0.036182	0.031225

Data S7. Comparison of gut microbiome at the phylum level in fC group		
ID	Verrucomicrobiota	Deferribacterota
fC_0.median	0.0517(0.0142,0.1175)	<0.0001(<0.0001,0.0002)
fC_0.mean	0.089926	6.36E-04
fC_0.se	0.038103882	5.16E-04
fC_2W.median	0.0155(0.0105,0.0428)	0.0036(0.0031,0.0077)
fC_2W.mean	0.051051889	0.006021222
fC_2W.se	0.023781652	0.002179141
fC_3W.median	0.1423(0.1329,0.2268)	0.0017(0.0004,0.0067)
fC_3W.mean	0.167764222	0.006301444
fC_3W.se	0.026297339	0.003679902
p-value	0.010251024	0.002646034
z-score	-2.567244711	-3.00612131
Sig_mark	*	**
q-value	0.060403524	0.031752409
fixp	0.0103	0.0026
fixps	* 0.0103	** 0.0026

Data S8. Comparison of gut microbiome at the genus level in fC group			
ID	fC_0.median	fC_0.mean	fC_0.se
Akkermansia	0.0517(0.0142,0.1175)	0.089926	0.038103882
Lactobacillus	0.0782(0.029,0.2007)	0.1254713	0.038454271
Lachnospiraceae_NK4A136_group	0.0027(0.002,0.0214)	0.011285	0.004233991
Muribaculum	0.006(0.0048,0.007)	0.0061297	9.91E-04
Odoribacter	0.0062(0.0045,0.0076)	0.0061987	6.11E-04
[Eubacterium]_xylanophilum_group	<0.0001(<0.0001,0.0002)	3.66E-04	2.11E-04
Mucispirillum	<0.0001(<0.0001,0.0002)	6.36E-04	5.16E-04
Candidatus_Arthromitus	0.0081(0.0033,0.0129)	0.0090153	0.002260346
Bacilli_unclassified	0.0039(0.0018,0.0107)	0.0058595	0.001770588
Faecalibaculum	0.0001(<0.0001,0.0008)	7.00E-04	3.57E-04
Bifidobacterium	0.0002(<0.0001,0.0008)	5.96E-04	2.90E-04
Blautia	<0.0001(<0.0001,0.0003)	1.71E-04	6.75E-05
Oscillospiraceae_UCG-005	<0.0001(<0.0001,<0.0001)	2.92E-05	7.61E-06
ID	fC_2W.median	fC_2W.mean	fC_2W.se
Akkermansia	0.0155(0.0105,0.0428)	0.051051889	0.023781652
Lactobacillus	0.0085(0.0062,0.017)	0.012955889	0.003389523
Lachnospiraceae_NK4A136_group	0.0795(0.0155,0.0936)	0.063398333	0.01407456
Muribaculum	0.0117(0.0105,0.0145)	0.011542667	0.001398748
Odoribacter	0.0118(0.0066,0.0159)	0.010797556	0.002031356
[Eubacterium]_xylanophilum_group	0.0168(0.0018,0.0198)	0.01315	0.003103923
Mucispirillum	0.0036(0.0031,0.0077)	0.006021222	0.002179141
Candidatus_Arthromitus	0.0002(<0.0001,0.0004)	2.91E-04	9.00E-05
Bacilli_unclassified	0.0008(0.0004,0.0015)	0.001116111	3.26E-04
Faecalibaculum	0.0033(0.0022,0.0045)	0.006796111	0.003628143
Bifidobacterium	<0.0001(<0.0001,<0.0001)	5.18E-05	3.58E-05
Blautia	0.0006(0.0003,0.0007)	5.45E-04	1.03E-04
Oscillospiraceae_UCG-005	0.0003(0.0001,0.001)	4.88E-04	1.74E-04
ID	fC_4W.median	fC_4W.mean	fC_4W.se
Akkermansia	0.1423(0.1329,0.2268)	0.167764222	0.026297339
Lactobacillus	0.0174(0.0155,0.0327)	0.025476889	0.005812831
Lachnospiraceae_NK4A136_group	0.0289(0.0088,0.0418)	0.028792889	0.007071451
Muribaculum	0.0141(0.0097,0.0193)	0.015910444	0.002671392
Odoribacter	0.0027(0.0007,0.0052)	0.004003667	0.001495632
[Eubacterium]_xylanophilum_group	0.0016(0.0007,0.0046)	0.006578889	0.003460712
Mucispirillum	0.0017(0.0004,0.0067)	0.006301444	0.003679902
Candidatus_Arthromitus	0.0002(0.0001,0.0004)	6.63E-04	3.80E-04
Bacilli_unclassified	0.0025(0.0015,0.0031)	0.002597778	5.92E-04
Faecalibaculum	0.001(0.0006,0.0018)	0.002029778	0.001039794
Bifidobacterium	0.0042(0.001,0.0081)	0.006413444	0.00261519
Blautia	0.0006(0.0003,0.0026)	0.004679111	0.003536548
Oscillospiraceae_UCG-005	<0.0001(<0.0001,<0.0001)	0.003832111	0.003752099
ID	p-value	z-score	Sig_mark

Akkermansia	0.010251024	-2.567244711	*
Lactobacillus	0.017890577	-2.367875042	*
Lachnospiraceae_NK4A136_group	0.005642534	-2.767862008	**
Muribaculum	0.001726359	-3.13366823	**
Odoribacter	0.024155702	-2.254643699	*
[Eubacterium]_xylanophilum_group	0.003127547	-2.954915459	**
Mucispirillum	0.002646034	-3.00612131	**
Candidatus_Arthromitus	0.003241096	-2.943895309	**
Bacilli_unclassified	0.038580504	-2.068633371	*
Faecalibaculum	0.004502191	-2.840648452	**
Bifidobacterium	5.51E-04	-3.454619515	***
Blautia	0.007322667	-2.68180255	**
Oscillospiraceae_UCG-005	0.00349461	-2.920508237	**
ID	q-value	fixp	fixps
Akkermansia	0.085626201	0.0103	* 0.0103
Lactobacillus	0.115475544	0.0179	* 0.0179
Lachnospiraceae_NK4A136_group	0.061633834	0.0056	** 0.0056
Muribaculum	0.061633834	0.0017	** 0.0017
Odoribacter	0.134585775	0.0242	* 0.0242
[Eubacterium]_xylanophilum_group	0.061633834	0.0031	** 0.0031
Mucispirillum	0.061633834	0.0026	** 0.0026
Candidatus_Arthromitus	0.061633834	0.0032	** 0.0032
Bacilli_unclassified	0.182614385	0.0386	* 0.0386
Faecalibaculum	0.061633834	0.0045	** 0.0045
Bifidobacterium	0.055585674	6.00E-04	***0.0006
Blautia	0.074272761	0.0073	** 0.0073
Oscillospiraceae_UCG-005	0.061633834	0.0035	** 0.0035

**Data S9. Comparison of gut microbiome at the phylum level between fC and CPNs at week 2**	
ID	Deferribacterota
fC_0.median	<0.0001(<0.0001,0.0002)
fC_0.mean	6.36E-04
fC_0.se	5.16E-04
fC_2W.median	0.0036(0.0031,0.0077)
fC_2W.mean	0.006021222
fC_2W.se	0.002179141
CPNs_0.median	<0.0001(<0.0001,0.0007)
CPNs_0.mean	0.00159
CPNs_0.se	0.00114155
CPNs_2W.median	0.0016(0.0006,0.006)
CPNs_2W.mean	0.002881556
CPNs_2W.se	9.25E-04
p-value	0.002061651
z-score	-3.081204395
Sig_mark	**
q-value	0.024739809
fixp	0.0021
fixps	** 0.0021

**Data S10. Comparison of gut microbiome at the genus level between fC and CPNs at week 2**			
ID	fC_0.median	fC_0.mean	fC_0.se
Lactobacillus	0.0782(0.029,0.2007)	0.1254713	0.038454271
Lachnospiraceae_NK4A136_group	0.0027(0.002,0.0214)	0.011285	0.004233991
Muribaculum	0.006(0.0048,0.007)	0.0061297	9.91E-04
[Eubacterium]_xylanophilum_group	<0.0001(<0.0001,0.0002)	3.66E-04	2.11E-04
Candidatus_Arthromitus	0.0081(0.0033,0.0129)	0.0090153	0.002260346
Bacilli_unclassified	0.0039(0.0018,0.0107)	0.0058595	0.001770588
Mucispirillum	<0.0001(<0.0001,0.0002)	6.36E-04	5.16E-04
Faecalibaculum	0.0001(<0.0001,0.0008)	7.00E-04	3.57E-04
Blautia	<0.0001(<0.0001,0.0003)	1.71E-04	6.75E-05
ID	fC_2W.median	fC_2W.mean	fC_2W.se
Lactobacillus	0.0085(0.0062,0.017)	0.012955889	0.003389523
Lachnospiraceae_NK4A136_group	0.0795(0.0155,0.0936)	0.063398333	0.01407456
Muribaculum	0.0117(0.0105,0.0145)	0.011542667	0.001398748
[Eubacterium]_xylanophilum_group	0.0168(0.0018,0.0198)	0.01315	0.003103923
Candidatus_Arthromitus	0.0002(<0.0001,0.0004)	2.91E-04	9.00E-05
Bacilli_unclassified	0.0008(0.0004,0.0015)	0.001116111	3.26E-04
Mucispirillum	0.0036(0.0031,0.0077)	0.006021222	0.002179141
Faecalibaculum	0.0033(0.0022,0.0045)	0.006796111	0.003628143
Blautia	0.0006(0.0003,0.0007)	5.45E-04	1.03E-04
ID	CPNs_0.median	CPNs_0.mean	CPNs_0.se
Lactobacillus	0.0444(0.0272,0.1168)	0.079416222	0.021038941
Lachnospiraceae_NK4A136_group	0.0034(0.0011,0.0131)	0.010933667	0.005632348
Muribaculum	0.0066(0.0059,0.009)	0.008379222	0.001104975
[Eubacterium]_xylanophilum_group	<0.0001(<0.0001,0.0002)	8.36E-04	7.14E-04
Candidatus_Arthromitus	0.0048(0.0019,0.0077)	0.005233222	0.001416255
Bacilli_unclassified	0.002(0.0017,0.0035)	0.002556889	4.58E-04
Mucispirillum	<0.0001(<0.0001,0.0007)	0.00159	0.00114155
Faecalibaculum	0.0001(<0.0001,0.0002)	1.23E-04	2.65E-05
Blautia	0.0001(<0.0001,0.0004)	2.17E-04	8.00E-05
ID	CPNs_2W.median	CPNs_2W.mean	CPNs_2W.se
Lactobacillus	0.0164(0.011,0.0789)	0.060007889	0.028068391
Lachnospiraceae_NK4A136_group	0.0325(0.0254,0.0502)	0.036364	0.008345238
Muribaculum	0.0103(0.0086,0.0128)	0.010463111	0.001677835
[Eubacterium]_xylanophilum_group	0.0027(0.0005,0.0032)	0.004267222	0.001860684
Candidatus_Arthromitus	0.0004(0.0002,0.0007)	6.21E-04	2.14E-04
Bacilli_unclassified	0.0017(0.0009,0.0026)	0.004306333	0.002585151
Mucispirillum	0.0016(0.0006,0.006)	0.002881556	9.25E-04
Faecalibaculum	0.0002(0.0002,0.0008)	4.12E-04	1.10E-04
Blautia	0.0005(0.0002,0.0005)	0.001813222	0.001373603

ID	p-value	z-score	Sig_mark
Lactobacillus	0.009636596	-2.58860358	**
Lachnospiraceae_NK4A136_group	0.003622384	-2.90930062	**
Muribaculum	0.032606436	-2.13689619	*
[Eubacterium]_xylanophilum_group	1.19E-04	-3.84859067	***
Candidatus_Arthromitus	1.01E-04	-3.88830523	***
Bacilli_unclassified	0.042432871	-2.02924963	*
Mucispirillum	0.002061651	-3.0812044	**
Faecalibaculum	5.11E-04	-3.47513779	***
Blautia	0.02132683	-2.30214795	*
ID	q-value	fixp	fixps
Lactobacillus	0.067963362	0.0096	** 0.0096
Lachnospiraceae_NK4A136_group	0.034671386	0.0036	** 0.0036
Muribaculum	0.167539861	0.0326	* 0.0326
[Eubacterium]_xylanophilum_group	0.005523198	1.00E-04	***0.0001
Candidatus_Arthromitus	0.005523198	1.00E-04	***0.0001
Bacilli_unclassified	0.189533492	0.0424	* 0.0424
Mucispirillum	0.025114654	0.0021	** 0.0021
Faecalibaculum	0.013683839	5.00E-04	***0.0005
Blautia	0.124251966	0.0213	* 0.0213

Data S11. Comparison of gut microbiome at the phylum level between fC and CPNs at week 3			
ID	fC_0.median	fC_0.mean	fC_0.se
Verrucomicrobiota	0.0517(0.0142,0.1175)	0.089926	0.038103882
Actinobacteriota	0.0033(0.0008,0.0064)	0.0047154	0.001490541
Deferribacterota	<0.0001(<0.0001,0.0002)	6.36E-04	5.16E-04
ID	fC_3W.median	fC_3W.mean	fC_3W.se
Verrucomicrobiota	0.1423(0.1329,0.2268)	0.167764222	0.026297339
Actinobacteriota	0.0076(0.0035,0.0101)	0.008685778	0.002671883
Deferribacterota	0.0017(0.0004,0.0067)	0.006301444	0.003679902
ID	CPNs_0.median	CPNs_0.mean	CPNs_0.se
Verrucomicrobiota	0.0533(0.0375,0.1079)	0.072075889	0.01860228
Actinobacteriota	0.001(0.0009,0.0029)	0.002275778	7.36E-04
Deferribacterota	<0.0001(<0.0001,0.0007)	0.00159	0.00114155
ID	CPNs_3W.median	CPNs_3W.mean	CPNs_3W.se
Verrucomicrobiota	0.1435(0.0696,0.1919)	0.134962111	0.026950626
Actinobacteriota	0.0049(0.0044,0.017)	0.009841111	0.002720622
Deferribacterota	0.0036(0.0003,0.0079)	0.004578333	0.001690434
ID	p-value	z-score	Sig_mark
Verrucomicrobiota	0.044129562	-2.01285808	*
Actinobacteriota	0.021675332	-2.2960096	*
Deferribacterota	0.010180977	-2.5696212	*
ID	q-value	fixp	fixps
Verrucomicrobiota	0.124348474	0.0441	* 0.0441
Actinobacteriota	0.119214329	0.0217	* 0.0217
Deferribacterota	0.111990749	0.0102	* 0.0102

Data S12. Comparison of gut microbiome at the genus level between fC and CPNs at week 3			
ID	fC_0.median	fC_0.mean	fC_0.se
Akkermansia	0.0517(0.0142,0.1175)	0.089926	0.038103882
Lachnospiraceae_NK4A136_group	0.0027(0.002,0.0214)	0.011285	0.004233991
Muribaculum	0.006(0.0048,0.007)	0.0061297	9.91E-04
Candidatus_Arthromitus	0.0081(0.0033,0.0129)	0.0090153	0.002260346
Bifidobacterium	0.0002(<0.0001,0.0008)	5.96E-04	2.90E-04
Mucispirillum	<0.0001(<0.0001,0.0002)	6.36E-04	5.16E-04
Rikenella	0.003(0.0021,0.0033)	0.0031379	4.91E-04
Prevotella	<0.0001(<0.0001,0.0002)	1.52E-04	6.32E-05
Blautia	<0.0001(<0.0001,0.0003)	1.71E-04	6.75E-05
Faecalibaculum	0.0001(<0.0001,0.0008)	7.00E-04	3.57E-04
ID	fC_3W.median	fC_3W.mean	fC_3W.se
Akkermansia	0.1423(0.1329,0.2268)	0.167764222	0.026297339
Lachnospiraceae_NK4A136_group	0.0289(0.0088,0.0418)	0.028792889	0.007071451
Muribaculum	0.0141(0.0097,0.0193)	0.015910444	0.002671392
Candidatus_Arthromitus	0.0002(0.0001,0.0004)	6.63E-04	3.80E-04
Bifidobacterium	0.0042(0.001,0.0081)	0.006413444	0.00261519
Mucispirillum	0.0017(0.0004,0.0067)	0.006301444	0.003679902
Rikenella	0.001(0.0009,0.0017)	0.001424444	3.60E-04
Prevotella	<0.0001(<0.0001,<0.0001)	0.010542111	0.010535487
Blautia	0.0006(0.0003,0.0026)	0.004679111	0.003536548
Faecalibaculum	0.001(0.0006,0.0018)	0.002029778	0.001039794
ID	CPNs_0.median	CPNs_0.mean	CPNs_0.se
Akkermansia	0.0533(0.0375,0.1079)	0.072075889	0.01860228
Lachnospiraceae_NK4A136_group	0.0034(0.0011,0.0131)	0.010933667	0.005632348
Muribaculum	0.0066(0.0059,0.009)	0.008379222	0.001104975
Candidatus_Arthromitus	0.0048(0.0019,0.0077)	0.005233222	0.001416255
Bifidobacterium	<0.0001(<0.0001,<0.0001)	8.11E-06	5.38E-06
Mucispirillum	<0.0001(<0.0001,0.0007)	0.00159	0.00114155
Rikenella	0.0031(0.0019,0.0039)	0.003327333	6.85E-04
Prevotella	<0.0001(<0.0001,<0.0001)	4.12E-05	1.80E-05
Blautia	0.0001(<0.0001,0.0004)	2.17E-04	8.00E-05
Faecalibaculum	0.0001(<0.0001,0.0002)	1.23E-04	2.65E-05
ID	CPNs_3W.median	CPNs_3W.mean	CPNs_3W.se
Akkermansia	0.1435(0.0696,0.1919)	0.134962111	0.026950626
Lachnospiraceae_NK4A136_group	0.022(0.0065,0.0484)	0.026866333	0.007531746
Muribaculum	0.0029(0.0019,0.015)	0.007977222	0.002368263
Candidatus_Arthromitus	0.0004(0.0002,0.0005)	5.50E-04	2.12E-04
Bifidobacterium	0.003(0.0012,0.0138)	0.007399889	0.002633128
Mucispirillum	0.0036(0.0003,0.0079)	0.004578333	0.001690434

Rikenella	0.0021(0.0019,0.0024)	0.003399111	0.001015483
Prevotella	<0.0001(<0.0001,<0.0001)	1.33E-06	1.33E-06
Blautia	0.0008(0.0005,0.0012)	0.001113556	3.65E-04
Faecalibaculum	0.0002(<0.0001,0.0015)	8.20E-04	3.25E-04
ID	p-value	z-score	Sig_mark
Akkermansia	0.044129562	-2.012858075	*
Lachnospiraceae_NK4A136_group	0.033774714	-2.122751102	*
Muribaculum	0.012145385	-2.507891801	*
Candidatus_Arthromitus	2.98E-04	-3.61670617	***
Bifidobacterium	3.63E-05	-4.13003873	***
Mucispirillum	0.010180977	-2.569621199	*
Rikenella	0.022536364	-2.281204231	*
Prevotella	0.00927845	-2.601620366	**
Blautia	0.003897877	-2.886310297	**
Faecalibaculum	0.035153096	-2.106590446	*
ID	q-value	fixp	fixps
Akkermansia	0.211439573	0.0441	* 0.0441
Lachnospiraceae_NK4A136_group	0.190850399	0.0338	* 0.0338
Muribaculum	0.107031206	0.0121	* 0.0121
Candidatus_Arthromitus	0.021035485	0.0003	***0.0003
Bifidobacterium	0.0051141	<0.0001	***<0.0001
Mucispirillum	0.102536984	0.0102	* 0.0102
Rikenella	0.176534855	0.0225	* 0.0225
Prevotella	0.100840658	0.0093	** 0.0093
Blautia	0.07851437	0.0039	** 0.0039
Faecalibaculum	0.190850399	0.0352	* 0.0352

Data S13. Heatmap of fC group and CPNs group at week 2					
	D1_1_1	D1_9_1	D1_8_1	D1_7_1	D1_6_1
OTU6 (Muribaculaceae)	0.044895	0.058752	0.065149	0.024267	0.062085
OTU292 (Muribaculaceae)	0.054686	0.059323	0.059807	0.022217	0.055905
OTU8 (Muribaculaceae)	0.05995	0.102	0.065965	0.050475	0.069513
OTU27 (Muribaculaceae)	0.005358	0.010796	0.025736	0.0004	0.008574
OTU5 (Muribaculaceae)	0.091836	0.152052	0.161005	0.097864	0.164647
OTU501 (Muribaculaceae)	0.027012	0.039946	0.041654	0.029055	0.048209
OTU21 (Muribaculaceae)	0.034027	0.044604	0.033153	0.089291	0.027975
OTU422 (Muribaculaceae)	0.031091	0.038722	0.039453	0.05854	0.022405
OTU414 (Muribaculaceae)	0.154907	0.018792	0.080916	0.102031	0.105987
OTU13 (Muribaculaceae)	0.063295	0.083125	0.06293	0.02958	0.077588
OTU44 (Candidatus_Arthromitus)	0.02843	0.030639	0.007658	0.011139	0.016252
OTU9 (Muribaculaceae)	0.039548	0.068096	0.05017	0.004344	0.054016
OTU87 (Clostridia_UCG-014)	0.005231	0.014216	0.00023	0.00183	0.000977
OTU94 (Clostridia_UCG-014)	0.000181	0	0	0.00059	0
OTU11 (Muribaculaceae)	0.029283	0.012568	0.05568	0.061175	0.035223
OTU10 (Muribaculaceae)	0.057189	0.077997	0.074541	0.019053	0.080514
OTU23 (Muribaculaceae)	0.025524	0.045997	0.038612	0.05268	0.035635
OTU56 (Mucispirillum)	0	0.002311	9.57E-05	0.000133	0.000484
OTU63 ([Eubacterium]_xylanophilum_group)	0	0.000237	0.002663	0.00028	0
OTU130 (Lachnospiraceae_NK4A136_group)	0	0.001639	9.22E-05	0	0
OTU54 (Lachnospiraceae_NK4A136_group)	0.000293	0.028807	0.000188	0.000301	0.004243
OTU37 (Lachnospiraceae_unclassified)	0.001382	0.019395	0.000188	0.000887	0.000304
OTU32 (Muribaculum)	0.027737	0.008576	0.020248	0.018536	0.015533
OTU34 (Muribaculaceae)	0.010169	0.009645	0.011522	0.027698	0.03839
OTU45 (Faecalibaculum)	0.00029	0.004644	0.007637	0	0.00966
OTU15 (Bacteroides)	0.102565	0.009353	0.022169	0.048159	0.015154
OTU33 (Bacteroides)	0.069433	0.001594	0.003404	0.145407	0.001371
OTU12 (Muribaculaceae)	0.030005	0.038238	0.057102	0.0742	0.041102
OTU40 (Muribaculaceae)	0.005682	0.017934	0.012033	0.029868	0.008252
	D1_10_1	D1_4_1	D1_3_1	D1_2_1	D1_5_1
OTU6 (Muribaculaceae)	0.087574	0.052792	0.02867	0.018017	0.109896
OTU292 (Muribaculaceae)	0.085394	0.048231	0.030574	0.018306	0.115614
OTU8 (Muribaculaceae)	0.094129	0.056821	0.099178	0.16185	0.072452
OTU27 (Muribaculaceae)	0.047272	0.005036	0.006494	0.020393	0.018079
OTU5 (Muribaculaceae)	0.182999	0.170529	0.142045	0.182454	0.185319
OTU501 (Muribaculaceae)	0.050123	0.032523	0.032029	0.045966	0.043105
OTU21 (Muribaculaceae)	0.034951	0.007243	0.037573	0.032391	0.018187
OTU422 (Muribaculaceae)	0.021557	0.032309	0.030917	0.030836	0.017952
OTU414 (Muribaculaceae)	0.026264	0.083716	0.140654	0.13367	0.07593
OTU13 (Muribaculaceae)	0.04867	0.083193	0.055656	0.038185	0.06381
OTU44 (Candidatus_Arthromitus)	0	0.032347	0.064805	0.0468	0.049071

OTU9 (Muribaculaceae)	0.046782	0.032274	0.055624	0.04908	0.061396
OTU87 (Clostridia_UCG-014)	0	0.002411	0.000232	0.003649	0.000445
OTU94 (Clostridia_UCG-014)	0	0.000809	0	0	0
OTU11 (Muribaculaceae)	0.029652	0.046934	0.035584	0.107024	0.023971
OTU10 (Muribaculaceae)	0.040199	0.109307	0.059563	0.046844	0.035203
OTU23 (Muribaculaceae)	0.016333	0.038482	0.025974	0.016859	0.010978
OTU56 (Mucispirillum)	0.000107	0.000252	0.009055	9.87E-05	0
OTU63 ([Eubacterium]_xylanophilum_group)	9.87E-05	0.004287	6.16E-05	0.000296	0.000268
OTU130 (Lachnospiraceae_NK4A136_group)	0	0.020521	0.00125	0.002958	0.002885
OTU54 (Lachnospiraceae_NK4A136_group)	8.29E-05	0.034588	0.002226	0.00015	0.000395
OTU37 (Lachnospiraceae_unclassified)	0.003412	0.000966	0.001904	0.000283	0.000596
OTU32 (Muribaculum)	0.013481	0.017738	0.014367	0.00533	0.011569
OTU34 (Muribaculaceae)	0.008324	0.01696	0.015661	0.005034	0.026967
OTU45 (Faecalibaculum)	0	0.000466	0	0	0.000936
OTU15 (Bacteroides)	0.098418	0.033851	0.03928	0.023616	0.012234
OTU33 (Bacteroides)	0.002488	0.01227	0.054357	0.003264	0.001786
OTU12 (Muribaculaceae)	0.032266	0.009399	0.007069	0.004483	0.020633
OTU40 (Muribaculaceae)	0.029423	0.013743	0.009199	0.002165	0.020324
	D1_3_2	D1_10_2	D1_9_2	D1_8_2	D1_7_2
OTU6 (Muribaculaceae)	0.0675	0.039411	0.064609	0.055601	0.019908
OTU292 (Muribaculaceae)	0.059915	0.039803	0.062926	0.056747	0.022786
OTU8 (Muribaculaceae)	0.046434	0.036375	0.07517	0.046875	0.022178
OTU27 (Muribaculaceae)	0.005189	0.00348	0.001388	0.001056	7.60E-05
OTU5 (Muribaculaceae)	0.081699	0.076648	0.098553	0.072672	0.169271
OTU501 (Muribaculaceae)	0.019825	0.015147	0.027171	0.015956	0.025058
OTU21 (Muribaculaceae)	0.023557	0.01271	0.008962	0.023591	0.042596
OTU422 (Muribaculaceae)	0.01297	0.015812	0.01362	0.015906	0.029651
OTU414 (Muribaculaceae)	0.076181	0.091087	0.05011	0.085289	0.014693
OTU13 (Muribaculaceae)	0.036847	0.055195	0.052433	0.044372	0.049393
OTU44 (Candidatus_Arthromitus)	0.001079	0.000245	0.000597	0.001827	0.000123
OTU9 (Muribaculaceae)	0.031743	0.05732	0.042189	0.014261	0.042517
OTU87 (Clostridia_UCG-014)	0.001431	0.000291	0.000723	0.001476	0
OTU94 (Clostridia_UCG-014)	0.000141	0	0.00363	0.005107	6.88E-05
OTU11 (Muribaculaceae)	0.040049	0.059904	0.056912	0.044306	0.144154
OTU10 (Muribaculaceae)	0.068954	0.127939	0.094836	0.073456	0.082189
OTU23 (Muribaculaceae)	0.057599	0.048809	0.050314	0.066858	0.092054
OTU56 (Mucispirillum)	0.013985	0.00118	0.01362	0.01038	0.006992
OTU63 ([Eubacterium]_xylanophilum_group)	0.045745	0.002721	0.048852	0.046146	0.006236
OTU130 (Lachnospiraceae_NK4A136_group)	0.042685	0.00091	0.005375	0.037195	0.006771
OTU54 (Lachnospiraceae_NK4A136_group)	0.011807	0.00368	0.018359	0.010276	0.006395
OTU37 (Lachnospiraceae_unclassified)	0.020913	0.002661	0.023967	0.017758	0.003691
OTU32 (Muribaculum)	0.038436	0.030248	0.022293	0.03596	0.00785
OTU34 (Muribaculaceae)	0.040389	0.068619	0.032021	0.061873	0.012109
OTU45 (Faecalibaculum)	0.006421	0.0305	0.014596	0.006748	0.001151

OTU15 (Bacteroides)	0.049994	0.059257	0.036348	0.054438	0.044938
OTU33 (Bacteroides)	0.034877	0.025311	0.005916	0.036906	0.042705
OTU12 (Muribaculaceae)	0.054852	0.07156	0.057694	0.048564	0.08643
OTU40 (Muribaculaceae)	0.008784	0.023175	0.016818	0.008399	0.018016
	D1_6_2	D1_5_2	D1_4_2	D1_2_2	ND1_10_1
OTU6 (Muribaculaceae)	0.074477	0.050745	0.04487	0.062599	0.096649
OTU292 (Muribaculaceae)	0.073948	0.055831	0.05067	0.06579	0.092823
OTU8 (Muribaculaceae)	0.044857	0.033866	0.086433	0.045054	0.074368
OTU27 (Muribaculaceae)	0.000626	0.037359	0.001129	6.55E-05	0.016581
OTU5 (Muribaculaceae)	0.099999	0.067234	0.071091	0.076906	0.160847
OTU501 (Muribaculaceae)	0.024465	0.015453	0.023034	0.018377	0.062237
OTU21 (Muribaculaceae)	0.025615	0.006646	0.005786	0.029493	0.017837
OTU422 (Muribaculaceae)	0.025446	0.005129	0.009576	0.017283	0.013132
OTU414 (Muribaculaceae)	0.116717	0.113759	0.043012	0.081474	0.081915
OTU13 (Muribaculaceae)	0.082164	0.041578	0.031453	0.046091	0.089533
OTU44 (Candidatus_Arthromitus)	0.002091	6.71E-05	0.000469	0.001254	0.003622
OTU9 (Muribaculaceae)	0.028344	0.065371	0.0497	0.011104	0.040395
OTU87 (Clostridia_UCG-014)	0.000342	0.000502	0.002613	0.001925	5.98E-05
OTU94 (Clostridia_UCG-014)	0.00168	0	0.014291	0.000137	0.002107
OTU11 (Muribaculaceae)	0.101961	0.04681	0.06954	0.033974	0.046495
OTU10 (Muribaculaceae)	0.057612	0.073359	0.109354	0.066413	0.059559
OTU23 (Muribaculaceae)	0.02866	0.025881	0.06424	0.061789	0.015254
OTU56 (Mucispirillum)	0.006746	0.000701	0.005494	0.037093	0
OTU63 ([Eubacterium]_xylanophilum_group)	0.001015	0.003575	0.063726	0.039313	0
OTU130 (Lachnospiraceae_NK4A136_group)	0.000279	0.000738	0.010396	0.062817	0.000146
OTU54 (Lachnospiraceae_NK4A136_group)	0.001041	0.001288	0.023285	0.011676	0.000266
OTU37 (Lachnospiraceae_unclassified)	0.000744	0.000121	0.03761	0.013943	0.000445
OTU32 (Muribaculum)	0.03094	0.026388	0.02685	0.039101	0.025512
OTU34 (Muribaculaceae)	0.058645	0.10383	0.032314	0.030911	0.019815
OTU45 (Faecalibaculum)	0.008794	0.094	0.012515	0.003337	0.000771
OTU15 (Bacteroides)	0.017999	0.047848	0.03793	0.049606	0.016973
OTU33 (Bacteroides)	0.019261	0.014331	0.007229	0.035866	0.001195
OTU12 (Muribaculaceae)	0.050401	0.057694	0.055759	0.053685	0.045013
OTU40 (Muribaculaceae)	0.015129	0.009897	0.00963	0.002923	0.016447
	ND1_9_1	ND1_8_1	ND1_7_1	ND1_6_1	ND1_4_1
OTU6 (Muribaculaceae)	0.06655	0.075567	0.066255	0.097613	0.105801
OTU292 (Muribaculaceae)	0.073207	0.07683	0.060728	0.097653	0.100149
OTU8 (Muribaculaceae)	0.062287	0.07708	0.053287	0.069591	0.075145
OTU27 (Muribaculaceae)	0.021884	0.014445	0.011646	0.01393	0.009648
OTU5 (Muribaculaceae)	0.215558	0.188675	0.27512	0.167716	0.15337
OTU501 (Muribaculaceae)	0.035494	0.04081	0.073165	0.057186	0.042131
OTU21 (Muribaculaceae)	0.024321	0.026627	0.019827	0.019152	0.024373
OTU422 (Muribaculaceae)	0.018148	0.016864	0.01701	0.014922	0.028452

OTU414 (Muribaculaceae)	0.059602	0.084022	0.099189	0.098323	0.057352
OTU13 (Muribaculaceae)	0.076541	0.070743	0.078635	0.073996	0.056033
OTU44 (Candidatus_Arthromitus)	0.006467	0.025101	0.019474	0.018145	0.03473
OTU9 (Muribaculaceae)	0.024653	0.047927	0.036399	0.031401	0.018294
OTU87 (Clostridia_UCG-014)	0.001676	0.000104	0.001149	0.000297	0.000601
OTU94 (Clostridia_UCG-014)	0.000157	0	0.00123	0.001087	0.000244
OTU11 (Muribaculaceae)	0.027783	0.024053	0.045646	0.028701	0.026134
OTU10 (Muribaculaceae)	0.047295	0.05871	0.036692	0.051691	0.074775
OTU23 (Muribaculaceae)	0.03019	0.024517	0.017546	0.015561	0.025022
OTU56 (Mucispirillum)	0.001585	0.023176	0.003581	0	0
OTU63 ([Eubacterium]_xylanophilum_group)	0.018308	0.000568	0.000196	0.000266	4.28E-05
OTU130 (Lachnospiraceae_NK4A136_group)	0.007636	0.000484	0.000212	0.000116	7.55E-05
OTU54 (Lachnospiraceae_NK4A136_group)	0.003075	0.001325	0.000191	0.000701	0.000695
OTU37 (Lachnospiraceae_unclassified)	0.014316	0.002899	0.000535	0.00038	0.000367
OTU32 (Muribaculum)	0.014741	0.014315	0.010908	0.017869	0.017003
OTU34 (Muribaculaceae)	0.025807	0.023446	0.016127	0.021135	0.010279
OTU45 (Faecalibaculum)	0.000551	0.00012	3.92E-05	0.000429	0.000307
OTU15 (Bacteroides)	0.029068	0.027829	0.01504	0.059671	0.03432
OTU33 (Bacteroides)	0.001443	0.009838	0.006098	0.003327	0.00199
OTU12 (Muribaculaceae)	0.064437	0.030215	0.025537	0.022702	0.079199
OTU40 (Muribaculaceae)	0.02722	0.013708	0.008536	0.01644	0.023467
	ND1_3_1	ND1_2_1	ND1_1_1	ND1_8_2	ND1_7_2
OTU6 (Muribaculaceae)	0.085892	0.038096	0.096813	0.068	0.07362
OTU292 (Muribaculaceae)	0.080241	0.03345	0.085847	0.06029	0.069336
OTU8 (Muribaculaceae)	0.061006	0.076773	0.085499	0.068764	0.04632
OTU27 (Muribaculaceae)	0.007054	0.01344	0.014822	0.011015	0.000795
OTU5 (Muribaculaceae)	0.158742	0.231022	0.175529	0.1512	0.078551
OTU501 (Muribaculaceae)	0.051784	0.047544	0.068391	0.020752	0.025234
OTU21 (Muribaculaceae)	0.013863	0.035573	0.021091	0.022421	0.018842
OTU422 (Muribaculaceae)	0.014717	0.035392	0.015518	0.017275	0.011344
OTU414 (Muribaculaceae)	0.074936	0.061931	0.051073	0.060548	0.098557
OTU13 (Muribaculaceae)	0.078282	0.05772	0.060753	0.05359	0.05179
OTU44 (Candidatus_Arthromitus)	0.003413	0.016452	0.00554	0.000268	0.004688
OTU9 (Muribaculaceae)	0.028568	0.044638	0.030484	0.041077	0.112562
OTU87 (Clostridia_UCG-014)	0.000454	0.006419	0.001469	0.0042	0.004174
OTU94 (Clostridia_UCG-014)	0.000317	0.001015	0.000873	0.026575	0.003286
OTU11 (Muribaculaceae)	0.033406	0.027421	0.03144	0.06743	0.098128
OTU10 (Muribaculaceae)	0.057412	0.070781	0.092075	0.067903	0.078886
OTU23 (Muribaculaceae)	0.026978	0.019726	0.024224	0.04724	0.029485
OTU56 (Mucispirillum)	0.000213	0.000632	7.08E-05	0.002937	0.007536
OTU63 ([Eubacterium]_xylanophilum_group)	0	0.001176	0	0.00557	0.001258
OTU130 (Lachnospiraceae_NK4A136_group)	9.52E-05	0.000738	7.08E-05	0.004609	0.002593
OTU54 (Lachnospiraceae_NK4A136_group)	0.000203	0.001275	0	0.017756	0.000952
OTU37 (Lachnospiraceae_unclassified)	0.00026	0.000243	0.000599	0.008553	0.001913

OTU32 (Muribaculum)	0.027149	0.020954	0.010768	0.02531	0.050719
OTU34 (Muribaculaceae)	0.014759	0.014083	0.00901	0.029911	0.034585
OTU45 (Faecalibaculum)	0.000283	0.000649	0.000192	0.001276	0.000791
OTU15 (Bacteroides)	0.043822	0.037747	0.042279	0.025335	0.030526
OTU33 (Bacteroides)	0.004565	0.003575	0.001764	0.011301	0.014438
OTU12 (Muribaculaceae)	0.09731	0.057703	0.049227	0.066533	0.03983
OTU40 (Muribaculaceae)	0.034276	0.043832	0.024578	0.012357	0.009262
	ND1_6_2	ND1_1_2	ND1_3_2	ND1_2_2	ND1_9_2
OTU6 (Muribaculaceae)	0.070065	0.102773	0.074124	0.097397	0.09515
OTU292 (Muribaculaceae)	0.068542	0.098485	0.078034	0.090033	0.087077
OTU8 (Muribaculaceae)	0.074993	0.084279	0.0639	0.052295	0.046496
OTU27 (Muribaculaceae)	9.03E-05	0.010818	0.007973	0.012737	0.001493
OTU5 (Muribaculaceae)	0.070568	0.216728	0.155746	0.18775	0.12656
OTU501 (Muribaculaceae)	0.019265	0.059892	0.054891	0.062546	0.055366
OTU21 (Muribaculaceae)	0.031519	0.029199	0.017228	0.012274	0.022003
OTU422 (Muribaculaceae)	0.024279	0.018373	0.015175	0.011389	0.012057
OTU414 (Muribaculaceae)	0.075248	0.057023	0.079615	0.056487	0.062842
OTU13 (Muribaculaceae)	0.048794	0.047205	0.049735	0.040042	0.073151
OTU44 (Candidatus_Arthromitus)	0.000938	0.001452	0.001991	0.007499	0.000532
OTU9 (Muribaculaceae)	0.033772	0.030896	0.050924	0.078528	0.059349
OTU87 (Clostridia_UCG-014)	0.000703	0.000598	0.002924	0.006689	0.003368
OTU94 (Clostridia_UCG-014)	0	0	0.036299	0.001039	0.013643
OTU11 (Muribaculaceae)	0.117116	0.053465	0.048783	0.045995	0.07214
OTU10 (Muribaculaceae)	0.13487	0.061454	0.058351	0.033134	0.077069
OTU23 (Muribaculaceae)	0.075346	0.02443	0.03374	0.017418	0.028375
OTU56 (Mucispirillum)	0.017444	0.001989	0.001049	0.001159	0.012328
OTU63 ([Eubacterium]_xylanophilum_group)	0.005854	0.00185	0.000579	0.007947	0.002252
OTU130 (Lachnospiraceae_NK4A136_group)	0.001743	0.000231	0.003144	0.001385	0.000526
OTU54 (Lachnospiraceae_NK4A136_group)	0.009796	0	0.012893	0.021151	0.003856
OTU37 (Lachnospiraceae_unclassified)	0.004162	0.00131	0.003984	0.005672	0.002625
OTU32 (Muribaculum)	0.009501	0.010911	0.020012	0.020845	0.024453
OTU34 (Muribaculaceae)	0.023549	0.008391	0.042138	0.026966	0.03456
OTU45 (Faecalibaculum)	0.000927	0	0.002328	0.00043	0.002556
OTU15 (Bacteroides)	0.00903	0.027544	0.023436	0.037961	0.018152
OTU33 (Bacteroides)	0.004507	0.01174	0.009895	0.02604	0.00353
OTU12 (Muribaculaceae)	0.059009	0.02711	0.034846	0.027042	0.037048
OTU40 (Muribaculaceae)	0.00837	0.011854	0.016265	0.010149	0.021443
	ND1_5_2	ND1_10_2			
OTU6 (Muribaculaceae)	0.032843	0.039031			
OTU292 (Muribaculaceae)	0.031199	0.041059			
OTU8 (Muribaculaceae)	0.049505	0.041316			
OTU27 (Muribaculaceae)	0.019896	0.000456			
OTU5 (Muribaculaceae)	0.117967	0.079774			

OTU501 (Muribaculaceae)	0.033734	0.020955			
OTU21 (Muribaculaceae)	0.023434	0.021039			
OTU422 (Muribaculaceae)	0.016572	0.013993			
OTU414 (Muribaculaceae)	0.056772	0.062616			
OTU13 (Muribaculaceae)	0.0351	0.039479			
OTU44 (Candidatus_Arthromitus)	0.002205	0.000134			
OTU9 (Muribaculaceae)	0.092529	0.050961			
OTU87 (Clostridia_UCG-014)	0.065329	0.003803			
OTU94 (Clostridia_UCG-014)	0.000387	0.002792			
OTU11 (Muribaculaceae)	0.086561	0.122445			
OTU10 (Muribaculaceae)	0.059679	0.083717			
OTU23 (Muribaculaceae)	0.033146	0.064402			
OTU56 (Mucispirillum)	0.000276	0.012778			
OTU63 ([Eubacterium]_xylanophilum_group)	0.024745	0.044313			
OTU130 (Lachnospiraceae_NK4A136_group)	0.00066	0.004327			
OTU54 (Lachnospiraceae_NK4A136_group)	0.005284	0.056654			
OTU37 (Lachnospiraceae_unclassified)	0.002343	0.00774			
OTU32 (Muribaculum)	0.033449	0.021311			
OTU34 (Muribaculaceae)	0.022756	0.038678			
OTU45 (Faecalibaculum)	0.000666	0.002165			
OTU15 (Bacteroides)	0.007579	0.030434			
OTU33 (Bacteroides)	0.015014	0.018849			
OTU12 (Muribaculaceae)	0.107735	0.064044			
OTU40 (Muribaculaceae)	0.022636	0.010734			

Data S14. Heatmap of fC group and CPNs group at week 3					
	D1_1_1	D1_9_1	D1_8_1	D1_7_1	D1_6_1
OTU14 (Muribaculaceae)	0.027234	0.04249	0.037284	0.052899	0.048732
OTU8 (Muribaculaceae)	0.051566	0.095735	0.063058	0.043212	0.053554
OTU28 (Muribaculaceae)	0.013782	0.018431	0.016491	0.045751	0.015155
OTU57 (Muribaculaceae)	0.008713	0.004856	0.005751	0.025102	0.008851
OTU21 (Muribaculaceae)	0.029268	0.041865	0.031692	0.076441	0.021552
OTU422 (Muribaculaceae)	0.026743	0.036344	0.037714	0.050116	0.017261
OTU74 (Lachnospiraceae_unclassified)	0.0027	0.049691	0.000644	0.002282	0.00076
OTU41 (Muribaculaceae)	0.024061	0.010086	0.01375	0.213135	0.019775
OTU44 (Candidatus_Arthromitus)	0.024454	0.028757	0.00732	0.009536	0.012521
OTU24 (Muribaculaceae)	0.073211	0.183318	0.123682	0.009289	0.096126
OTU9 (Muribaculaceae)	0.034018	0.063913	0.047959	0.003719	0.041615
OTU5 (Muribaculaceae)	0.078993	0.142713	0.15391	0.08378	0.126846
OTU501 (Muribaculaceae)	0.023234	0.037492	0.039819	0.024873	0.037141
OTU11 (Muribaculaceae)	0.025187	0.011796	0.053226	0.052371	0.027136
OTU77 (Blautia)	0	0.000381	0	0	0
OTU204 (Lachnospiraceae_NK4A136_group)	7.54E-05	0.011886	0	0	0.000364
OTU133 (Lachnospiraceae_unclassified)	0.000437	0.016794	0.000424	0.000785	0.000466
OTU26 (Bifidobacterium)	0	0.00114	0.003159	9.95E-05	0.006344
OTU35 (Lachnospiraceae_unclassified)	0.000411	0	0	0	9.50E-05
OTU27 (Muribaculaceae)	0.004609	0.010133	0.024602	0.000343	0.006606
OTU46 (Muribaculaceae)	0.001641	0.007784	0.029441	0.000391	0.005162
OTU933 (Muribaculaceae)	0.001092	0.006824	0.021101	0.000181	0.004029
OTU6 (Muribaculaceae)	0.038616	0.055144	0.062278	0.020775	0.047831
OTU292 (Muribaculaceae)	0.047038	0.055679	0.057171	0.01902	0.04307
OTU2 (Muribaculaceae)	0.439058	0.058697	0.150171	0.250032	0.347042
OTU32 (Muribaculum)	0.023858	0.00805	0.019355	0.015868	0.011967
	D1_10_1	D1_4_1	D1_3_1	D1_2_1	D1_5_1
OTU14 (Muribaculaceae)	0.038111	0.062185	0.017511	0.020645	0.031598
OTU8 (Muribaculaceae)	0.070933	0.069777	0.114674	0.174762	0.059782
OTU28 (Muribaculaceae)	0.003508	0.025439	0.022391	0.021696	0.009958
OTU57 (Muribaculaceae)	0.004148	0.008906	0.023256	0.011805	0.004764
OTU21 (Muribaculaceae)	0.026338	0.008895	0.043443	0.034975	0.015006
OTU422 (Muribaculaceae)	0.016245	0.039676	0.035748	0.033295	0.014813
OTU74 (Lachnospiraceae_unclassified)	0	0	7.12E-05	7.72E-05	0.000285
OTU41 (Muribaculaceae)	0.009856	0.02922	0.037481	0.026246	0.020791
OTU44 (Candidatus_Arthromitus)	0	0.039722	0.074931	0.050533	0.04049
OTU24 (Muribaculaceae)	0.094269	0.126651	0.173142	0.126423	0.139037
OTU9 (Muribaculaceae)	0.035254	0.039634	0.064315	0.052996	0.05066
OTU5 (Muribaculaceae)	0.137903	0.209413	0.164239	0.197009	0.152911
OTU501 (Muribaculaceae)	0.037771	0.039939	0.037033	0.049633	0.035567
OTU11 (Muribaculaceae)	0.022345	0.057635	0.041144	0.115562	0.019779

OTU77 (Blautia)	0	0.000537	0.000577	0	0.000843
OTU204 (Lachnospiraceae_NK4A136_group)	0	0.000514	0.002442	0.00011	0.000763
OTU133 (Lachnospiraceae_unclassified)	0.000559	0.001078	0.001268	0.000353	0.000761
OTU26 (Bifidobacterium)	0	0.000147	0.000102	0	0.003552
OTU35 (Lachnospiraceae_unclassified)	8.33E-05	0.000943	0.000475	0.000132	0
OTU27 (Muribaculaceae)	0.035623	0.006185	0.007509	0.022019	0.014917
OTU46 (Muribaculaceae)	0.022684	0.007422	0.004792	0.000412	0.004579
OTU933 (Muribaculaceae)	0.0137	0.005161	0.004599	0.00018	0.003142
OTU6 (Muribaculaceae)	0.065994	0.064829	0.03315	0.019454	0.090678
OTU292 (Muribaculaceae)	0.064351	0.059228	0.035351	0.019766	0.095396
OTU2 (Muribaculaceae)	0.290164	0.075081	0.043745	0.016161	0.180384
OTU32 (Muribaculum)	0.010159	0.021782	0.016612	0.005756	0.009546
	D1_3_3	D1_10_3	D1_9_3	D1_8_3	D1_7_3
OTU14 (Muribaculaceae)	0.011526	0.037828	0.024484	0.010622	0.017741
OTU8 (Muribaculaceae)	0.065474	0.047653	0.05632	0.033783	0.059619
OTU28 (Muribaculaceae)	0.004624	0.004449	0.009385	0.000828	0.007316
OTU57 (Muribaculaceae)	0.001375	0.00087	0.002031	0.000199	0.001045
OTU21 (Muribaculaceae)	0.029367	0.014801	0.015101	0.008046	0.013843
OTU422 (Muribaculaceae)	0.020404	0.010378	0.015802	0.003342	0.013488
OTU74 (Lachnospiraceae_unclassified)	0	8.90E-05	0.000159	0.000358	0.000298
OTU41 (Muribaculaceae)	0.005211	0.004509	0.008831	0.002309	0.004442
OTU44 (Candidatus_Arthromitus)	0.000435	0.011631	0.001069	0.000482	0.000569
OTU24 (Muribaculaceae)	0.007473	0.032723	0.038812	0.031944	0.01228
OTU9 (Muribaculaceae)	0.003113	0.015885	0.015844	0.016904	0.005315
OTU5 (Muribaculaceae)	0.049785	0.119904	0.111132	0.076526	0.093218
OTU501 (Muribaculaceae)	0.010769	0.029305	0.028736	0.027684	0.018757
OTU11 (Muribaculaceae)	0.028417	0.051779	0.057206	0.011509	0.135969
OTU77 (Blautia)	0.001742	0.003619	0.006931	0.00046	0.075487
OTU204 (Lachnospiraceae_NK4A136_group)	0.001647	0.004166	0.003241	0.020171	0.027834
OTU133 (Lachnospiraceae_unclassified)	0.001423	0.028056	0.00438	0.049171	0.003421
OTU26 (Bifidobacterium)	0.000183	0.013466	0.004427	0.016325	0.002473
OTU35 (Lachnospiraceae_unclassified)	0.02199	0.046634	0.023405	0.08334	0.003658
OTU27 (Muribaculaceae)	0.008247	0.071906	0.026664	0.027728	0.033108
OTU46 (Muribaculaceae)	0.053201	0.020789	0.012198	0.019555	0.025216
OTU933 (Muribaculaceae)	0.036426	0.014916	0.00972	0.015619	0.0155
OTU6 (Muribaculaceae)	0.106402	0.084064	0.071604	0.060279	0.046044
OTU292 (Muribaculaceae)	0.102345	0.081641	0.072843	0.06344	0.046358
OTU2 (Muribaculaceae)	0.383987	0.225558	0.360056	0.385242	0.317781
OTU32 (Muribaculum)	0.044435	0.02338	0.019618	0.034137	0.019221
	D1_6_3	D1_5_3	D1_4_3	D1_2_3	ND1_10_1
OTU14 (Muribaculaceae)	0.019645	0.008532	0.018482	0.013079	0.030322
OTU8 (Muribaculaceae)	0.043564	0.016684	0.023941	0.010579	0.075119
OTU28 (Muribaculaceae)	0.008808	0.000113	0.006629	9.83E-05	0.010907

OTU57 (Muribaculaceae)	0.001119	0	5.00E-05	0	0.005141
OTU21 (Muribaculaceae)	0.011752	0.004167	0.009513	0.012188	0.018017
OTU422 (Muribaculaceae)	0.011821	0.002152	0.007982	0.004941	0.013265
OTU74 (Lachnospiraceae_unclassified)	0.079321	0.000478	0.0006	3.28E-05	0
OTU41 (Muribaculaceae)	0.006175	0.002007	0.007133	0.009571	0.012103
OTU44 (Candidatus_Arthromitus)	0.000765	9.80E-05	0	0.003414	0.003659
OTU24 (Muribaculaceae)	0.035999	0.012319	0.003534	0.002163	0.075152
OTU9 (Muribaculaceae)	0.01606	0.004302	0.001239	0.000841	0.040803
OTU5 (Muribaculaceae)	0.146221	0.049954	0.108097	0.095047	0.16247
OTU501 (Muribaculaceae)	0.045901	0.016115	0.024744	0.028127	0.062865
OTU11 (Muribaculaceae)	0.025973	0.002775	0.022497	0.030364	0.046965
OTU77 (Blautia)	0.000218	0.000301	0.003707	3.00E-05	0
OTU204 (Lachnospiraceae_NK4A136_group)	0.024165	0.001449	0.009405	0.004829	0.000205
OTU133 (Lachnospiraceae_unclassified)	0.01154	0.000194	0.003403	0.00189	0.000314
OTU26 (Bifidobacterium)	0.003616	0.028201	0.016777	0.066655	0
OTU35 (Lachnospiraceae_unclassified)	0.01615	0.002892	0.002941	0.001393	7.85E-05
OTU27 (Muribaculaceae)	0.039456	0.016576	0.01842	0.023625	0.016749
OTU46 (Muribaculaceae)	0.028549	0.019424	0.015246	0.017785	0.01274
OTU933 (Muribaculaceae)	0.018353	0.014655	0.009948	0.012399	0.010735
OTU6 (Muribaculaceae)	0.064805	0.08231	0.052213	0.061072	0.097625
OTU292 (Muribaculaceae)	0.075029	0.074694	0.055932	0.061834	0.093761
OTU2 (Muribaculaceae)	0.239804	0.601376	0.527312	0.474709	0.185236
OTU32 (Muribaculum)	0.02519	0.038233	0.050257	0.063331	0.025769
	ND1_9_1	ND1_8_1	ND1_7_1	ND1_6_1	ND1_4_1
OTU14 (Muribaculaceae)	0.068001	0.028825	0.026675	0.023233	0.03981
OTU8 (Muribaculaceae)	0.055283	0.060817	0.051761	0.063987	0.078321
OTU28 (Muribaculaceae)	0.014798	0.021158	0.017191	0.013557	0.02902
OTU57 (Muribaculaceae)	0.005448	0.007273	0.008704	0.004289	0.006229
OTU21 (Muribaculaceae)	0.021586	0.021009	0.019259	0.017609	0.025403
OTU422 (Muribaculaceae)	0.016107	0.013306	0.016523	0.01372	0.029655
OTU74 (Lachnospiraceae_unclassified)	0.009961	0.010972	0.025337	0.013875	0.003409
OTU41 (Muribaculaceae)	0.010986	0.016388	0.033633	0.019765	0.017955
OTU44 (Candidatus_Arthromitus)	0.00574	0.019805	0.018917	0.016684	0.036198
OTU24 (Muribaculaceae)	0.070554	0.101715	0.081616	0.057838	0.052024
OTU9 (Muribaculaceae)	0.021881	0.037814	0.035357	0.028872	0.019067
OTU5 (Muribaculaceae)	0.191319	0.148865	0.267241	0.15421	0.159853
OTU501 (Muribaculaceae)	0.031503	0.032199	0.07107	0.052581	0.043912
OTU11 (Muribaculaceae)	0.024659	0.018978	0.044339	0.02639	0.027239
OTU77 (Blautia)	0.000212	0.001084	0.000179	0	0
OTU204 (Lachnospiraceae_NK4A136_group)	5.43E-05	0.067505	0.000659	0.000504	0
OTU133 (Lachnospiraceae_unclassified)	0.001167	0.002044	0.000919	0.001016	0.000598
OTU26 (Bifidobacterium)	0	0	0	0	0
OTU35 (Lachnospiraceae_unclassified)	0.000171	0.00071	0.000334	0.000127	4.20E-05
OTU27 (Muribaculaceae)	0.019423	0.011397	0.011313	0.012808	0.010055

OTU46 (Muribaculaceae)	0.019783	0.006648	0.00467	0.005046	0.004773
OTU933 (Muribaculaceae)	0.014154	0.006917	0.003991	0.003616	0.003145
OTU6 (Muribaculaceae)	0.059067	0.059623	0.064358	0.089753	0.110274
OTU292 (Muribaculaceae)	0.064975	0.060619	0.058988	0.089789	0.104383
OTU2 (Muribaculaceae)	0.260084	0.233035	0.12637	0.274301	0.180913
OTU32 (Muribaculum)	0.013083	0.011295	0.010596	0.01643	0.017722
	ND1_3_1	ND1_2_1	ND1_1_1	ND1_8_3	ND1_7_3
OTU14 (Muribaculaceae)	0.037874	0.140361	0.026937	0.042289	0.032828
OTU8 (Muribaculaceae)	0.054017	0.072568	0.086109	0.184548	0.178543
OTU28 (Muribaculaceae)	0.009928	0.013606	0.018044	0.030978	0.020927
OTU57 (Muribaculaceae)	0.003618	0.006972	0.003384	0.038561	0.024388
OTU21 (Muribaculaceae)	0.012275	0.033625	0.021241	0.035675	0.03618
OTU422 (Muribaculaceae)	0.013031	0.033454	0.015629	0.02554	0.03071
OTU74 (Lachnospiraceae_unclassified)	0.004065	0.000207	7.43E-05	0.000485	0
OTU41 (Muribaculaceae)	0.020773	0.011661	0.012619	0	2.27E-05
OTU44 (Candidatus_Arthromitus)	0.003022	0.015551	0.00558	0.001308	0.000647
OTU24 (Muribaculaceae)	0.059043	0.105486	0.058266	0.034495	0.034251
OTU9 (Muribaculaceae)	0.025295	0.042194	0.030702	0.015655	0.01307
OTU5 (Muribaculaceae)	0.140557	0.21837	0.176782	0.116702	0.068482
OTU501 (Muribaculaceae)	0.045852	0.04494	0.068879	0.030212	0.014719
OTU11 (Muribaculaceae)	0.029579	0.025919	0.031664	0.071003	0.135867
OTU77 (Blautia)	0	0	0	0.000374	0.003348
OTU204 (Lachnospiraceae_NK4A136_group)	0	0.0001	0.000152	0.048311	0.003166
OTU133 (Lachnospiraceae_unclassified)	0.000284	0.001648	0.000499	0.008577	0.000999
OTU26 (Bifidobacterium)	0	0	0	0.008178	0.083016
OTU35 (Lachnospiraceae_unclassified)	0	0.000805	5.94E-05	0.047641	0.022017
OTU27 (Muribaculaceae)	0.006246	0.012704	0.014927	0.062711	0.054523
OTU46 (Muribaculaceae)	0.009914	0.008947	0.011499	0.032464	0.035783
OTU933 (Muribaculaceae)	0.006904	0.005913	0.008073	0.022575	0.022437
OTU6 (Muribaculaceae)	0.076052	0.036009	0.097504	0.065035	0.085335
OTU292 (Muribaculaceae)	0.071048	0.031619	0.08646	0.065526	0.089046
OTU2 (Muribaculaceae)	0.346585	0.117537	0.214071	0.006503	0.002818
OTU32 (Muribaculum)	0.024039	0.019806	0.010845	0.004654	0.006877
	ND1_6_3	ND1_1_3	ND1_3_3	ND1_2_3	ND1_9_3
OTU14 (Muribaculaceae)	0.051799	0.011599	0.017518	0.019393	0.042715
OTU8 (Muribaculaceae)	0.184528	0.039765	0.082275	0.061313	0.162565
OTU28 (Muribaculaceae)	0.019949	0.005084	0.008443	0.010038	0.024605
OTU57 (Muribaculaceae)	0.059002	0.00196	0.001307	0.001357	0.02488
OTU21 (Muribaculaceae)	0.026113	0.020607	0.013614	0.011067	0.031972
OTU422 (Muribaculaceae)	0.022987	0.011987	0.008039	0.008088	0.024851
OTU74 (Lachnospiraceae_unclassified)	0.001184	0	0.003901	0.00327	5.80E-05
OTU41 (Muribaculaceae)	0	0.003538	0.005956	0.006853	0
OTU44 (Candidatus_Arthromitus)	0.002231	0.004825	0.000556	0.000613	0.001795

OTU24 (Muribaculaceae)	0.046107	0.012678	0.014805	0.016914	0.023825
OTU9 (Muribaculaceae)	0.021053	0.007485	0.005132	0.007074	0.010493
OTU5 (Muribaculaceae)	0.096079	0.088578	0.128364	0.147168	0.097486
OTU501 (Muribaculaceae)	0.029436	0.032704	0.034767	0.046131	0.024224
OTU11 (Muribaculaceae)	0.133689	0.061386	0.072723	0.076916	0.142758
OTU77 (Blautia)	0.004165	0	0	1.70E-05	0.010449
OTU204 (Lachnospiraceae_NK4A136_group)	0.021784	0.002663	0.001276	0.003041	0.010624
OTU133 (Lachnospiraceae_unclassified)	0.002912	0.004223	0.00326	0.003109	0.009021
OTU26 (Bifidobacterium)	0.056383	7.68E-05	0.00184	0.003261	0.049822
OTU35 (Lachnospiraceae_unclassified)	0.003986	0.001464	0.004861	0.029177	0.076917
OTU27 (Muribaculaceae)	0.036578	0.021592	0.061994	0.051427	0.072182
OTU46 (Muribaculaceae)	0.019138	0.022738	0.031747	0.029115	0.03038
OTU933 (Muribaculaceae)	0.012213	0.01569	0.019428	0.021233	0.022944
OTU6 (Muribaculaceae)	0.068828	0.055499	0.080977	0.079536	0.047142
OTU292 (Muribaculaceae)	0.068873	0.052037	0.08492	0.075749	0.045554
OTU2 (Muribaculaceae)	0.002456	0.500656	0.278406	0.252286	0.002766
OTU32 (Muribaculum)	0.008528	0.021164	0.033892	0.035855	0.009971
	ND1_4_3	ND1_10_3			
OTU14 (Muribaculaceae)	0.01726	0.048885			
OTU8 (Muribaculaceae)	0.047616	0.135785			
OTU28 (Muribaculaceae)	0.007385	0.022142			
OTU57 (Muribaculaceae)	0.000865	0.038069			
OTU21 (Muribaculaceae)	0.013374	0.036025			
OTU422 (Muribaculaceae)	0.00647	0.030263			
OTU74 (Lachnospiraceae_unclassified)	0	0			
OTU41 (Muribaculaceae)	0.003967	0.000108			
OTU44 (Candidatus_Arthromitus)	0.001091	0.00095			
OTU24 (Muribaculaceae)	0.016416	0.032631			
OTU9 (Muribaculaceae)	0.006245	0.012969			
OTU5 (Muribaculaceae)	0.097823	0.116402			
OTU501 (Muribaculaceae)	0.021026	0.031487			
OTU11 (Muribaculaceae)	0.065254	0.10171			
OTU77 (Blautia)	0	0.002152			
OTU204 (Lachnospiraceae_NK4A136_group)	0.000206	0.000423			
OTU133 (Lachnospiraceae_unclassified)	0.00097	0.007941			
OTU26 (Bifidobacterium)	0.006541	0.037488			
OTU35 (Lachnospiraceae_unclassified)	0.001028	0.102997			
OTU27 (Muribaculaceae)	0.038412	0.053409			
OTU46 (Muribaculaceae)	0.022837	0.030079			
OTU933 (Muribaculaceae)	0.015783	0.022989			
OTU6 (Muribaculaceae)	0.070578	0.061543			
OTU292 (Muribaculaceae)	0.069899	0.064829			
OTU2 (Muribaculaceae)	0.440211	0.002624			
OTU32 (Muribaculum)	0.028741	0.006099			

Data S15. KEGG pathway of different metabolites in fC group at different time points			
meta	CID	pathway	pathway_name
L-Tyrosine	C00082	mmu05031	Amphetamine addiction
L-Asparagine	C00152	mmu00250	Alanine, aspartate and glutamate metabolism
Sphinganine	C00836	mmu04071	Sphingolipid signaling pathway
SM(d18:0/16:1(9Z))	C00550	mmu04071	Sphingolipid signaling pathway
D-Pantothenic acid	C00864	mmu04977	Vitamin digestion and absorption
12(R)-HETE	C14822	mmu04925	Aldosterone synthesis and secretion
Aldosterone	C01780	mmu04925	Aldosterone synthesis and secretion
5'-Deoxy-5-fluorouridine	C12739	mmu00983	Drug metabolism - other enzymes
L-Tyrosine	C00082	mmu05034	Alcoholism
1-Butanol	C06142	mmu00650	Butanoate metabolism
L-Tyrosine	C00082	mmu00360	Phenylalanine metabolism
L-Ornithine	C00077	mmu00330	Arginine and proline metabolism
Fructose 1-phosphate	C01094	mmu00051	Fructose and mannose metabolism
Xanthurenic Acid	C02470	mmu00380	Tryptophan metabolism
5-Hydroxyindoleacetic acid	C05635	mmu00380	Tryptophan metabolism
5-Hydroxyindoleacetic acid	C05635	mmu04726	Serotonergic synapse
Orotic acid	C00295	mmu00240	Pyrimidine metabolism
12(R)-HETE	C14822	mmu00590	Arachidonic acid metabolism
Cortisol	C00735	mmu04934	Cushing syndrome
D-Pantothenic acid	C00864	mmu00770	Pantothenate and CoA biosynthesis
L-Tyrosine	C00082	mmu01210	2-Oxocarboxylic acid metabolism
L-Ornithine	C00077	mmu01210	2-Oxocarboxylic acid metabolism
L-Tyrosine	C00082	mmu05012	Parkinson disease
Dodecanoic acid	C02679	mmu00061	Fatty acid biosynthesis
L-Tyrosine	C00082	mmu00730	Thiamine metabolism
Uric acid	C00366	mmu04976	Bile secretion
Phenethylamine glucuronide	C03033	mmu04976	Bile secretion
2-Phenylethanol glucuronide	C03033	mmu04976	Bile secretion
Glycochenodeoxycholic acid 3-glucuronide	C03033	mmu04976	Bile secretion
Estrone	C02538	mmu04976	Bile secretion
Trigonellinamide	C02918	mmu04976	Bile secretion

Cortisol	C00735	mmu04976	Bile secretion
Deoxycholic acid	C04483	mmu04976	Bile secretion
L-Tyrosine	C00082	mmu04917	Prolactin signaling pathway
L-Asparagine	C00152	mmu04978	Mineral absorption
L-Histidine	C00135	mmu01230	Biosynthesis of amino acids
L-Asparagine	C00152	mmu01230	Biosynthesis of amino acids
L-Tyrosine	C00082	mmu01230	Biosynthesis of amino acids
L-Ornithine	C00077	mmu01230	Biosynthesis of amino acids
Trigonellinamide	C02918	mmu00760	Nicotinate and nicotinamide metabolism
Phenethylamine glucuronide	C03033	mmu00040	Pentose and glucuronate interconversions
2-Phenylethanol glucuronide	C03033	mmu00040	Pentose and glucuronate interconversions
Glycochenodeoxycholic acid 3-glucuronide	C03033	mmu00040	Pentose and glucuronate interconversions
L-Histidine	C00135	mmu00410	beta-Alanine metabolism
D-Pantothenic acid	C00864	mmu00410	beta-Alanine metabolism
Cortisol	C00735	mmu04080	Neuroactive ligand-receptor interaction
Sphinganine-phosphate	C01120	mmu04080	Neuroactive ligand-receptor interaction
Cortisol	C00735	mmu05215	Prostate cancer
Cortisol	C00735	mmu00140	Steroid hormone biosynthesis
Estrone	C02538	mmu00140	Steroid hormone biosynthesis
Aldosterone	C01780	mmu00140	Steroid hormone biosynthesis
Xanthine	C00385	mmu00232	Caffeine metabolism
Xanthosine	C01762	mmu00232	Caffeine metabolism
L-Tyrosine	C00082	mmu05030	Cocaine addiction
L-Ornithine	C00077	mmu00480	Glutathione metabolism
Carboxyphosphamide	C07646	mmu00982	Drug metabolism - cytochrome P450
L-Tyrosine	C00082	mmu04916	Melanogenesis
L-Ornithine	C00077	mmu00220	Arginine biosynthesis
L-Histidine	C00135	mmu00340	Histidine metabolism
L-Histidine trimethylbetaine	C05575	mmu00340	Histidine metabolism
L-Ornithine	C00077	mmu00472	D-Arginine and D-ornithine metabolism
Melibiitol	C05399	mmu00052	Galactose metabolism
Cortisol	C00735	mmu05200	Pathways in cancer
Inosine	C00294	mmu02010	ABC transporters

Xanthosine	C01762	mmu02010	ABC transporters
L-Histidine	C00135	mmu02010	ABC transporters
L-Ornithine	C00077	mmu02010	ABC transporters
L-Histidine	C00135	mmu04974	Protein digestion and absorption
L-Asparagine	C00152	mmu04974	Protein digestion and absorption
L-Tyrosine	C00082	mmu04974	Protein digestion and absorption
SM(d18:0/16:1(9Z))	C00550	mmu04217	Necroptosis
L-Tyrosine	C00082	mmu00130	Ubiquinone and other terpenoid-quinone biosynthesis
Quinic acid	C00296	mmu00400	Phenylalanine, tyrosine and tryptophan biosynthesis
Fructose 1-phosphate	C01094	mmu00400	Phenylalanine, tyrosine and tryptophan biosynthesis
L-Tyrosine	C00082	mmu00400	Phenylalanine, tyrosine and tryptophan biosynthesis
Sphinganine	C00836	mmu00600	Sphingolipid metabolism
3-O-Sulfogalactosylceramide (d18:1/16:0)	C06125	mmu00600	Sphingolipid metabolism
3-O-Sulfogalactosylceramide (d18:1/22:0)	C06125	mmu00600	Sphingolipid metabolism
Sphinganine-phosphate	C01120	mmu00600	Sphingolipid metabolism
SM(d18:0/16:1(9Z))	C00550	mmu00600	Sphingolipid metabolism
Dihydrobiopterin	C02953	mmu00790	Folate biosynthesis
L-Histidine	C00135	mmu05230	Central carbon metabolism in cancer
L-Asparagine	C00152	mmu05230	Central carbon metabolism in cancer
L-Tyrosine	C00082	mmu05230	Central carbon metabolism in cancer
Cortisol	C00735	mmu04960	Aldosterone-regulated sodium reabsorption
Aldosterone	C01780	mmu04960	Aldosterone-regulated sodium reabsorption
Uric acid	C00366	mmu01100	Metabolic pathways
Genistein	C06563	mmu01100	Metabolic pathways
Aldosterone	C01780	mmu01100	Metabolic pathways
Abscisic acid	C06082	mmu01100	Metabolic pathways
D-Pantothenic acid	C00864	mmu01100	Metabolic pathways
Sphinganine-phosphate	C01120	mmu01100	Metabolic pathways
Phenethylamine glucuronide	C03033	mmu01100	Metabolic pathways
2-Phenylethanol glucuronide	C03033	mmu01100	Metabolic pathways
Glycochenodeoxycholic acid 3-glucuronide	C03033	mmu01100	Metabolic pathways
1-Butanol	C06142	mmu01100	Metabolic pathways
3-O-Sulfogalactosylceramide (d18:1/16:0)	C06125	mmu01100	Metabolic pathways

3-O-Sulfogalactosylceramide (d18:1/22:0)	C06125	mmu01100	Metabolic pathways
O-Cresol	C01542	mmu01100	Metabolic pathways
SM(d18:0/16:1(9Z))	C00550	mmu01100	Metabolic pathways
Dodecanoic acid	C02679	mmu01100	Metabolic pathways
Quinic acid	C00296	mmu01100	Metabolic pathways
Inosine	C00294	mmu01100	Metabolic pathways
Trigonellinamide	C02918	mmu01100	Metabolic pathways
L-Histidine	C00135	mmu01100	Metabolic pathways
L-Asparagine	C00152	mmu01100	Metabolic pathways
L-Ornithine	C00077	mmu01100	Metabolic pathways
5-Hydroxyindoleacetic acid	C05635	mmu01100	Metabolic pathways
Orotic acid	C00295	mmu01100	Metabolic pathways
Dihydrobiopterin	C02953	mmu01100	Metabolic pathways
L-Histidine trimethylbetaine	C05575	mmu01100	Metabolic pathways
Xanthosine	C01762	mmu01100	Metabolic pathways
L-Tyrosine	C00082	mmu01100	Metabolic pathways
Sphinganine	C00836	mmu01100	Metabolic pathways
Xanthine	C00385	mmu01100	Metabolic pathways
12(R)-HETE	C14822	mmu01100	Metabolic pathways
Cortisol	C00735	mmu01100	Metabolic pathways
4-Hydroxy-3-methoxycinnamaldehyde	C02666	mmu01100	Metabolic pathways
S-Lactoylglutathione	C03451	mmu01100	Metabolic pathways
Hypoxanthine	C00262	mmu01100	Metabolic pathways
Fructose 1-phosphate	C01094	mmu01100	Metabolic pathways
Lactosamine	C00203	mmu01100	Metabolic pathways
Lactosamine	C00203	mmu00520	Amino sugar and nucleotide sugar metabolism
Uric acid	C00366	mmu00230	Purine metabolism
Xanthine	C00385	mmu00230	Purine metabolism
Inosine	C00294	mmu00230	Purine metabolism
Xanthosine	C01762	mmu00230	Purine metabolism
Hypoxanthine	C00262	mmu00230	Purine metabolism
L-Histidine	C00135	mmu00970	Aminoacyl-tRNA biosynthesis
L-Asparagine	C00152	mmu00970	Aminoacyl-tRNA biosynthesis

L-Tyrosine	C00082	mmu00970	Aminoacyl-tRNA biosynthesis
Cortisol	C00735	mmu04927	Cortisol synthesis and secretion
S-Lactoylglutathione	C03451	mmu00620	Pyruvate metabolism
L-Tyrosine	C00082	mmu04728	Dopaminergic synapse
L-Tyrosine	C00082	mmu00350	Tyrosine metabolism

**Data S16.** **KEGG pathway of different metabolites between fC group and CPNs group at week 2**			
	CID	pathway	pathway_name
LysoPC(24:1(15Z))	C04230	mmu05231	Choline metabolism in cancer
L-Tyrosine	C00082	mmu05031	Amphetamine addiction
D-Pantothenic acid	C00864	mmu04977	Vitamin digestion and absorption
Pantothenic Acid	C00864	mmu04977	Vitamin digestion and absorption
Aldosterone	C01780	mmu04925	Aldosterone synthesis and secretion
L-Tyrosine	C00082	mmu05034	Alcoholism
1-Butanol	C06142	mmu00650	Butanoate metabolism
L-Tyrosine	C00082	mmu00360	Phenylalanine metabolism
L-Ornithine	C00077	mmu00330	Arginine and proline metabolism
Spermidine	C00315	mmu00330	Arginine and proline metabolism
Fructose 1-phosphate	C01094	mmu00051	Fructose and mannose metabolism
Xanthurenic Acid	C02470	mmu00380	Tryptophan metabolism
5-Hydroxyindoleacetic acid	C05635	mmu00380	Tryptophan metabolism
Kynurenic acid	C01717	mmu00380	Tryptophan metabolism
5-Hydroxyindoleacetic acid	C05635	mmu04726	Serotonergic synapse
Cortisol	C00735	mmu04934	Cushing syndrome
D-Pantothenic acid	C00864	mmu00770	Pantothenate and CoA biosynthesis
Pantothenic Acid	C00864	mmu00770	Pantothenate and CoA biosynthesis
L-Ornithine	C00077	mmu01210	2-Oxocarboxylic acid metabolism
L-Tyrosine	C00082	mmu01210	2-Oxocarboxylic acid metabolism
L-Tyrosine	C00082	mmu05012	Parkinson disease
L-Tyrosine	C00082	mmu00730	Thiamine metabolism
Uric acid	C00366	mmu04976	Bile secretion
Cortisol	C00735	mmu04976	Bile secretion
2-Phenylethanol glucuronide	C03033	mmu04976	Bile secretion
Glycochenodeoxycholic acid 3-glucuronide	C03033	mmu04976	Bile secretion
Spermidine	C00315	mmu04976	Bile secretion
L-Tyrosine	C00082	mmu04917	Prolactin signaling pathway
L-Ornithine	C00077	mmu01230	Biosynthesis of amino acids
L-Tyrosine	C00082	mmu01230	Biosynthesis of amino acids
2-Phenylethanol glucuronide	C03033	mmu00040	Pentose and glucuronate interconversions

Glycochenodeoxycholic acid 3-glucuronide	C03033	mmu00040	Pentose and glucuronate interconversions
D-Pantothenic acid	C00864	mmu00410	beta-Alanine metabolism
Pantothenic Acid	C00864	mmu00410	beta-Alanine metabolism
Spermidine	C00315	mmu00410	beta-Alanine metabolism
Cortisol	C00735	mmu04080	Neuroactive ligand-receptor interaction
Sphinganine-phosphate	C01120	mmu04080	Neuroactive ligand-receptor interaction
Cortisol	C00735	mmu05215	Prostate cancer
Cortisol	C00735	mmu00140	Steroid hormone biosynthesis
21-Deoxycortisol	C05497	mmu00140	Steroid hormone biosynthesis
Aldosterone	C01780	mmu00140	Steroid hormone biosynthesis
Xanthosine	C01762	mmu00232	Caffeine metabolism
L-Tyrosine	C00082	mmu05030	Cocaine addiction
L-Ornithine	C00077	mmu00480	Glutathione metabolism
Spermidine	C00315	mmu00480	Glutathione metabolism
Carboxyphosphamide	C07646	mmu00982	Drug metabolism - cytochrome P450
L-Tyrosine	C00082	mmu04916	Melanogenesis
L-Ornithine	C00077	mmu00220	Arginine biosynthesis
Formimino-L-glutamic acid	C00439	mmu00340	Histidine metabolism
L-Ornithine	C00077	mmu00472	D-Arginine and D-ornithine metabolism
Cortisol	C00735	mmu05200	Pathways in cancer
Inosine	C00294	mmu02010	ABC transporters
Xanthosine	C01762	mmu02010	ABC transporters
L-Ornithine	C00077	mmu02010	ABC transporters
Spermidine	C00315	mmu02010	ABC transporters
L-Tyrosine	C00082	mmu04974	Protein digestion and absorption
L-Tyrosine	C00082	mmu00130	Ubiquinone and other terpenoid-quinone biosynthesis
Quinic acid	C00296	mmu00400	Phenylalanine, tyrosine and tryptophan biosynthesis
Fructose 1-phosphate	C01094	mmu00400	Phenylalanine, tyrosine and tryptophan biosynthesis
L-Tyrosine	C00082	mmu00400	Phenylalanine, tyrosine and tryptophan biosynthesis
3-O-Sulfogalactosylceramide (d18:1/22:0)	C06125	mmu00600	Sphingolipid metabolism
Sphinganine-phosphate	C01120	mmu00600	Sphingolipid metabolism
DL-Pipecolinic acid	C00408	mmu00310	Lysine degradation
L-Tyrosine	C00082	mmu05230	Central carbon metabolism in cancer

Cortisol	C00735	mmu04960	Aldosterone-regulated sodium reabsorption
Aldosterone	C01780	mmu04960	Aldosterone-regulated sodium reabsorption
Uric acid	C00366	mmu01100	Metabolic pathways
Genistein	C06563	mmu01100	Metabolic pathways
Aldosterone	C01780	mmu01100	Metabolic pathways
Abscisic acid	C06082	mmu01100	Metabolic pathways
D-Pantothenic acid	C00864	mmu01100	Metabolic pathways
Pantothenic Acid	C00864	mmu01100	Metabolic pathways
DL-Pipecolinic acid	C00408	mmu01100	Metabolic pathways
Sphinganine-phosphate	C01120	mmu01100	Metabolic pathways
2-Phenylethanol glucuronide	C03033	mmu01100	Metabolic pathways
Glycochenodeoxycholic acid 3-glucuronide	C03033	mmu01100	Metabolic pathways
1-Butanol	C06142	mmu01100	Metabolic pathways
Formimino-L-glutamic acid	C00439	mmu01100	Metabolic pathways
3-O-Sulfogalactosylceramide (d18:1/22:0)	C06125	mmu01100	Metabolic pathways
Spermidine	C00315	mmu01100	Metabolic pathways
Kynurenic acid	C01717	mmu01100	Metabolic pathways
Quinic acid	C00296	mmu01100	Metabolic pathways
Inosine	C00294	mmu01100	Metabolic pathways
L-Ornithine	C00077	mmu01100	Metabolic pathways
Xanthosine	C01762	mmu01100	Metabolic pathways
5-Hydroxyindoleacetic acid	C05635	mmu01100	Metabolic pathways
Guanine	C00242	mmu01100	Metabolic pathways
L-Tyrosine	C00082	mmu01100	Metabolic pathways
Hypoxanthine	C00262	mmu01100	Metabolic pathways
Cortisol	C00735	mmu01100	Metabolic pathways
4-Hydroxy-3-methoxycinnamaldehyde	C02666	mmu01100	Metabolic pathways
Fructose 1-phosphate	C01094	mmu01100	Metabolic pathways
Uric acid	C00366	mmu00230	Purine metabolism
Hypoxanthine	C00262	mmu00230	Purine metabolism
Inosine	C00294	mmu00230	Purine metabolism
Guanine	C00242	mmu00230	Purine metabolism
Xanthosine	C01762	mmu00230	Purine metabolism

L-Tyrosine	C00082	mmu00970	Aminoacyl-tRNA biosynthesis
Cortisol	C00735	mmu04927	Cortisol synthesis and secretion
L-Tyrosine	C00082	mmu04728	Dopaminergic synapse
L-Tyrosine	C00082	mmu00350	Tyrosine metabolism
LysoPC(24:1(15Z))	C04230	mmu00564	Glycerophospholipid metabolism

**Data S17. KEGG pathway of different metabolites between fC group and CPNs group at week 3**			
meta	CID	pathway	pathway_name
PC(16:0/20:5(5Z,8Z,11Z,14Z,17Z))	C00157	mmu05231	Choline metabolism in cancer
Sphinganine	C00836	mmu04071	Sphingolipid signaling pathway
SM(d18:0/16:1(9Z))	C00550	mmu04071	Sphingolipid signaling pathway
D-Pantothenic acid	C00864	mmu04977	Vitamin digestion and absorption
5'-Deoxy-5-fluorouridine	C12739	mmu00983	Drug metabolism - other enzymes
PC(16:0/20:5(5Z,8Z,11Z,14Z,17Z))	C00157	mmu00592	alpha-Linolenic acid metabolism
Hippurate	C01586	mmu00360	Phenylalanine metabolism
L-Ornithine	C00077	mmu00330	Arginine and proline metabolism
N-Acetyl-L-glutamate 5-semialdehyde	C01250	mmu00330	Arginine and proline metabolism
AFMK	C05642	mmu00380	Tryptophan metabolism
PC(16:0/20:5(5Z,8Z,11Z,14Z,17Z))	C00157	mmu00590	Arachidonic acid metabolism
Betaine	C00719	mmu00260	Glycine, serine and threonine metabolism
D-Pantothenic acid	C00864	mmu00770	Pantothenate and CoA biosynthesis
Dexpanthenol	C05944	mmu00770	Pantothenate and CoA biosynthesis
L-Ornithine	C00077	mmu01210	2-Oxocarboxylic acid metabolism
N-Acetyl-L-glutamate 5-semialdehyde	C01250	mmu01210	2-Oxocarboxylic acid metabolism
Uric acid	C00366	mmu04976	Bile secretion
Phenethylamine glucuronide	C03033	mmu04976	Bile secretion
Estrone	C02538	mmu04976	Bile secretion
L-Ornithine	C00077	mmu01230	Biosynthesis of amino acids
N-Acetyl-L-glutamate 5-semialdehyde	C01250	mmu01230	Biosynthesis of amino acids
Trigonelline	C01004	mmu00760	Nicotinate and nicotinamide metabolism
Phenethylamine glucuronide	C03033	mmu00040	Pentose and glucuronate interconversions
D-Pantothenic acid	C00864	mmu00410	beta-Alanine metabolism
Estrone	C02538	mmu00140	Steroid hormone biosynthesis
Xanthine	C00385	mmu00232	Caffeine metabolism
Xanthosine	C01762	mmu00232	Caffeine metabolism
PC(16:0/20:5(5Z,8Z,11Z,14Z,17Z))	C00157	mmu00591	Linoleic acid metabolism
L-Ornithine	C00077	mmu00480	Glutathione metabolism
Carboxyphosphamide	C07646	mmu00982	Drug metabolism - cytochrome P450
L-Ornithine	C00077	mmu00220	Arginine biosynthesis
N-Acetyl-L-glutamate 5-semialdehyde	C01250	mmu00220	Arginine biosynthesis
1-Nitro-5-glutathionyl-6-hydroxy-5,6-dihydronaphthalene	C14806	mmu00980	Metabolism of xenobiotics by cytochrome P450
L-Ornithine	C00077	mmu00472	D-Arginine and D-ornithine metabolism
Inosine	C00294	mmu02010	ABC transporters
Xanthosine	C01762	mmu02010	ABC transporters
L-Ornithine	C00077	mmu02010	ABC transporters
Betaine	C00719	mmu02010	ABC transporters
SM(d18:0/16:1(9Z))	C00550	mmu04217	Necroptosis
Quinic acid	C00296	mmu00400	Phenylalanine, tyrosine and tryptophan biosynthesis

Sphinganine	C00836	mmu00600	Sphingolipid metabolism
3-O-Sulfogalactosylceramide (d18:1/16:0)	C06125	mmu00600	Sphingolipid metabolism
3-O-Sulfogalactosylceramide (d18:1/22:0)	C06125	mmu00600	Sphingolipid metabolism
SM(d18:0/16:1(9Z))	C00550	mmu00600	Sphingolipid metabolism
DL-Pipecolinic acid	C00408	mmu00310	Lysine degradation
Dihydrobiopterin	C02953	mmu00790	Folate biosynthesis
Uric acid	C00366	mmu01100	Metabolic pathways
Genistein	C06563	mmu01100	Metabolic pathways
Abscisic acid	C06082	mmu01100	Metabolic pathways
D-Pantothenic acid	C00864	mmu01100	Metabolic pathways
Phenethylamine glucuronide	C03033	mmu01100	Metabolic pathways
Hippurate	C01586	mmu01100	Metabolic pathways
3-O-Sulfogalactosylceramide (d18:1/16:0)	C06125	mmu01100	Metabolic pathways
3-O-Sulfogalactosylceramide (d18:1/22:0)	C06125	mmu01100	Metabolic pathways
Ferulic acid	C01494	mmu01100	Metabolic pathways
Pyrocatechol	C00090	mmu01100	Metabolic pathways
SM(d18:0/16:1(9Z))	C00550	mmu01100	Metabolic pathways
Quinic acid	C00296	mmu01100	Metabolic pathways
Inosine	C00294	mmu01100	Metabolic pathways
L-Ornithine	C00077	mmu01100	Metabolic pathways
PC(16:0/20:5(5Z,8Z,11Z,14Z,17Z))	C00157	mmu01100	Metabolic pathways
Xanthosine	C01762	mmu01100	Metabolic pathways
Dihydrobiopterin	C02953	mmu01100	Metabolic pathways
DL-Pipecolinic acid	C00408	mmu01100	Metabolic pathways
Sphinganine	C00836	mmu01100	Metabolic pathways
Xanthine	C00385	mmu01100	Metabolic pathways
4-Hydroxy-3-methoxycinnamaldehyde	C02666	mmu01100	Metabolic pathways
N-Acetyl-L-glutamate 5-semialdehyde	C01250	mmu01100	Metabolic pathways
Betaine	C00719	mmu01100	Metabolic pathways
Caffeic Acid	C01197	mmu01100	Metabolic pathways
Lactosamine	C00203	mmu01100	Metabolic pathways
Lactosamine	C00203	mmu00520	Amino sugar and nucleotide sugar metabolism
Uric acid	C00366	mmu00230	Purine metabolism
Xanthine	C00385	mmu00230	Purine metabolism
Inosine	C00294	mmu00230	Purine metabolism
Xanthosine	C01762	mmu00230	Purine metabolism
All-trans-heptaprenyl diphosphate	C04216	mmu00900	Terpenoid backbone biosynthesis
PC(16:0/20:5(5Z,8Z,11Z,14Z,17Z))	C00157	mmu04723	Retrograde endocannabinoid signaling
PC(16:0/20:5(5Z,8Z,11Z,14Z,17Z))	C00157	mmu00564	Glycerophospholipid metabolism
